# Supplementary material for: Exploring CRISPR-Cas: The transformative impact of gene editing in molecular biology
Source: Mol Ther Nucleic Acids. 2025 Sep 15;36(4):102717. doi: 10.1016/j.omtn.2025.102717 (PMC12506487; doi:10.1016/j.omtn.2025.102717)
Supplement: Document S1. Tables S1 and S2 [file mmc1.pdf]

OMTN, Volume *36*

Supplemental information

Exploring CRISPR-Cas: The transformative impact of gene editing in molecular biology

Vivek Pandey, Shivani Sharma, and Yuba Raj Pokharel

Tables:

Table S1: Cancer-Lethal Genes by CRISPR Screen Type: Loss-of-Function and Gain-of-Function

Sr. No.	Gene	Function/Pathway	Cancer Type	Screen Outcome	Therapeutic Implication
1	BCR-ABL [1]	Tyrosine kinase fusion	Chronic myeloid leukemia	Essential - LOF causes cell death	Direct target for imatinib therapy
2	KRAS [2]	Small GTPase/MAPK signaling	Colorectal, pancreatic cancer	Essential - LOF inhibits growth	Synthetic lethal targets identified
3	Anti-proliferative genes [3]	Growth suppression	K562 leukemia	GOF - activation inhibits growth	Reactivation therapy strategies
4	MAPK pathway genes [4]	Growth signaling	A375 melanoma	GOF - activation confers drug resistance	Combination MAPK inhibition
5	lncRNAs (499 identified) [5]	Gene regulation	Multiple cell lines	Cell-specific effects	lncRNA-targeted therapies
6	PVT1 [6]	Pro-growth lncRNA	Various cancer cells	Silencing - reduces growth	lncRNA modulation
7	CDK6 [2]	Etoposide	HL60 leukemia	LOF - alters cell cycle response	CDK inhibitors
8	NF2 [7]	BRAF inhibitors	A375 melanoma	LOF - activates alternative pathways	Hippo pathway targeting
9	TRIM37 [8]	PLK4 inhibitors	Multiple cancers	LOF - synthetic lethality	Biomarker-driven therapy
10	ARID1A [9]	Chromatin remodeling	LOF	Enhances T cell persistence	Epigenetic modulators
11	SLC7A1/SLC38A2 [10]	Amino acid transport	LOF	Affects T cell metabolism	Metabolic modulators

Table S2: CRISPR Clinical Trials - Therapeutic Potential and Global Progress

Sr. No.	Trial/Therapy	CRISPR Technique	Disease Target	Country	Phase	Sample Size	Primary Outcome	Status/Results
1	CTX001 (Casgevy) [11]	Base editing (BCL11A)	Sickle cell disease, β-thalassemia	USA, Europe, Canada	III	75 patients	Transfusion independence	FDA/EMA Approved 2023 95% success
2	EDIT-101[12]	Cas9 knockout (CEP290)	Leber congenital amaurosis	USA	I/II	18 patients	Vision restoration	Ongoing - promising early results
3	CTX110 [13]	Cas9 (TRAC knockout)	B-cell malignancies	USA, Europe	I	37 patients	CAR-T efficacy	70% overall response rate
4	NTLA-2001 [14]	*In vivo* base editing	Hereditary ATTR amyloidosis	USA, UK	I	12 patients	TTR protein reduction	87% reduction achieved
5	Universal CAR-T [15]	Cas9 multiplex editing	ALL, lymphoma	UK, USA	I	28 patients	Remission rates	60% complete remission
6	CRISPR-HIV (CCR5)[16,17]	Cas9 knockout	HIV infection	China, USA	I	24 patients	Viral suppression	Mixed results, ongoing studies

7	AGN-151587 [18]	Cas9 editing	Geographic atrophy (AMD)	USA	I/II	79 patients	Vision preservation	Recruiting patients
8	CRISPR-001 [11]	*Ex vivo* editing	β-thalassemia	Multiple	I/II	42 patients	Hemoglobin increase	89% transfusion reduction
9	NY-ESO-1 TCR [19]	Cas9 multiplex	Solid tumors	USA	I	20 patients	Tumor response	50% stable disease
10	Base Editor DMD [20]	Cytosine base editing	Duchenne muscular dystrophy	USA	Preclinical	N/A	Dystrophin restoration	In development

References:

(1) Hart, T.; Chandrashekhar, M.; Aregger, M.; Steinhart, Z.; Brown, K. R.; MacLeod, G.; Mis, M.; Zimmermann, M.; Fradet-Turcotte, A.; Sun, S.; Mero, P.; Dirks, P.; Sidhu, S.; Roth, F. P.; Rissland, O. S.; Durocher, D.; Angers, S.; Moffat, J. High-Resolution CRISPR Screens Reveal Fitness Genes and Genotype-Specific Cancer Liabilities. *Cell* **2015**, *163* (6), 1515–1526. https://doi.org/10.1016/J.CELL.2015.11.015.

(2) Wang, T.; Wei, J. J.; Sabatini, D. M.; Lander, E. S. Genetic Screens in Human Cells Using the CRISPR-Cas9 System. *Science (80-.).* **2014**, *343* (6166), 80–84. https://doi.org/10.1126/SCIENCE.1246981.

(3) Gilbert, L. A.; Horlbeck, M. A.; Adamson, B.; Villalta, J. E.; Chen, Y.; Whitehead, E. H.; Guimaraes, C.; Panning, B.; Ploegh, H. L.; Bassik, M. C.; Qi, L. S.; Kampmann, M.; Weissman, J. S. Genome-Scale CRISPR-Mediated Control of Gene Repression and Activation. *Cell* **2014**,

159 (3), 647–661. https://doi.org/10.1016/J.CELL.2014.09.029.

(4) Konermann, S.; Brigham, M. D.; Trevino, A. E.; Joung, J.; Abudayyeh, O. O.; Barcena, C.; Hsu, P. D.; Habib, N.; Gootenberg, J. S.; Nishimasu, H.; Nureki, O.; Zhang, F. Genome-Scale Transcriptional Activation by an Engineered CRISPR-Cas9 Complex. *Nat. 2014 5177536* **2014**, *517* (7536), 583–588. https://doi.org/10.1038/nature14136.

(5) Liu, S. J.; Horlbeck, M. A.; Cho, S. W.; Birk, H. S.; Malatesta, M.; He, D.; Attenello, F. J.; Villalta, J. E.; Cho, M. Y.; Chen, Y.; Mandegar, M. A.; Olvera, M. P.; Gilbert, L. A.; Conklin, B. R.; Chang, H. Y.; Weissman, J. S.; Lim, D. A. CRISPRi-Based Genome-Scale Identification of Functional Long Non-Coding RNA Loci in Human Cells. *Science* **2017**, *355* (6320). https://doi.org/10.1126/SCIENCE.AAH7111.

(6) Liu, S. J.; Horlbeck, M. A.; Cho, S. W.; Birk, H. S.; Malatesta, M.; He, D.; Attenello, F. J.; Villalta, J. E.; Cho, M. Y.; Chen, Y.; Mandegar, M. A.; Olvera, M. P.; Gilbert, L. A.; Conklin, B. R.; Chang, H. Y.; Weissman, J. S.; Lim, D. A. CRISPRi-Based Genome-Scale Identification of Functional Long Non-Coding RNA Loci in Human Cells HHS Public Access LncRNA Knockdown Can Perturb Complex Transcriptional Networks in a Cell Type-Specific Manner. These Data Underscore the Functional Importance. *Sci. January* **2017**, *06* (3556320), 1–19. https://doi.org/10.1126/science.aah7111.CRISPRi-based.

(7) Shalem, O.; Sanjana, N. E.; Hartenian, E.; Shi, X.; Scott, D. A.; Mikkelsen, T. S.; Heckl, D.; Ebert, B. L.; Root, D. E.; Doench, J. G.; Zhang, F. Genome-Scale CRISPR-Cas9 Knockout Screening in Human Cells. *Science (80-.).* **2014**, *343* (6166), 84–87. https://doi.org/10.1126/SCIENCE.1247005.

(8) Meitinger, F.; Ohta, M.; Lee, K. Y.; Watanabe, S.; Davis, R. L.; Anzola, J. V.; Kabeche, R.; Jenkins, D. A.; Shiau, A. K.; Desai, A.; Oegema, K. TRIM37 Controls Cancer-Specific Vulnerability to PLK4 Inhibition. *Nature* **2020**, *585* (7825), 440–446. https://doi.org/10.1038/s41586-020-2710-1.

(9) Belk, J. A.; Yao, W.; Ly, N.; Freitas, K. A.; Chen, Y. T.; Shi, Q.; Valencia, A. M.; Shifrut, E.; Kale, N.; Yost, K. E.; Duffy, C. V.; Daniel, B.; Hwee, M. A.; Miao, Z.; Ashworth, A.; Mackall, C. L.; Marson, A.; Carnevale, J.; Vardhana, S. A.; Satpathy, A. T. Genome-Wide CRISPR Screens of T Cell Exhaustion Identify Chromatin Remodeling Factors That Limit T Cell Persistence. *Cancer Cell* **2022**, *40* (7), 768-786.e7. https://doi.org/10.1016/j.ccell.2022.06.001.

(10) Huang, H.; Zhou, P.; Wei, J.; Long, L.; Shi, H.; Dhungana, Y.; Chapman, N. M.; Fu, G.; Saravia, J.; Raynor, J. L.; Liu, S.; Palacios, G.; Wang, Y. D.; Qian, C.; Yu, J.; Chi, H. In Vivo CRISPR Screening Reveals Nutrient Signaling Processes Underpinning CD8+ T Cell Fate Decisions. *Cell* **2021**, *184* (5), 1245-1261.e21. https://doi.org/10.1016/j.cell.2021.02.021.

(11) Frangoul, H.; Altshuler, D.; Cappellini, M. D.; Chen, Y.-S.; Domm, J.; Eustace, B. K.; Foell, J.; de la Fuente, J.; Grupp, S.; Handgretinger, R.; Ho, T. W.; Kattamis, A.; Kernytsky, A.; Lekstrom-Himes, J.; Li, A. M.; Locatelli, F.; Mapara, M. Y.; de Montalembert, M.; Rondelli, D.; Sharma, A.; Sheth, S.; Soni, S.; Steinberg, M. H.; Wall, D.; Yen, A.; Corbacioglu, S. CRISPR-Cas9 Gene Editing for Sickle Cell Disease and β-Thalassemia. *N. Engl. J. Med.* **2021**, *384* (3), 252–260. https://doi.org/10.1056/nejmoa2031054.

(12) Maeder, M. L.; Stefanidakis, M.; Wilson, C. J.; Baral, R.; Barrera, L. A.; Bounoutas, G. S.; Bumcrot, D.; Chao, H.; Ciulla, D. M.; DaSilva,

J. A.; Dass, A.; Dhanapal, V.; Fennell, T. J.; Friedland, A. E.; Giannoukos, G.; Gloskowski, S. W.; Glucksmann, A.; Gotta, G. M.; Jayaram, H.; Haskett, S. J.; Hopkins, B.; Horng, J. E.; Joshi, S.; Marco, E.; Mepani, R.; Reyon, D.; Ta, T.; Tabbaa, D. G.; Samuelsson, S. J.; Shen, S.; Skor, M. N.; Stetkiewicz, P.; Wang, T.; Yudkoff, C.; Myer, V. E.; Albright, C. F.; Jiang, H. Development of a Gene-Editing Approach to Restore Vision Loss in Leber Congenital Amaurosis Type 10. *Nat. Med.* **2019**, *25* (2), 229–233. https://doi.org/10.1038/s41591-018-0327-9.

(13) Stadtmauer, E. A.; Fraietta, J. A.; Davis, M. M.; Cohen, A. D.; Weber, K. L.; Lancaster, E.; Mangan, P. A.; Kulikovskaya, I.; Gupta, M.; Chen, F.; Tian, L.; Gonzalez, V. E.; Xu, J.; Jung, I. young; Joseph Melenhorst, J.; Plesa, G.; Shea, J.; Matlawski, T.; Cervini, A.; Gaymon, A. L.; Desjardins, S.; Lamontagne, A.; Salas-Mckee, J.; Fesnak, A.; Siegel, D. L.; Levine, B. L.; Jadlowsky, J. K.; Young, R. M.; Chew, A.; Hwang, W. T.; Hexner, E. O.; Carreno, B. M.; Nobles, C. L.; Bushman, F. D.; Parker, K. R.; Qi, Y.; Satpathy, A. T.; Chang, H. Y.; Zhao, Y.; Lacey, S. F.; June, C. H. CRISPR-Engineered T Cells in Patients with Refractory Cancer. *Science (80-.).* **2020**, *367* (6481), 1–20. https://doi.org/10.1126/science.aba7365.

(14) Gillmore, J. D.; Gane, E.; Taubel, J.; Kao, J.; Fontana, M.; Maitland, M. L.; Seitzer, J.; O'Connell, D.; Walsh, K. R.; Wood, K.; Phillips, J.; Xu, Y.; Amaral, A.; Boyd, A. P.; Cehelsky, J. E.; McKee, M. D.; Schiermeier, A.; Harari, O.; Murphy, A.; Kyratsous, C. A.; Zambrowicz, B.; Soltys, R.; Gutstein, D. E.; Leonard, J.; Sepp-Lorenzino, L.; Lebwohl, D. CRISPR-Cas9 In Vivo Gene Editing for Transthyretin Amyloidosis. *N. Engl. J. Med.* **2021**, *385* (6), 493–502. https://doi.org/10.1056/nejmoa2107454.

(15) Qasim, W.; Zhan, H.; Samarasinghe, S.; Adams, S.; Amrolia, P.; Stafford, S.; Butler, K.; Rivat, C.; Wright, G.; Somana, K.; Ghorashian, S.; Pinner, D.; Ahsan, G.; Gilmour, K.; Lucchini, G.; Inglott, S.; Mifsud, W.; Chiesa, R.; Peggs, K. S.; Chan, L.; Farzeneh, F.; Thrasher, A. J.; Vora, A.; Pule, M.; Veys, P. Molecular Remission of Infant B-ALL after Infusion of Universal TALEN Gene-Edited CAR T Cells. *Sci. Transl. Med.* **2017**, *9* (374), 1–8. https://doi.org/10.1126/scitranslmed.aaj2013.

(16) Tebas, P.; Stein, D.; Tang, W. W.; Frank, I.; Wang, S. Q.; Lee, G.; Spratt, S. K.; Surosky, R. T.; Giedlin, M. A.; Nichol, G.; Holmes, M. C.; Gregory, P. D.; Ando, D. G.; Kalos, M.; Collman, R. G.; Binder-Scholl, G.; Plesa, G.; Hwang, W.-T.; Levine, B. L.; June, C. H. Gene Editing of CCR5 in Autologous CD4 T Cells of Persons Infected with HIV. *http://dx.doi.org/10.1056/NEJMoa1300662* **2014**, *370* (10), 901–910. https://doi.org/10.1056/NEJMOA1300662.

(17) Xu, L.; Wang, J.; Liu, Y.; Xie, L.; Su, B.; Mou, D.; Wang, L.; Liu, T.; Wang, X.; Zhang, B.; Zhao, L.; Hu, L.; Ning, H.; Zhang, Y.; Deng, K.; Liu, L.; Lu, X.; Zhang, T.; Xu, J.; Li, C.; Wu, H.; Deng, H.; Chen, H. CRISPR-Edited Stem Cells in a Patient with HIV and Acute Lymphocytic Leukemia. *N. Engl. J. Med.* **2019**, *381* (13), 1240–1247. https://doi.org/10.1056/nejmoa1817426.

(18) Huo, Y.; Aboud, K.; Kang, H.; Cutting, L. E.; Bennett, A. Clinical Endpoints for the Study of Geographic Atrophy Secondary to Age-Related Macular Degeneration. **2017**, *44* (9), 1–13. https://doi.org/10.1097/IAE.0000000000001283.Clinical.

(19) Lu, Y. C.; Parker, L. L.; Lu, T.; Zheng, Z.; Toomey, M. A.; White, D. E.; Yao, X.; Li, Y. F.; Robbins, P. F.; Feldman, S. A.; Van Der Bruggen, P.; Klebanoff, C. A.; Goff, S. L.; Sherry, R. M.; Kammula, U. S.; Yang, J. C.; Rosenberg, S. A. Treatment of Patients with

Metastatic Cancer Using a Major Histocompatibility Complex Class II-Restricted T-Cell Receptor Targeting the Cancer Germline Antigen MAGE-A3. *J. Clin. Oncol.* **2017**, *35* (29), 3322–3329. https://doi.org/10.1200/JCO.2017.74.5463.

(20) Chemello, F.; Chai, A. C.; Li, H.; Rodriguez-Caycedo, C.; Sanchez-Ortiz, E.; Atmanli, A.; Mireault, A. A.; Liu, N.; Bassel-Duby, R.; Olson, E. N. Precise Correction of Duchenne Muscular Dystrophy Exon Deletion Mutations by Base and Prime Editing. *Sci. Adv.* **2021**, *7* (18). https://doi.org/10.1126/sciadv.abg4910.
