# Supplementary material for: Exploring CRISPR-Cas: The transformative impact of gene editing in molecular biology
Source: Mol Ther Nucleic Acids. 2025 Sep 15;36(4):102717. doi: 10.1016/j.omtn.2025.102717 (PMC12506487; doi:10.1016/j.omtn.2025.102717)
Supplement: Document S2. Article plus Supplemental information [file mmc2.pdf]

Molecular Therapy
Nucleic Acids
Review

Exploring CRISPR-Cas: The transformative impact of gene editing in molecular biology

Vivek Pandey,[1,2,3] Shivani Sharma,[1,3] and Yuba Raj Pokharel[1]

[1]Faculty of Life Sciences and Biotechnology, South Asian University, New Delhi 110068, India; [2]Dr. B.R. Ambedkar Centre for Biomedical Research, University of Delhi, New Delhi 110007, India

This review traces the evolution of clustered regularly interspaced short palindromic repeats (CRISPR) technology from a prokaryotic immune mechanism to a versatile tool for precise genome engineering. We compare CRISPR with traditional gene-editing methods like RNA interference (RNAi), zinc finger nucleases (ZFNs), and transcription activator-like effector nucleases (TALENs), emphasizing its advantages in target specificity, multiplexing, and ease of design. We examine various Cas enzyme classes, engineered variants, and their applications in dissecting genetic alterations at the cellular level. The review further explores CRISPR's expanding role in developing disease models using tissues, organoids, and animal systems, enhancing our understanding of disease mechanisms. Finally, we discuss CRISPR's emerging applications in diagnostics and its transformative impact on immunotherapy and cell-based cancer treatments.

INTRODUCTION

Over the past century, the global disease burden on account of non-communicable diseases has been increasing and affecting the productivity of millions. The toll of death on account of non-communicable diseases is also rising continuously. There is a shift in the burden of disease from the previous century, dominated by the widespread prevalence of communicable diseases, to that of non-communicable diseases.[1] This shift led to the development of antimicrobial drugs, including antibiotics, antivirals, and antifungals. However, contrary to communicable diseases, the number of people affected by non-communicable diseases is gradually increasing, and this could be due to the change in lifestyle practices, increasing pollution of natural resources, and rising concentration of toxic elements in food products. This has led to an increase in the number of people suffering from these diseases. Today, cardiovascular diseases are the leading cause of death, followed by different kinds of cancers.[1] According to the latest estimates from the World Health Organization (WHO) and the International Agency for Research on Cancer, approximately 20 million new cancer cases and 9.7 million cancer-related deaths occurred worldwide in 2022. Lung cancer remained the leading cause of cancer mortality, accounting for about 1.8 million deaths (18.7%), followed by colorectal cancer with 900,000 deaths (9.3%), liver cancer with 760,000 deaths (7.8%), breast cancer with 670,000 deaths (6.9%), and stomach cancer with 660,000 deaths (6.8%).[2] These statistics highlight the persistent global burden of cancer and emphasize the critical need for improved strategies in early diagnosis, prevention, and treatment.

Cancer encompasses a diverse group of diseases characterized by distinct molecular signatures and clinical presentations. Traditional cancer classifications include carcinomas (epithelial origin), sarcomas (mesenchymal origin), leukemias and lymphomas (hematopoietic origin), and central nervous system tumors.[3] Recent advances in molecular profiling have led to the identification of novel cancer subtypes and rare malignancies. Emerging cancers of clinical significance include primary effusion lymphoma associated with Kaposi sarcoma-associated herpes virus (KSHV) infection, anaplastic lymphoma kinase-positive large B cell lymphoma, and various fusion-driven sarcomas such as Capicua Transcriptional Repressor (CIC)-rearranged sarcomas.[4] Additionally, the COVID-19 pandemic has revealed potential links between SARS-CoV-2 infection and certain haematological malignancies, though causality remains under investigation.[5] Environmental carcinogens continue to drive the emergence of new cancer types, including e-cigarette or vaping product use-associated lung injury-related malignancies and occupational cancers linked to novel industrial chemicals.[6] Furthermore, advances in immunodeficiency treatments have unmasked previously rare cancers such as KSHV-related disorders and Epstein-Barr virus (EBV)-positive mucocutaneous ulcers in immunocompromised patients.[7]

WHO defines cancer as "a disease where the abnormal cells grow uncontrollably and move beyond the restricted cellular limits and invade healthy cells." These cells have defects in the regulatory circuit that control proliferation and homeostasis. This aberration in the circuit is attributed to a myriad of factors, including genetic, epigenetic, environmental, and behavioral.[8] Apart from genetics, all other causes have some other triggering external agents. These include physical agents such as UV rays and ionizing radiation; chemical stimuli such as cadmium, lead, and asbestos; and biological agents like bacteria and viruses, in particular Human Papillomavirus (HPV), Hepatitis B Virus (HBV), and EBV.[9]

https://doi.org/10.1016/j.omtn.2025.102717.

[3]These authors contributed equally

Correspondence: Yuba Raj Pokharel, Faculty of Life Sciences and Biotechnology, South Asian University, New Delhi 110068, India.
E-mail: yrp@sau.ac.in

© 2025 The Author(s). Published by Elsevier Inc. on behalf of The American Society of Gene and Cell Therapy. This is an open access article under the CC BY license (http://creativecommons.org/licenses/by/4.0/).

Table 1. Comparative analysis of genome editing tools: RNAi, ZFNs, TALENs, and CRISPR-Cas systems

Sr. No.	Parameter	ZFN	TALEN	CRISPR-Cas
1	efficiency	0%–12%, low	0%–76%, moderate	0%–81%, high
2	interacting partners	protein-DNA	protein-DNA	DNA-RNA
3	possible target site	18–36 bp/ZFN pair	30–40 bp/TALEN pair	22 bp
4	off-target effect	less predictable	less predictable	highly predictable
5	ease of designing	difficult, two ZFNs around the target sequence	difficult, two TALENs around the target sequence	easy, sgRNA complementary to the target sequence with Cas protein
6	multiplexing	less feasible	less feasible	highly feasible (no need of ESCs)
7	large-scale library construction and genome-wide screening	challenging (need individual gene tailoring)	challenging (need individual gene tailoring)	easy (only requires plasmid containing small oligonucleotides)
8	affordability	low	low	high
9	immune response	low	low	high
10	*in vivo* delivery system	AAV	AAV	AAV, lentivirus

The table highlights differences in the mechanism of action, target design complexity, efficiency, specificity, multiplexing potential, and therapeutic applicability.

Traditionally, the cancer therapies were limited to surgery, chemotherapy, and radiotherapy, or their combinations.[10] However, these therapies had a hidden disadvantage of non-selectivity, resulting in the death of healthy cells of the patients. With the advent of modern biological sciences and discoveries in the fields of molecular biology, protein engineering, and nano-medicines, numerous more selective approaches have been developed.[11,12] The development of genome editing tools based on bacterial and engineered nucleases has made it possible to study the effect of even a single gene and knock it out in case of any defect. These techniques are based on the removal of specific sequences of DNA using nucleases, producing double-strand breaks (DSBs).[13] These DSBs are then repaired by either the non-homologous end joining (NHEJ) pathway or the homology-directed repair (HDR) pathway, resulting in gene disruption or replacement with the targeted sequence.[14,15] Zinc finger nuclease (ZFN) and transcription activator-like effector nucleases (TALENs) were then the pioneer tools in this direction.[16,17] Both of them are based on protein-DNA interactions for the recognition of the target site. Thus, even though they eliminated the non-specificity and experimental variation associated with RNA interference (RNAi), they became limited by their design. For every new gene, the amino acid sequence needs to be changed, making the process cumbersome. Moreover, the high molecular weight of the protein also created a problem with multiplexing and loading it into the carrier vector.[18] The discovery of clustered regularly interspaced short palindromic repeats (CRISPR) and CRISPR-associated proteins (Cas) as a bacterial defense mechanism and its development as a genome editing tool completely revolutionized the field. Unlike its predecessors, it does not rely on protein-DNA interactions but is based on DNA-RNA recognition for targeting the specific site. A single nuclease can be used for all genes under study, just by changing the sequence of the guide RNA (gRNA).[19] A comparative analysis of the three genome editing tools is presented in Table 1.

Advancements in CRISPR technology have revolutionized the study of genes by enabling precise regulation of gene expression, high-throughput genome screening, and efficient multiplexed editing with reduced off-target effects. Beyond its transformative role in genetic studies, CRISPR has emerged as a valuable tool in epigenetic research, allowing for targeted modulation of chromatin states and transcriptional activity without altering the underlying DNA sequence. These breakthroughs have laid the foundation for the development of precision medicine, a paradigm that leverages individual genetic profiles to guide personalized therapeutic interventions.

This review traces the journey of the CRISPR system from its origins as an adaptive immune mechanism in prokaryotes to its current status as a versatile genome engineering platform. We detail the evolution and engineering of various Cas protein variants, their functional adaptations, and how these innovations have expanded the scope of CRISPR applications. Particular emphasis is placed on the utility of CRISPR in cancer genomics, where it has accelerated the functional interrogation of oncogenes and tumor suppressors, enabled the development of more accurate cancer models, and facilitated drug target discovery. Finally, we explore the growing role of CRISPR-based technologies in cancer diagnostics and their integration into ongoing clinical trials, underscoring their potential to transform the landscape of cancer treatment.

CRISPR-Cas9 origin and working mechanism

The term CRISPR was coined first in 2002 by Jansen et al., though it was first reported in 1987 by Ishino et al. in *Escherichia coli.*[20,21] It is a part of the adaptive immunity component based on nucleic acid recognition, followed by cleavage in bacteria and archaea.[22] It targets the offending agents, such as bacteriophages and harmful foreign DNA, by inducing RNA-guided DNA cleavage.[23–25] The CRISPR system has three functional components: Cas, which serves as the endonuclease; a CRISPR-targeting RNA (crRNA); and a *trans*-activating

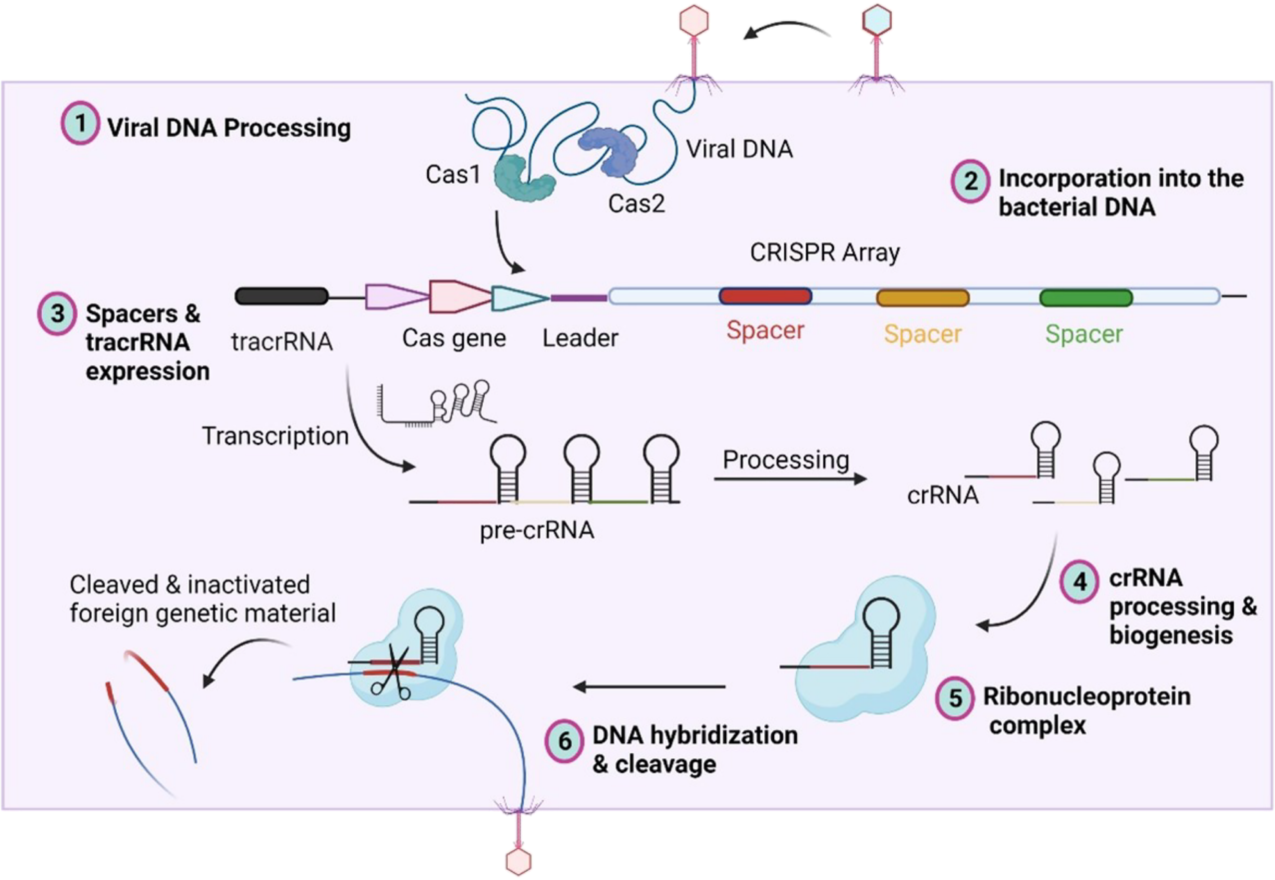

Figure 1. Mechanism of CRISPR-Cas9 gene-editing tool

Steps 1 and 2 (adaptation phase): viral or plasmid DNA would be processed into protospacers and integrated into repeat sequences to form the CRISPR array through Cas1 and Cas2. Specific CRISPR locus (from *Streptococcus pyogenes*) consists of tracrRNA sequence, several Cas genes, leader sequence, and CRISPR array. Steps 3 and 4 (crRNA biogenesis): CRISPR array transcribes into pre-crRNA. The tracrRNA combines pre-crRNA to form a mature tracrRNA-crRNA complex processed by nucleases. Steps 5 and 6 (interference phase): this complex activates Cas9 endonuclease and recognizes a 20-nt crRNA complementary sequence within the exogenous gene, while Cas9 finds PAMs. The double-stranded DNA would be cleaved at 3 nt upstream of the PAMs ultimately by Cas9 endonuclease. CRISPR, clustered regularly interspaced short palindromic repeats; tracrRNA, *trans*-activating CRISPR RNA; crRNA, CRISPR RNA; PAMs, protospacer adjacent motifs.

RNA (tracrRNA), where the latter guides the Cas protein to the target site recognized by the crRNA for cleavage (Figure 1).[26] It is a three-step process that starts with spacer acquisition, then biogenesis of crRNA, and ends with target interference of foreign DNA, thereby conferring resistance to bacteria against the targeted virus or plasmid.[27] The acquisition/adaptation phase involves splicing the invasive DNA into small fragments, followed by their incorporation into the CRISPR locus.[28] This incorporation happens upstream of the existing spacers adjacent to the leader sequence. The leader sequence is a 500 bp AT-rich region having a binding site for the promoter required in crRNA transcription.[24] The incorporated spacer has sequence homology to the invading DNA and a unique 2–3 nucleotide motif, known as the protospacer adjacent motif (PAM), that is unique to each CRISPR-Cas type. The PAM motif, which is NGG ("N" represents any nucleotide A, T, C, or G, and "GG" represents two guanine nucleotides) in the case of Cas9, is essential for recognition and cleavage, as sequences lacking it are excluded.[29]

The fundamental difference between T-rich and G-rich PAM requirements has significant implications for genome editing applications and target accessibility. G-rich PAMs, such as the NGG sequence recognized by SpCas9, are distributed throughout the genome with an average occurrence of every 8–12 base pairs in human DNA. However, this distribution is not uniform across all genomic regions. GC-rich regions, including many gene promoters and cytosine-phosphate-guanine (CpG) islands, contain abundant NGG sites, while AT-rich regions such as heterochromatin and certain intergenic sequences have limited Cas9 accessibility.[30] In contrast, T-rich PAMs like the TTTV sequence recognized by Cas12a provide complementary targeting capabilities. These PAMs are more frequently found in AT-rich genomic regions, including regulatory elements, enhancers, and gene bodies that are often inaccessible to Cas9. This expanded targeting range is particularly valuable for editing genes in heterochromatic regions and for targeting specific cell types where AT-rich sequences predominate.[31]

The biochemical basis for these PAM preferences lies in the distinct protein-DNA interaction mechanisms. Cas9 recognizes G-rich PAMs through specific amino acid-nucleotide contacts in its PAM-interacting domain, with key residues R1333 and R1335 specifically contacting the guanine bases through hydrogen bonding interactions. In contrast, Cas12a employs a different set of residues, including K946, K951, and R947 that are optimized for T-rich sequence recognition through distinct hydrogen bonding patterns with thymine bases.[32] This fundamental difference in molecular recognition allows researchers to select the most appropriate nuclease based on the genomic context of their target site.

Practical implications include improved editing efficiency in previously challenging genomic regions. For instance, targeting tumor suppressor genes located in AT-rich regions benefits from Cas12a's T-rich PAM requirement, while oncogenes in GC-rich promoter regions remain accessible to Cas9. This complementary targeting capability enhances the overall utility of CRISPR systems in cancer research and therapeutic applications.

Biogenesis happens after the incorporation of the spacer sequence into the CRISPR array. It involves the production of precursor CRISPR-RNA (pre-crRNA) by transcription. The pre-crRNA is cleaved into mature crRNA by a complex having RNA, tracrRNA, RNase III, and Cas9.[33,34] The tracrRNA has a sequence complementary to the repeat sequence of the pre-crRNA and an additional sequence resulting in the formation of three stem-loops, supportive of nuclease recruitment.[35] Though significantly important in most types of Cas, they are not required in type V and some type VI variants, i.e., in Cas 12 and Cas 13.[36]

Interference concludes the last phase of the defense mechanism, where a trihybrid complex containing crRNA-tracrRNA-Cas recognizes the foreign DNA complementary to the crRNA by base pairing and cleaves it via its endonuclease activity.[37] This endonuclease activity generates DSBs due to the presence of the bilobed cleavage domain in Cas9. The two conserved domains, RuvC (named after RuvulaC resolvase) and histidine-asparagine-histidine motif (HNH), get activated upon recognition of complementary base-pairing between crRNA and the foreign DNA, resulting in cleavage of the foreign DNA and generating DSBs.[38] The generation of DSBs results in the activation of the DNA repair mechanism via two pathways, i.e., NHEJ and HDR. The former results in creating indels, whereas the latter results in the replacement of the mutated/wild-type sequence with the sequence having the desired characteristics (Figure 2).[39] The generation of DSBs has significant therapeutic implications in cancer treatment, as different cancer types exhibit varying sensitivities to DNA damage based on their repair pathway deficiencies. Breast cancer susceptibility genes (BRCA)-deficient breast and ovarian cancers show enhanced vulnerability to CRISPR-induced DSBs due to impaired homologous recombination, creating synthetic lethality opportunities.[40] Similarly, microsatellite instability-high colorectal cancers demonstrate increased sensitivity to DSB-inducing treatments due to defective mismatch repair systems.[41] Recently characterized cancer subtypes, including chromophobe renal cell carcinoma and adenoid cystic carcinomas with specific fusion proteins, have revealed novel DSB-related therapeutic vulnerabilities.[42,43] In glioblastoma, CRISPR-mediated targeting of DNA repair genes like MGMT in combination with alkylating agents has shown enhanced efficacy in clinical trials.[44]

In 2012, Jinek et al. catalyzed a major breakthrough in CRISPR engineering by designing a chimeric RNA molecule that simplified and streamlined the system's functionality. This innovation involved fusing the 3′ end of the crRNA with the 5′ end of the tracrRNA, resulting in a single-guide RNA (sgRNA).[37] This engineered sgRNA retained the dual functionality of recognizing the target DNA through its spacer sequence and recruiting the Cas9 endonuclease via the tracrRNA scaffold. The team successfully generated five such sgRNAs, each capable of effectively silencing green fluorescent protein expression *in vitro*, thus validating the approach.[32] The therapeutic efficacy of CRISPR-based cancer interventions fundamentally depends on sgRNA design parameters that differ substantially from research applications. Cancer cells present unique challenges, including genomic instability, heterogeneous mutations, and altered chromatin landscapes that influence sgRNA accessibility and binding kinetics. Optimal sgRNA selection for cancer targets requires a comprehensive analysis of tumor-specific mutation patterns, as single-nucleotide variations can dramatically affect sgRNA efficiency and specificity.[45] Clinical translation has revealed that sgRNA performance varies significantly between cancer subtypes, necessitating personalized guide design strategies. For instance, sgRNAs targeting mutant p53 variants show differential activity across various cancer types due to distinct chromatin contexts and co-occurring mutations.[46] Contemporary approaches incorporate machine learning algorithms to predict sgRNA efficacy in cancer-specific contexts, accounting for factors such as DNA methylation patterns, histone modifications, and transcriptional activity levels.[47] Additionally, multiplexed sgRNA strategies enable simultaneous disruption of cooperating oncogenic pathways, addressing the polygenic nature of cancer while minimizing treatment resistance mechanisms.[48] In recent times, CRISPR-Cas9 has emerged as a robust, versatile, and user-friendly genome editing tool, with applications rapidly expanding across virtually all domains of life.

Development and diversification of CRISPR-Cas systems

Class 1 CRISPR-Cas systems (types I, III, and IV) use multi-protein effector complexes that require multiple Cas proteins working together to recognize and cleave target sequences.[49] Type I systems use Cas3 as the signature effector nuclease. These systems recognize DNA as their target and induce single-strand breaks, often in a processive manner. This system has been studied for its role in bacterial immunity, but it is less developed for genome editing due to its complexity.[50]

Type III systems are unique in that they target nascent RNA transcripts, with Cas10 functioning as the key effector molecule, often displaying both RNase and DNase activities depending on the

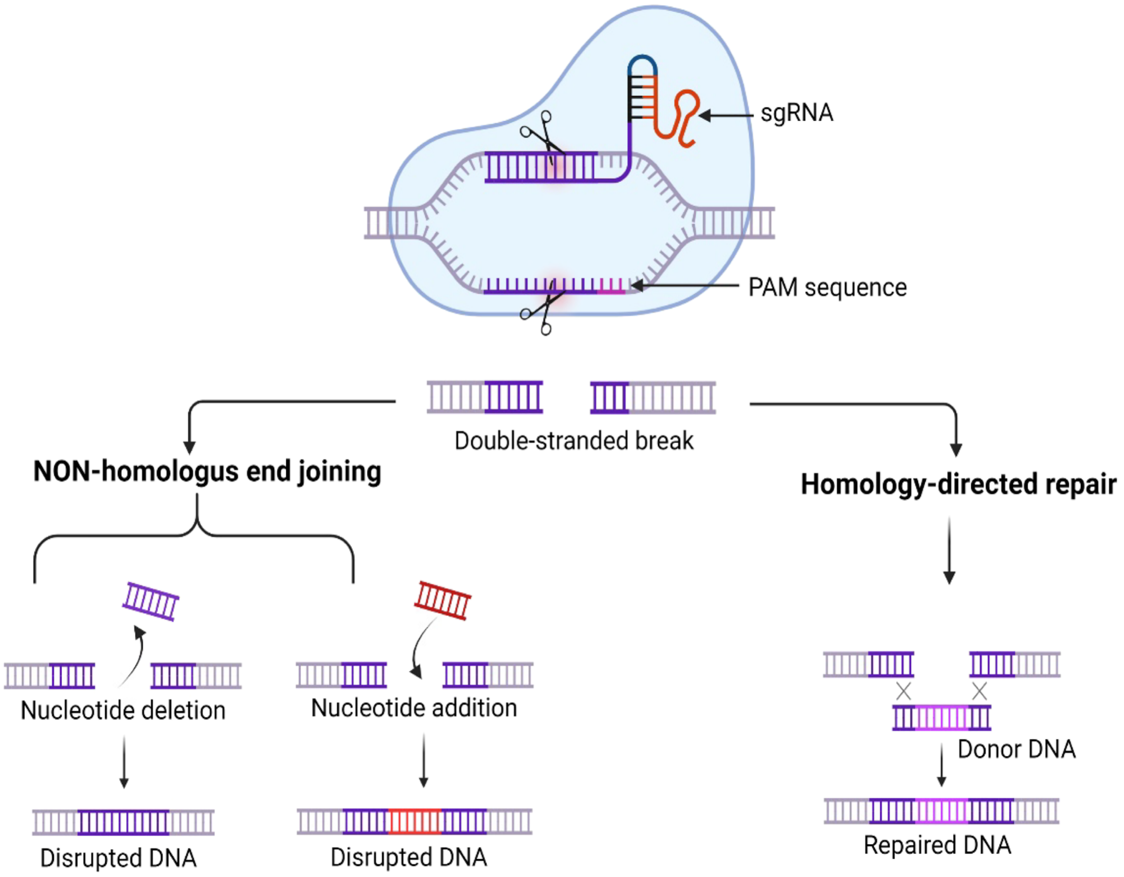

Figure 2. CRISPR-Cas9-mediated genome editing system
The chimeric sgRNA recruits Cas9 to the target DNA. It contains a protospacer followed by PAM to recognize the target sequence. Cas9-induced DSBs are repaired either by NHEJ giving rise to indel mutations or by HDR using a synthetic donor DNA template, enabling the introduction of desired sequence changes. sgRNA, single-guide RNA; PAM, protospacer adjacent motif; DSBs, double-strand breaks; NHEJ, non-homologous end joining; HDR, homology-directed repair.

context of the infection. However, the dual cleavage activity complicates its use in precision genome editing.[51]

Type IV systems remain poorly characterized. They typically encode CRISPR-associated sequence factor 1 as the putative signature protein, but their exact biological roles and mechanisms are still under investigation.[52]

In contrast, class 2 CRISPR-Cas systems (types II, V, and VI) are characterized by a single, multidomain effector protein capable of performing all necessary functions for interference. This structural simplicity has made class 2 systems more amenable to adaptation for genome engineering purposes.[53,54]

Type II systems utilize Cas9, the most well-known and widely applied CRISPR effector, which binds to dsDNA and introduces blunt-ended DSBs.[55]

Type V systems, represented by Cas12, also target DNA but differ mechanistically by generating staggered DSBs with overhangs, and some subtypes possess collateral cleavage activity.[56] Cas12a recognizes a T-rich PAM (e.g., TTTV), expanding the range of editable sequences compared to SpCas9, which requires a G-rich PAM. Unlike SpCas9, Cas12a generates staggered cuts, creating “sticky ends” that are beneficial for specific genetic modifications. Additionally, Cas12a can autonomously process its crRNA array, enabling more efficient multiplexed genome editing without the need for additional tracrRNA sequences.[56]

Type VI systems employ Cas13, which is unique in its ability to bind and cleave single-stranded RNA, exhibiting both specific and collateral RNase activity.[57] Upon binding to its target RNA, Cas13a displays a distinctive collateral cleavage activity, which leads to the indiscriminate degradation of nearby non-target RNAs.[58]

Due to their streamlined architecture and remarkable functional versatility, class 2 CRISPR systems, particularly Cas9, Cas12, and Cas13, have become foundational tools in modern genome engineering. These systems, characterized by their single-protein structure, offer distinct advantages over the more complex class 1 systems,

such as simplified programming, efficiency, and precision, making them ideal candidates for diverse applications in molecular biology.

Cas9

The initial discovery in CRISPR biotechnology was primarily based on Cas9 as the effector endonuclease. Cas9 is a large, multidomain protein comprising approximately 1,368 amino acids with a molecular weight of ~160 kDa. Its crystal structure reveals a bilobed architecture consisting of a recognition (REC) lobe and a nuclease (NUC) lobe connected by a flexible bridge helix.[59] The REC lobe contains the arginine-rich bridge helix and REC1 and REC2 domains responsible for sgRNA binding and target DNA recognition. The NUC lobe houses two distinct nuclease domains: the HNH domain, which cleaves the target DNA strand complementary to the gRNA, and the RuvC domain, which cleaves the non-target strand.[60] This dual-nuclease architecture is fundamental to Cas9's ability to generate precise blunt-ended DSBs, distinguishing it from single-nuclease systems. Cas9's catalytic mechanism involves a sophisticated multi-step process beginning with ribonucleoprotein (RNP) complex formation with the sgRNA. Target recognition initiates through PAM scanning, where Cas9 interrogates DNA sequences through rapid binding and dissociation events.[61] Upon encountering the NGG motif, local DNA unwinding occurs, allowing the gRNA spacer to invade and form base pairs with the complementary DNA strand. This R-loop formation triggers critical conformational changes in Cas9, particularly repositioning of the HNH domain to align with the target strand.[62] Only upon successful base-pairing along the entire spacer sequence do both nuclease domains activate simultaneously, ensuring high fidelity targeting. As mentioned earlier, the system produces DSBs, resulting in NHEJ and HDR repair pathways.[63] The enzyme has been a tool of choice for creating indels, resulting in gene disruption and replacing the gene with a target-modified sequence.[64] Traditional Cas9 nucleases rely on the NGG PAM sequence, which limits the range of editable genomic sites, particularly in regions where this motif is sparse or absent. To overcome this limitation, researchers have developed PAM-less or PAM-relaxed Cas9 variants, with *Streptococcus pyogenes* Cas9 relaxed pY motif (SpRY) being one of the most advanced. SpRY was engineered through mutations in its PAM-interacting domain, allowing it to recognize a broader range of PAM sequences, such as NRN and NYN (where "R" denotes a purine and "Y" a pyrimidine).[65] This expanded flexibility enables SpRY to target nearly any genomic site, particularly in AT-rich regions where NGG motifs are less frequent, thereby broadening the possibilities for genome editing.[66] Recent advances demonstrate CRISPR-Cas9's potential for therapeutic chromosome elimination, with allele-specific targeting successfully correcting trisomy 21 by removing excess chromosomes while preserving cellular viability and restoring normal gene expression profiles. This approach represents a paradigm shift from gene-level editing to whole-chromosome manipulation, offering new possibilities for treating aneuploid disorders through precise chromosomal rescue using engineered high-fidelity variants like eSpCas9(1.1), which contains K848A, K1003A, and R1060A mutations for enhanced targeting specificity.[67] Despite its widespread utility, native Cas9 has several limitations, including off-target effects and its strict reliance on NGG PAM sequences, which can limit its editing efficiency in certain genomic regions.[68,69] In addition to the mutation and gene editing functions, it has also been modified to perform inhibition and activation of the gene functions as CRISPR interference (CRISPRi) and CRISPR activation (CRISPRa) via dead Cas9 (dCas9).[70] Further, an alternative to DSBs that introduce non-targeted indels, Cas9 nickase (Cas9n), could be used to introduce specific single-nucleotide modifications.

dCas9

The dCas9 is a modified version of Cas9, containing a mutationally inactivated RuvC and HNH domain responsible for the nucleolytic activity of Cas9.[59] The inactivation is introduced through two-point mutations, D10A and H840A, inactivating RuvC and HNH domains, respectively. dCas9 with lost catalytic activity is still functional in performing the site recognition function.[71] A mutant protein with lost endonuclease activity but intact recognition function causes the repression of transcription by sterically inhibiting the RNA polymerase (RNAP) progression at the site of gRNA recognition. This can be used to study initiation and elongation events based on the targeted region. The aforementioned repression can be rescued by fusing the ω subunit of the RNAPII with the enzyme. In addition, dCas9 has enlarged the scope of CRISPR technology beyond nuclease activity. dCas9 can be reprogrammed to study epigenomic modeling factors and chromatin structures and perform base-editing functions as well.[72] Targeted DNA methylation or demethylation can be achieved by fusing dCas9 with enzymes like DNA methyltransferase 3A (DNMT3A) or ten-eleven translocation 1 (TET1), respectively. Such modifications have been applied to regulate gene expression in various cell types, including cancer cells.[73] Chimeric dCas9 fused with different proteins of choice can be used as an activator or repressor to carry out a genome-wide functionalization assay. The repressor modification is called CRISPRi, and the activator is called CRISPRa.[74] Further, modifications in the dCas9 are used to perform epigenomic studies and protein interaction studies (Figure 3).

CRISPRi

dCas9-mediated transcriptional silencing of the target gene upon binding is referred to as CRISPRi. The chimeric molecule of dCas9 is fused with repressors, resulting in gene inactivation upon binding to the target site mediated by gRNA recognition. Interference approaches target promoter-proximal and enhancer regions lying between −50 and +300 of the gene start site.[75] Kruppel-associated box (KRAB) and enhancer of Zest homology 2 (EZH2) are prominent transcriptional repressor domains utilized in CRISPRi approaches for targeted gene silencing.[76] KRAB, found in approximately one-third of all mammalian zinc finger proteins, functions as a powerful transcriptional repressor when fused to dCas9.[77,78] The KRAB domain serves as a scaffold that recruits KRAB-associated protein 1, also known as TRIM28, which subsequently establishes repressive chromatin modifications, including trimethylation of lysine 9 on histone H3 (H3K9, cme3) and local DNA methylation.[79–81] This recruitment leads to heterochromatin formation

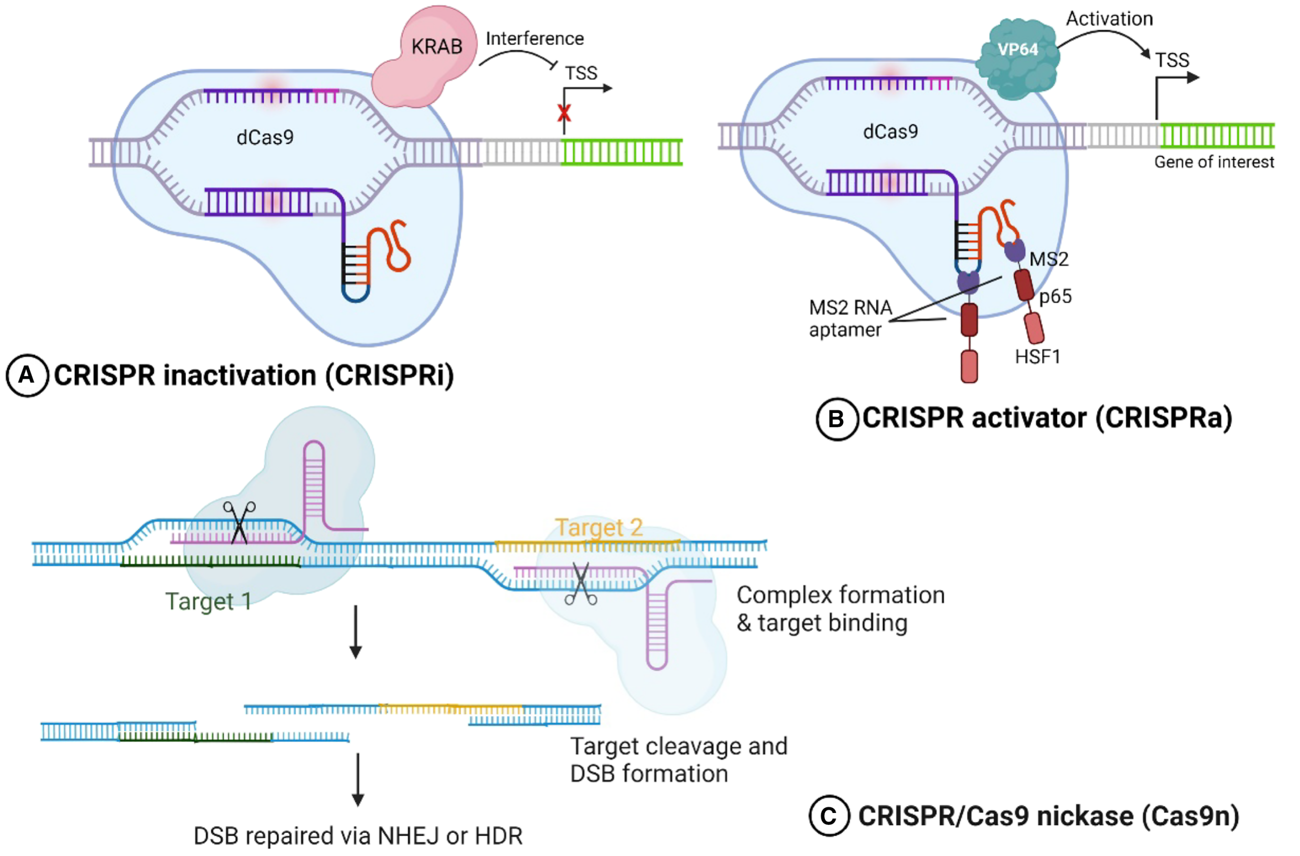

Figure 3. CRISPR-Cas9 tools for genetic engineering

(A) CRISPRi-mediated transcription initiation repression via pairing repressor domain KRAB with dCas9 to block RNA polymerase and, hence, gene expression. (B) CRISPRa-mediated gene overexpression via direct fusion of dCas9 with a transcriptional activator domain VP64 and modified sgRNA stem-loops that recruit transcriptional factors (VP64 and MS2-p65-HSF1). (C) Cas9n: the specificity of CRISPR-Cas9 can be improved by using paired Cas9n to introduce DNA breaks, which have reduced affinity for the DNA backbone. CRISPRi, CRISPR interference; KRAB, Kruppel-associated box; dCas9, dead Cas9; CRISPRa, CRISPR activation; sgRNA, single-guide RNA; VP64, viral protein 64; MS2, MS2 coat protein; p65, transcriptional activator p65; HSF1, heat shock factor 1; Cas9n, Cas9 nickase.

and can silence genes up to 10 kb from the target site through long-range chromatin compaction.

EZH2, the catalytic subunit of polycomb repressive complex 2, represents an alternative repressor strategy that functions through histone methylation. When fused to dCas9, EZH2 catalyzes trimethylation of lysine 27 on histone H3 (H3K27me3), a key epigenetic mark associated with facultative heterochromatin and gene silencing.[76] Studies have demonstrated that EZH2-dCas9 can achieve locus-specific long-term gene repression when combined with DNA methyltransferases (DNMT3A-dCas9 + DNMT3L), particularly effective for genes with active chromatin marks.[76,80] The former includes a dual system, dCas9-KRAB and dCas9-DNMT3A targeting the same region, while the latter includes the dCas9-KRAB-methyl-CpG binding protein 2 (MeCP2) complex. Further, dCas9-SunTag-DNMT3A recruits multiple antibody-tagged DNMT3A to the SunTag, resulting in silencing.[76,82] The choice between KRAB and EZH2 repressor domains appears to be context dependent, with different loci showing varying sensitivities to each approach. Recent *in vivo* studies have confirmed that epigenetic editors containing these repressor domains can achieve durable gene silencing lasting nearly 1 year in mice, with silencing maintained even after forced tissue regeneration.[76]

CRISPRa

Transcriptional activation of the target gene in a dCas9-dependent manner is referred to as CRISPRa. It generally targets the promoter proximal region from −300 to +50 of the gene start site by fusing dCas9 with a potent transcription activator protein.[83] These include multimers of VP16, p300, p65, a subunit of nuclear factor kappa-light-chain-enhancer of activated B cells, or p65 fusion with heat shock protein (p65-HSF1).[84,85] These proteins are used due to their transactivating potential at the promoter start site. The activation strength can be further modified by modifying Cas, gRNA, or both. In the Cas modification system, an array of general control nonderepressible 4 peptides is fused to the dCas9, resulting in what is called SunTag, to which small-chain variable fragment (scFv)-fused activation domains p65 and p300 bind.[86,87] The guide

modification approaches include the addition of aptamer sequences to the gRNA, which recruits the aptamer-binding protein fused with the transcription activation domain. The tripartite recruitment enhancement element system, which can recruit up to 32 molecules, combines both approaches.[88]

Cas9n

Cas 9 nickase (Cas9n) is a modified version of Cas9 created by introducing the point mutation D10A in the RuvC nucleolytic domain of the Cas9 protein and leaving only one functional nuclease domain. The resulting Cas9n generates single-strand breaks called nicks instead of creating DSBs.[89] These single-stranded breaks get repaired by the base excision mechanism, which is much more precise than the NHEJ following DSBs.[32,90] Thus, Cas9n serves as an effective tool in altering/restoring gene function by creating point mutations. Paired nickases, which target opposite DNA strands individually, improve editing precision by generating DSB-like effects while significantly reducing off-target activity. This strategy offers a more accurate and safer approach to genome editing.[91]

Prime editors are composed of Cas9n fused to a reverse transcriptase (RT) and use a specialized prime editing guide RNA (pegRNA) that encodes both the target site and the desired edit to be made, thus allowing conversion of all 12 target bases in the sequence, as well as insertions and deletions. Similar to base editing, it also does not create DSBs, and a single nick is sufficient. The nick created at the target site removes the bases that the RT attached to the Cas9n uses pegRNA as the template and incorporates the desired sequence. The cellular repair machinery repairs the mismatch in the opposite strand, thus creating the identical copies. Bulcaen et al. (2024) applied prime editing to correct mutations in the cystic fibrosis transmembrane conductance regulator gene in patient-derived airway epithelial cells, offering a potential therapeutic route for cystic fibrosis.[92] Chen et al. (2021) reported the development of PEmax, an optimized prime editor with higher editing efficiency and improved nuclear localization signals. The system incorporates a mismatch repair inhibitor, which often impedes the prime editing function.[93] Additionally, efforts to expand the targeting scope have included the use of SaCas9 and Cas12b prime editors, allowing for editing in PAM sites beyond NGG.[94]

Base editing modifies DNA at single-nucleotide resolution without cutting both DNA strands. Catalytically impaired Cas9n is fused with a deaminase enzyme, which catalyzes the base changes. In cytosine base editors, a cytidine deaminase (usually rAPOBEC1) converts cytosine (C) to uracil (U), which is then recognized by DNA repair pathways as thymine (T), resulting in a C-G to T-A transition.[95] At times, the presence of uracil in DNA is recognized by the repair machinery; thus, fusion of another protein called uracil DNA glycosylase inhibitor is done, which prevents uracil glycosylase activity. Similarly, in adenine base editors (ABEs), a TadA-derived adenosine deaminase converts adenine (A) to inosine (I), which pairs like guanine (G) during replication, enabling A-T to G-C conversion. Engineered TadA domain in ABEs (e.g., ABE8e) increases the editing in human cells.[96,97] ABE was used to correct a mutation in the PAH gene responsible for phenylketonuria in a mouse model.[98] Yeh et al. (2018) used base editors to target sickle cell disease mutations in human hematopoietic stem cells, restoring normal hemoglobin production with high efficiency.[99] Though highly efficient, they may cause bystander edits where nearby nucleotides within the editing window are unintentionally modified. Modified base editors such as BE4max and ABE8e provide enhanced and improved editing.[99]

Base editors derived from Cas9n achieved clinical success with the Food and Drug Administration (FDA)-approved CTX001 therapy for sickle cell disease and β-thalassemia, which edits the BCL11A gene to reactivate fetal hemoglobin production.[100] The EDIT-101 trial represents the first *in vivo* CRISPR application, directly correcting CEP290 mutations for treating Leber congenital amaurosis.[101] Recently, Musunuru et al.[102] performed base editing with a cytosine base editor to correct the Q335X nonsense mutation in the CPS1 gene, converting a premature stop codon to a functional leucine codon and restoring enzyme function. This groundbreaking application led to KJ Muldoon becoming the first baby to receive personalized CRISPR treatment, marking a historic milestone in precision genetic medicine.[102]

Cas12a (Cpf1)

Cas12a, formerly referred to as CRISPR from *Prevotella* and *Francisella* 1 (Cpf1), is a class 2 CRISPR system. Similar to other members of this class, it also has a single effector endonuclease.[103] However, unlike Cas9, which has HNH and RuvC as nuclease domains, it lacks the former. Along with the RuvC domain, it has an NUC domain that cleaves the target strand only after RuvC cleaves the non-target strand.[36] This results in the production of DSBs; however, unlike Cas9-generated blunt ends, these ends are staggered and have a five-nucleotide 5′ overhang.[104] Staggered breaks produced are repaired preferably following HDR rather than NHEJ, which is less efficient. However, unlike Cas9, which prefers G-rich "NGG" as a PAM motif sequence, Cas12a prefers T-rich "TTN" as the PAM motif for recognition.[105] Cas12a′s T-rich PAM requirement enables targeting of AT-rich regulatory sequences previously inaccessible to Cas9.[106] Cas12a is a unique member of class 2 as it does not require the dual RNA system consisting of crRNA:tracrRNA and requires only crRNA for its functioning.[107] Furthermore, Cas12a possesses intrinsic RNase activity required for cleavage of the precursor RNA and hence does not require host RNase III like Cas9.[108] This makes it more manageable to perform multiplexing of several guides into one sequence so that several characters can be studied simultaneously.[109]

Cas13

Cas13 is another unique member of class 2 of the CRISPR system belonging to type V. The Cas13 system includes three subtypes, Cas13a, Cas13b, and Cas13c.[110] Unlike other members of this class that recognize DNA as a target molecule, it recognizes RNA and causes cleavage. Exclusive RNA cleavage activity is attributed to the presence of a higher eukaryotic and prokaryotic nuclease

Table 2. A comparative analysis of different Cas systems (Cas9, Cas12, and Cas13)

Sr. No.	Parameter	Cas9	Cas12	Cas13
1	RNA	two RNA molecule	single RNA molecule	RNA molecule
2	nuclease site	two nuclease domains, HNH and RuvC	single nuclease domain; NUC	HEPN domains
3	type of cut	blunt-ended dsDNA break	5 nt 5′ overhang dsDNA break	ssRNA
4	size	∼1,000–1,600 aa	∼1,100–1,300 aa	∼900–1,300 aa
5	guide spacer length	18–24 nt	18–25 nt	22–30 nt
6	total guide length	∼100 nt (sgRNA)	42–44 nt	52–66 nt
7	PAM requirements	G rich; 5′-NGG-3′	T rich; 5′-TTN-3′	analog of PAM; protospacer flanking site – 3′ A, U, or C
8	precrRNA processing	requires host RNases III and tracrRNA	possess intrinsic RNase activity	possess intrinsic RNase activity
9	type	2	5	6
10	multiplexing	challenging	possible	possible
11	effector molecule	both crRNA and tracrRNA	only phage crRNA	both crRNA and tracrRNA
12	AAV-mediated delivery	possible	possible	not possible due to the large size
13	viable for *in vivo* system	yes	yes	no
14	availability in the human genome	every ∼8 bp	every ∼23 bp	any location

This table highlights the key differences in target molecules, PAM requirements, cleavage characteristics, and major applications of class 2 CRISPR systems: Cas9, Cas12, and Cas13. The comparison underscores their unique functionalities, making them suitable for a wide range of genome editing and diagnostic purposes.

(HEPN) domain instead of the common RuvC domain, like the other Cas systems.[111] Similar to Cas12a, it too can perform its own pre-crRNA processing that aids in multiplexing, i.e., targeting multiple loci with only one template.[112] Cas13a cleaves the nuclear transcripts, thus providing post-transcriptional repression similar to RNAi techniques, as it targets the specific transcript isoform generated post processing of the pre-mRNA rather than targeting the gene as a whole.[58] Thereby, it avoids the problem of inhibition of alternative isoforms of genes generated through alternative splicing. This is crucial in targeting the diseases generated through mis-splicing the transcripts.[113] Unlike the Cas9-based DNA base editing, which is permanent and irreversible in nature, Cas13b has been modified to perform RNA base edits that are transient and reversible, as changes happen at the transcript level, reducing the risk of permanent off-target effects. It uses RNA editing for a programmable A-to-I replacement (REPAIR) system. This involves the fusion of adenosine deaminases acting on RNA (ADAR) domains with Cas13b to perform the reversible RNA base editing.[114] REPAIR mechanism targets A on the target strand with C on the gRNA, responsible for mismatch, and gets converted to I, which is read as guanosine (G) during translation, enabling A-to-G changes at the transcriptome level.[115] In 2020, Cox et al. showed that a REPAIRv2 system (dCas13b-ADAR2dd) could correct a premature stop codon in the MECP2 gene, implicated in Rett syndrome, in human cells with high specificity.[112] This system also enables applications like targeting mutant huntingtin mRNA in Huntington's disease.[116] In addition to RNA cleavage and RNA base editing, the Cas13 system has been developed to perform RNA detection via the specific high-sensitivity enzymatic reporter unlocking; the same will be described later in this review.[117,118] Cas13's collateral cleavage activity has been exploited for antiviral applications against RNA viruses, including SARS-CoV-2.[119] A comparison table between different CRISPR-Cas systems has been tabulated in Table 2.

CRISPR-Cas in gene functionalization assay

Loss of function

CRISPR knockout and CRISPRi approaches have generated a loss-of-function (LOF) pooled CRISPR library. These are produced by transducing a large volume of cells with a pool of viruses carrying different CRISPR, cloned at low multiplicity of infection to ensure each cell receives only one.[120] Positive and negative selection-based LOF libraries have been generated. These libraries were subjected to selection for proliferation, drug sensitivity, or other parameters. The genomic profile of cells was analyzed before and after selection pressure, and a relative comparison of the expression was made through polymerase chain reaction sequencing and deep sequencing. These LOF screens led to the identification of essential and cancer-lethal genes.[121] The former included the genes involved in DNA replication, protein synthesis, the cell cycle, and proteolysis machinery. In addition, it also included several such genes for which very little information is available. On the other hand, cancer-lethal genes included genes involved in signaling, differentiation, and other regulatory processes.[122]

LOF libraries were a boon in identifying crucial individual genes and discovering and establishing the relationship between the genes in specific cancer types. Apart from establishing reliance on genes, this has been pivotal in identifying the drug target specific to cancer. Examples include breakpoint cluster region and Abelson murine leukemia viral oncogene homolog lethal mutations identified in chronic myelogenous leukemia Kawasaki medical school-7 (KBM7) cells, and Kirsten rat sarcoma viral oncogene homolog (KRAS) and

phosphatidylinositol-4,5-bisphosphate 3-kinase catalytic subunit alpha mutations in colon cancer cell lines human colon tumor 116 and Dukes' type C colorectal adenocarcinoma-1.[123,124] Identification of such cancer-specific lethal mutations has been of significant interest.

In addition to gene dependencies, LOF libraries have been crucial in identifying chromosome dependencies in Burkitt's lymphoma. Raji cells, which depend on DDX3Y present on the Y chromosome instead of its lost paralog DDX3X present on the X chromosome, are required in KBM7 and K562. This is attributed to the loss of DD3X due to inactivation following a mutation at the 5′ end.[125]

Gain of function

Gain-of-function (GOF) libraries are based on the CRISPRa. It is advantageous to the cDNA overexpression libraries and helps identify both the positively and negatively regulated genes in cancer cell proliferation.[70] A study performed on K652, a chronic myeloid leukemia cell line, showed inhibition of cancer cell proliferation upon activation of the anti-cancer proliferation gene due to the binding of CRISPRa. Though functionally intact, these antiproliferative genes mainly remain downregulated in cancer. Such anti-proliferation genes could serve as a potential target for drug therapy. Another CRISPRa screen performed in the A375 identified genes that impart resistance to vemurafenib.[126]

Both the LOF and GOF screens have been helpful in identifying the genes involved in the survival of normal cells, and also in discovering the cancer-lethal genes.[70] Further, these findings have been essential in identifying the novel protein dependencies in several cancer cell lines. The comprehensive application of different CRISPR screen types has revealed distinct categories of cancer-lethal genes across various functional pathways. Table S1 summarizes the cancer-lethal genes identified through different CRISPR screening approaches, organized by screen type and functional category. Further, these screens are useful in discovering the mechanism for drug resistance, mentioned later in this review.

Long non-coding RNA

The long non-coding RNAs (lncRNAs) are emerging as target molecules in studying cancer regulation and expression.[127] CRISPRi screens have been reported to be useful in identifying the lncRNA loci, which were difficult to identify with the RNAi approaches.[128] The interference screening identified 499 lncRNAs, of which approximately 89% were single-cell line specific. The plasmacytoma variant translocation 1 (PVT1) inhibition was identified as conferring a pro-growth phenotype to cancer cells when CRISPRi targeted the 1 kb region around the transcription start site. This unexpected finding revealed that the PVT1 promoter functions as a DNA boundary element that normally suppresses myelocytomatosis viral oncogene homolog (MYC) expression through a promoter competition mechanism.[129] The PVT1 and MYC promoters, located 55 kb apart on chromosome 8q24, compete for engagement with four intragenic enhancers within the PVT1 locus. Under normal conditions, these enhancers preferentially contact the PVT1 promoter, thereby limiting MYC transcription. However, when PVT1 promoter activity is silenced via CRISPRi, these enhancers redirect their interactions to the MYC promoter, resulting in increased MYC expression and enhanced cell proliferation.[130]

The pro-growth phenotype induced by PVT1 promoter silencing was highly correlated with the degree of MYC upregulation, demonstrating that MYC serves as the primary effector of this growth advantage.[130] This mechanism operates in *cis*, with the PVT1 promoter inhibiting MYC expression only from the same chromosome through promoter competition.[129] Importantly, this tumor suppressor function is independent of the PVT1 lncRNA transcript, as strategies that degraded the PVT1 RNA without affecting promoter activity failed to increase MYC expression or cell growth.[129]

Similarly, long intergenic non-coding RNA 00263 (LINC00263), which was upregulated in several cancer cell lines, showed differential effects across cell types when targeted by CRISPRi. Upon inhibition in U87 glioblastoma cells, LINC00263 suppression led to upregulation of endoplasmic reticulum stress response genes and apoptosis genes. However, no similar effect was observed in other cell lines examined, including K562, Michigan Cancer Foundation-7, MD Anderson-metastatic breast-231, and Henrietta Lacks cells.[131] This cell-type specificity underscores the importance of context-dependent lncRNA functions and highlights why functional screening across multiple cell types is essential for understanding lncRNA biology.

Epigenetic modifications

CRISPR has not only served as a tool to study genetic modification but also as a tool of choice to study epigenetic regulation.[132] This is often done by fusing the dCas protein with the targeted modification under study. One of the most frequently studied modifications includes methylations and demethylation events, resulting in suppression and activation of gene function. dCas9-DNMT3 and dCas9-SunTag-DNMT3 are routinely used modifications to study the effect of methylation on gene function.[133] The former modification generates weak methylation events, whereas the latter produces high-efficiency methyl modification. Gene-specific sgRNA, dCas9 fused with DNA methyltransferase, binds to the promoter or the enhancer of the gene, resulting in methylation of the CpG islands thus causing gene silencing by recruitment of the repressive chromatin modifier to the site of modification, heterochromatin formation, and halting of the transcription machinery by preventing the binding of transcription factors and RNAPII at the promoter site.[134,135] These modifications have been used to study the effect of the hypermethylated regulatory region of tumor suppressor genes such as phosphatase and tensin homolog deleted on chromosome 10 (PTEN), cyclin-dependent kinase inhibitor 2A, and RAS association domain family 1 and their effect on oncogenesis.[136,137] Similarly, Vojta et al. used dCas9-DNMT3A to methylate the promoter of BTB domain and CNC homolog 2, resulting in significant transcriptional repression.[138] On the other hand, demethylation events at the gene site can be studied by adding TET1 to the dCas9 as dCas9-TET1 or to

dCas9-SunTag-TET1 and dCas9-MCP-TET1.[86] dCas9-TET1 system uses TET1 as the demethylation enzyme, causing oxidation of 5-methyl cytosine, resulting in demethylation at the target promoter site. This strategy usually targets the hypermethylated regions of the genes suppressed epigenetically, such as the tumor suppressor gene and genes involved in stemness. The demethylation causes relaxation of the chromatin and facilitates the recruitment of RNAPII machinery.[139] Cas9-TET1 system was utilized to reprogram the neural stem cells. CRISPR modifications have also been useful in studying the effect of acetylation on gene function. dCas9-p300 fusion protein has been used to study the effect of acetylation on octamer-binding transcription factor 4 and myogenic differentiation 1 in human embryonic kidney-293 cells with SV40 T antigen cells.[84] The study shows that binding results in the significant upregulation of gene expression. Furthermore, not limited to these modifications, CRISPR has played an essential role in identifying the proteins involved in chromatin remodeling using CRISPR affinity purification *in situ* of regulatory elements and CRISPR-associated protein extraction (CASPEX).[140] The former take a long incubation time to identify the protein based on the avidin-streptavidin interaction, whereas the latter can capture even the dynamic processes. The CASPEX system consists of dCas9-ASPEX peroxidase added into the cells in a mixture of hydrogen peroxide and biotin-phenol.[101] Production of free radicals due to peroxidase activity results in the attachment of biotin to the protein present in the vicinity, which could be analyzed via pull-down assays. In addition, the CRISPRi approach has also been helpful in identifying distal regions located far away but essential for gene function. The system is referred to as chromatin locus optogenetic with CRISPR-dCas9 and makes use of two chimeric dCas9 molecules.[72] One targets the distal region, and the other targets the promoter site. These dCas9 contain dimerization domains of phytochrome-like 1 and ABA-insensitive 1, which dimerize on the addition of abscisic acid, which acts as an inducer.[141] Thus, it is useful in establishing distal regulation, which is important in several cancer-associated regulations. Chromatin remodeling plays a crucial role in gene regulation and can be precisely manipulated using CRISPR-Cas systems. By linking catalytically inactive Cas9 (dCas9) to chromatin remodeling complexes, scientists can target specific genomic regions to shift nucleosomes and modify local chromatin architecture.[142] This strategy enables access to genes that are typically silenced due to dense chromatin packing. Such targeted remodeling holds promise for reactivating suppressed genes and developing treatments for diseases driven by epigenetic repression.[143]

In summary, CRISPR-based epigenetic editing offers a powerful, reversible strategy for precise gene regulation, with significant potential in research and therapeutic applications. However, issues such as off-target effects, cytotoxicity, transient activity, and dependence on chromatin context remain key limitations that must be addressed to fully harness its clinical utility.[144,145]

CRISPR integration with advanced molecular techniques

While CRISPR-Cas systems have revolutionized genome editing, their true potential is realized when integrated with other advanced molecular techniques, particularly proteomics, transcriptomics, and metabolomics approaches.[146] This multi-omics integration provides comprehensive insights into cellular function that cannot be achieved through genetic manipulation alone. Proteomics techniques offer distinct advantages that complement CRISPR-based genetic studies. A multi-omics integrative analysis based on CRISPR screens enables redefinition of pluripotency regulatory networks through integration of functional genomics, transcriptomes, proteomes, and epigenome data. CRISPR-Cas technologies are increasingly applied to proteomics through three major approaches: studying protein-protein interactions, studying protein-chromatin interactions, and generation of cellular models with subsequent proteomic analysis.[147] Unlike CRISPR knockout studies that examine gene function through LOF approaches, mass spectrometry-based proteomics captures the dynamic protein landscape, including isoform-specific changes and temporal protein regulation that may not be apparent from genetic studies alone.[148] The integration of CRISPR with single-cell technologies has proven particularly powerful.[149] Perturb-seq (also known as CRISP-seq and CROP-seq) combines multiplexed CRISPR-mediated gene inactivation with single-cell RNA sequencing to assess comprehensive gene expression phenotypes for each perturbation.[150] Recent advances include PerturbSci-Kinetics, which captures whole transcriptomes, nascent transcriptomes, and sgRNA identities across hundreds of genetic perturbations at the single-cell level.[151]

Clinical trials increasingly utilize proteomic biomarkers for monitoring CRISPR therapy efficacy, with blood testing used to assess if genome-editing components are successfully reducing target protein levels. Mass spectrometry-based proteomics is being implemented in clinical laboratories for biomarker discovery, early detection, prognosis, and treatment response monitoring in CRISPR-treated patients.

CRISPR-Cas delivery systems

CRISPR-Cas therapeutic potential depends on efficient delivery to target cancer cells, with method choice varying by objectives and tissue. Effective delivery vehicles must (1) resist immunological clearance, (2) accumulate in target tissues and undergo endocytosis, and (3) escape lysosomal degradation.[152] Current delivery approaches can be broadly classified into three categories, which include physical methods, viral vectors, and non-viral systems (Figure 4).[153]

Physical delivery methods

Electroporation uses electric pulses to create transient membrane pores, facilitating entry of CRISPR-Cas components, achieving >90% editing efficiency in *ex vivo* chimeric antigen receptor (CAR)-T cell engineering, particularly targeting CD19 in B cell leukemias.[154] Microinjections are another prominent technique that enables the precise delivery of the CRISPR components into individual cancer cells. It is commonly used for generating cancer cell models with specific oncogene knockouts like TP53 or KRAS.[155] Advanced techniques such as magnetofection combine magnetic nanoparticles

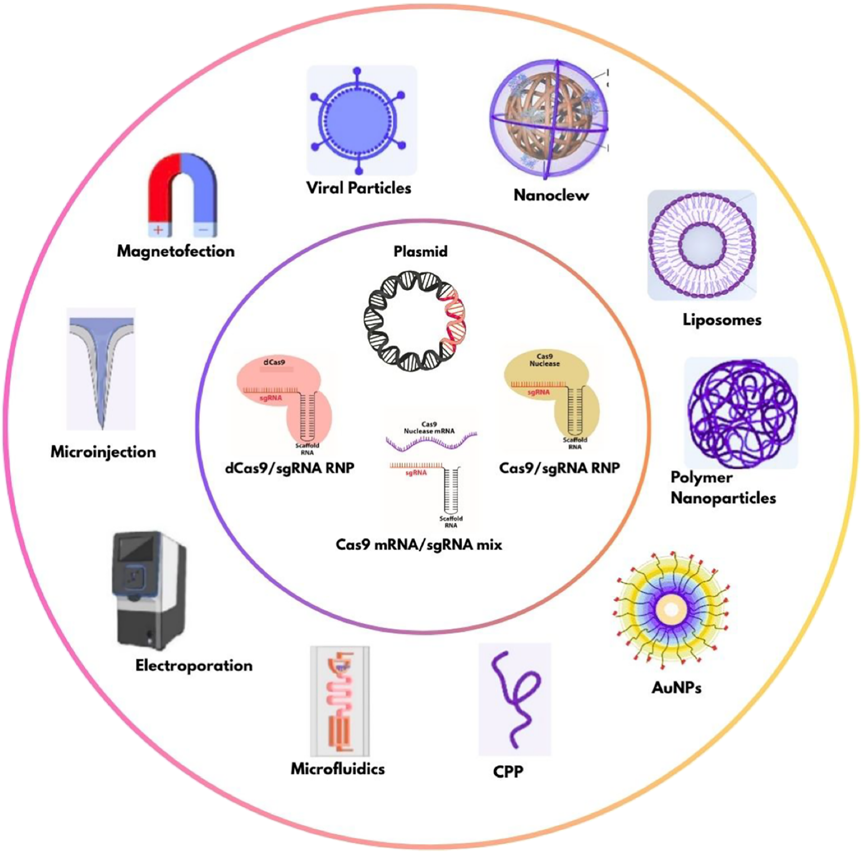

Figure 4. CRISPR-Cas delivery systems and cargo types

The inner circle illustrates different CRISPR cargo formats, including dCas9/sgRNA RNP complexes, Cas9/sgRNA RNP complexes, plasmid DNA, and Cas9 mRNA/sgRNA mixtures that can be delivered to target cells. The outer circle depicts various delivery vehicles and methods, including physical delivery methods (electroporation, microinjection, and magnetofection), viral vectors (viral particles), and non-viral carriers (liposomes, polymer nanoparticles, AuNPs, nanoclews, microfluidics, and CPPs). Each delivery system offers distinct advantages for specific applications in CRISPR-mediated genome editing. RNP, ribonucleoprotein; dCas9, dead Cas9; sgRNA, single-guide RNA; mRNA, messenger RNA; AuNPs, gold nanoparticles; CPPs, cell-penetrating peptides.

with CRISPR components using magnetic fields for tumor targeting, demonstrating enhanced delivery to breast cancer xenografts.[156]

Viral vector methods

Viral vectors are preferred gene delivery systems due to their natural cell entry ability and high efficiency. Adeno-associated viruses (AAVs) offer low immunogenicity, broad tissue tropism, and long-term episomal expression in non-dividing cells. CRISPR-based AAVs have been used to successfully target liver metastases by knocking out tumor suppressors like PTEN in hepatocellular carcinoma.[157] However, packaging limitations (~4.7 Kbp) restrict larger Cas proteins like SpCas9 (~4.9 Kbp).[158] Himeda et al. used dSaCas9 variants to inhibit DUX4 mRNA in facioscapulohumeral muscular dystrophy.[159] Lentiviral vectors provide a 10 kb capacity suitable for packaging complex cancer gene editing machinery. Lentivirus-based CRISPR systems were used to effectively disrupt oncogenes like MYC in lymphomas and deliver large donor templates for tumor suppressor restoration.[160] However, random genomic integration poses insertional inactivation risks and potential immune responses. Adenoviral vectors offer 36 kb capacity for comprehensive cancer gene editing. It successfully targets multiple oncogenes simultaneously in lung cancer models, despite immunogenicity limiting systemic use.[161] Baculoviruses, non-pathogenic insect viruses, maintain episomal expression without replication or genomic integration. Nguyen et al. used baculoviruses carrying dCas9-VP64-p65-Rta to enhance lncRNA expression significantly.[162]

Non-viral delivery methods

Lipid-based systems

Lipid nanoparticles (LNPs) are prominent non-viral CRISPR-Cas carriers due to their biocompatibility, low immunogenicity, and versatility in packaging nucleic acids, proteins, or RNP complexes. LNPs contain four primary lipids: ionizable cationic lipids, PEG lipids, zwitterionic phospholipids, and cholesterol, entering cells via endocytosis. Finn et al. (2018)[163] achieved a successful editing in over 70% of mouse liver cells by targeting the TTR gene using LNPs to deliver Cas9 mRNA and sgRNA.[164] However, LNPs accumulate primarily in the liver due to lipid metabolism, benefiting liver-targeted studies but limiting tissue tropism. Some studies overcome this by incorporating tissue-specific binding proteins onto LNPs.[165] Intellia's NTLA-2001 uses LNPs for liver-targeted editing, while cancer applications include LNP-delivered CRISPR targeting PCSK9 for familial hypercholesterolemia-related cardiovascular risks.[166] Recent studies achieved 16%–37% editing efficiency in lung cancer models with tissue-selective LNPs.[167,168] Liposomes offer excellent biocompatibility for cancer applications, with targeted versions delivering CRISPR to HER2-positive breast cancer cells for specific knockout of drug resistance genes like MDR1.[169]

Polymeric systems

Polymer-based nanoparticles offer chemical adaptability, biocompatibility, and nucleic acid/protein protection from degradation. The carriers include natural or synthetic polymers like chitosan, Polyethylenimine (PEI), Poly lactic-co-glycolic acid (PLGA), or Poly beta-amino esters (PBAEs), varying by target site requirements.[170] Their surfaces can be functionalized to enhance cell targeting or endosomal escape, improving intracellular delivery efficiency. PLGA nanoparticles modified with chitosan enhance cancer cell uptake.[171]

This demonstrates successful glioblastoma delivery for epidermal growth factor receptor mutation targeting with 80% encapsulation efficiency and sustained release.[172] PEI-based systems effectively deliver CRISPR plasmids to cancer cells. PEI-magnetic nanoparticles have been used to target melanoma cells for BRAF knockout, outperforming the traditional lipofectamine-based approach in A375 cells.[173] Unlike viral vectors, polymeric nanoparticles reduce insertional mutagenesis risk and can degrade under physiological conditions for controlled cargo release.[174]

Biological delivery systems

Exosomes are membrane-bound vesicles (30–100 nm diameter) originating from multivesicular bodies in organelles that serve as natural intercellular biomolecule carriers.[175] They offer a higher carrying capacity for both sgRNA and Cas systems together, minimizing off-target effects.[176] Since exosomes retain parent cell proteins and lipids, they interact preferentially with parent cell molecules, providing selectivity. Wan et al. engineered hepatic stellate cell-derived exosomes to target PUMA, cyclin E1, and K acetyltransferase 5 responsible for disease development.[177] Cancer-derived exosomes deliver CRISPR systems to suppress Poly ADP-ribose Polymerase-1 (PARP-1) in ovarian cancer models, achieving complete growth inhibition.[178] Mesenchymal stem cell exosomes target glioblastoma cells for MGMT knockout, enhancing temozolomide sensitivity.[179] Cell-penetrating peptides (CPPs) facilitate direct membrane crossing. Engineered CPPs deliver Cas9-sgRNA complexes to pancreatic cancer cells for KRAS G12D correction, achieving 23%–30% editing efficiency.[180]

Inorganic systems

Gold nanoparticles (AuNPs) offer optical tracking, easy surface modification, and biocompatibility. Their high surface-area-to-volume ratio enables efficient loading of Cas9 protein, gRNA, or DNA templates.[181] Wang et al. combined lipid nanoparticles with AuNPs for targeted delivery, increasing uptake and target efficiency.[182] Glucose-coated AuNPs deliver CRISPR systems to brain tumors, enabling real-time glioblastoma editing monitoring through glucose transporter overexpression.[183] Nanoclews are DNA-based carriers that efficiently package CRISPR components for breast cancer targeting, achieving controlled release and enhanced specificity versus free CRISPR delivery.[184]

Current clinical trials focus on *ex vivo* CAR-T cell engineering using electroporation, while *in vivo* applications utilize LNPs for liver-targeted editing. Future cancer applications include combination approaches like exosome-liposome hybrids for dual oncogene targeting and tissue-specific promoters for enhanced tumor selectivity.[185] Method choice depends on cancer type, target accessibility, and required editing precision, with physical methods dominating *ex vivo* applications and non-viral systems preferred for *in vivo* tumor targeting.

Advances in organoid and animal model development using CRISPR

CRISPR has revolutionized gene functionalization studies and transformed the development of complex biological models like organoids and genetically engineered animals.[186] These models offer unprecedented opportunities to mimic human physiology and disease more accurately than traditional cell lines.[187] Organoids, derived from adult or pluripotent stem cells, recapitulate tissue architecture and function, enabling the study of development, disease progression, and therapeutic responses.[188] When generated from patient samples, they serve as personalized platforms for drug screening and precision medicine. CRISPR has accelerated animal model creation through rapid, targeted genome modifications essential for studying gene function, modeling human diseases, and testing gene therapies *in vivo*.[189,190] Together, CRISPR-powered organoid and animal models are reshaping biomedical research, providing deeper disease insights and facilitating translation of genetic discoveries into clinical applications.[191]

Drost et al. and Matano et al. independently transformed healthy colon organoids into cancerous counterparts.[192,193] Colon cancer was generated through sequential adenocarcinoma involving the disruption of tumor suppressors TP53, SMAD4, and Adenomatous Polyposis Coli (APC), plus base editing in oncogenes KRAS and PI3K.[193] These cells grew independently of the growth factors required by normal organoids.

CRISPR-Cas systems are essential for generating cancer animal models.[194] Studies use either transplanted *ex vivo*-edited cells or *in vivo* sgRNA/Cas complex delivery targeting desired genes. Cas12-based systems can target multiple genes simultaneously. Transplantation of *ex vivo*-edited cells enables high-throughput screening for LOF/GOF associated with cancer-lethal genes. Chen et al. performed genome-wide LOF screening to identify lung metastasis genes, transducing non-metastatic murine lung cancer cells with pooled CRISPR libraries and transplanting them to mice.[195] Similarly, LOF screening with non-functional TP53 and overexpressed MYC identified four novel liver cancer progression genes: Nf1, Flrt1, Plxnb1, and B9d1.[196]

In vivo studies involving the delivery of plasmid targeting multiple genes simultaneously, via Cas9/sgRNA, rather than requiring multiple breeding rounds. This approach created models with non-functional PTEN and TP53, and targeted ten genes leading to hepatocellular carcinoma development.[197,198] CRISPR strategies have significantly advanced precise epithelial ovarian carcinoma mouse model generation, aiding ovarian cancer mechanism and therapy investigation.[199] These studies explore gene interrelationships and dependencies for functional phenotypes and drug/inhibitor effects.

CRISPR-Cas in drug resistance exploration

LOF/GOF screens performed at *in vitro* and *in vivo* levels have become a critical tool in discovering drug resistance in cancer, the genes involved, and the mechanism. The topoisomerase II alpha (TOPIIA) and cyclin-dependent kinase 6 were identified as positive screens in the LOF screens. These genes impart resistance to the HL60 against the TOPIIA inhibitor, etoposide.[124] Similarly, neurofibromin 2, cullin E3 ligase, and members of the Spt-three-Taf9-Ada-Gcn5 acetyltransferase histone deacetylase complex were identified

as the genes imparting resistance against B-Raf proto-oncogene, serine/threonine kinase (BRAF) inhibitor in the A375 melanoma cell line.[200] A GOF study conducted on A375 using BRAF inhibitors showed that the inhibition could be revoked by reactivation of the mitogen-activated protein kinase pathway or other parallel pathways.[126] Further, they have been essential in discovering the role of mutations responsible for imparting resistance to the inhibitor.

CRISPR-Cas as a diagnostic tool

Demand for highly sensitive, rapid, and cost-effective nucleic acid detection has increased significantly, especially in cancer diagnostics. Traditional approaches, including tissue biopsy, imaging, and blood-based tumor marker detection, remain cornerstones of clinical diagnosis. While effective, these methods are often invasive, time-consuming, and sometimes lack sensitivity for detecting early-stage malignancies or subtle genetic alterations. Next-generation sequencing marked a major advancement through genome-wide mutation profiling; however, routine diagnostic application is limited by cost, infrastructure requirements, and turnaround time.

CRISPR-based diagnostics have emerged as transformative solutions offering rapid, specific, and scalable alternatives.[201,202] Certain CRISPR-associated enzymes, particularly Cas12 and Cas13, have been harnessed for molecular detection.[203] Cas13a, upon recognizing specific RNA sequences, undergoes conformational changes, activating collateral cleavage activity that indiscriminately cuts nearby single-stranded RNAs.[118] Similarly, Cas12a exhibits collateral cleavage on single-stranded DNA following target-specific double-stranded DNA binding.[204] This target-triggered non-specific cleavage phenomenon forms the basis of CRISPR-powered diagnostic platforms.

Specific high-sensitivity enzymatic reporter unlocking utilizes Cas13a for RNA or DNA detection with attomolar sensitivity, while DNA endonuclease-targeted CRISPR trans reporter employs Cas12a for highly specific DNA sequence detection.[204] These platforms enable rapid identification of cancer-associated mutations, infectious agents, and other biomarkers with high precision, even in resource-limited settings. CRISPR diagnostics represent a significant advancement in next-generation point-of-care technologies with broad applications in oncology, infectious diseases, and genetic screening. Table 3 summarizes CRISPR-Cas12 and Cas13-based diagnostic platforms, highlighting their mechanisms and applications in sensitive, efficient nucleic acid detection.

Advancements in CAR-T cell therapy

Immunotherapies have brought a new ray of light to cancer treatment and have been instrumental in saving the lives of many people across the globe.[205] Current-day immunotherapies are not merely focused on the generation of monoclonal antibodies blocking the aberrant ligands; therapies that make use of engineered T cells have also gained much popularity in the field of cancer therapeutics.[206] CRISPR-engineered CAR-T cell therapy uses Cas9 to enhance T cell persistence by disrupting key immune checkpoint genes such as programmed death-1 (PD-1), cytotoxic T lymphocyte-associated protein 4 (CTLA-4), and lymphocyte activation gene 3 (LAG-3), which normally limit T cell activation and promote exhaustion in the tumor microenvironment. Simultaneously, Cas9 facilitates the precise insertion of CARs targeting tumor-specific antigens like CD19, CD20, or HER2 into safe harbor sites such as the T cell receptor alpha constant (TRAC) locus, creating enhanced T cells capable of sustained anti-tumor activity.[207] Clinical trials are being conducted in several parts of the world, and many of them have received FDA approval.[208] CRISPR therapies focus on generating *ex vivo*-edited cells known as the CAR-T cell (Figure 5). These therapies are focused on activating the co-stimulatory pathway and blocking the inhibitory pathway in the edited cells. In the former approach, T cells are engineered to express chimeric antigens having a scFv targeting the tumor antigen and fused with the T cell signaling domain CD3ζ and FcRγ for activation. The second- and third-generation therapies using this approach express additional co-stimulatory molecules such as CD27, CD28, and 4-1BB. This approach has successfully recovered patients suffering from B cell leukemia and other lymphomas. Here, CD19-targeting chimeric antigen-expressing T cells were produced, with some trials incorporating co-stimulatory CD27 and CD28 for increased efficiency.[209] These clinical trials targeting B cells received FDA approvals. Similar to this, several clinical trials targeting various cancerous cell-specific proteins are being conducted. These include HER-2, GD2, CD70, CD171, and many more.

The inhibitory approach, on the other hand, targets downregulation of the specific receptor expressed on the T cell surface that is utilized by cancerous cells for preventing tumor clearance. The interaction between PD-1 and programmed death-ligand 1 (PD-L1) in normal cells to distinguish the self from the non-self is one such example.[141] However, cancerous cells cleverly use this interaction to inhibit T cell activation even after T cell receptor (TCR) recognition of the target ligand. CAR-T therapy targets downregulation of the PD-1 receptor on the surface of T cells. Other receptors targeted using a similar approach include CTLA-4, LAG-3, adenosine A2A receptor ($A_{2A}R$), T cell immunoglobulin and mucin domain-containing protein 3, and indoleamine 2,3-dioxygenase.[210] PD-1 signaling modulation using CAR-T activation was inhibited following $A_{2A}R$ activation. Downregulation of the $A_{2A}R$ resulted in the inhibition of tumor proliferation, highlighting the importance of co-targeting several pathway proteins together, resulting in the inhibition of the same. In addition, CAR-T engineering can also be done to produce αPD-1-secreting cells that help neutralize circulating cancerous B cells.[211] Several PD-1 modification strategies have emerged to enhance CAR-T cell efficacy. Direct gene knockout using CRISPR-Cas9 technology eliminates PD-1 expression entirely, preventing inhibitory signaling and maintaining T cell activation in immunosuppressive environments.[212,213] Alternative approaches include engineering dominant-negative PD-1 receptors that retain surface expression but lack functional signaling capacity, effectively blocking endogenous PD-1 pathways without complete gene deletion.[214] A particularly innovative strategy involves creating PD-1-CD28

Table 3. This table summarizes CRISPR-based diagnostic platforms, focusing on Cas12a and Cas13a, their target nucleic acids, and the mechanisms enabling collateral cleavage for sensitive and efficient nucleic acid detection

Sr. No.	CRISPR diagnostic system	CRISPR enzyme	Target	Mechanism	Application
1	SHERLOCK (specific high-sensitivity enzymatic reporter unlocking)	Cas13a	RNA	Cas13a binds to specific RNA sequences; upon binding, it undergoes a conformational change, activating collateral cleavage of non-target ssRNA	sensitive RNA detection, including detection of genetic mutations, viral RNA (e.g., cancer biomarkers, and pathogens), and other genetic alterations
2	DETECTR (DNA endonuclease-targeted CRISPR trans reporter)	Cas12a	DNA (dsDNA)	Cas12a recognizes and binds to target DNA, triggering a conformational change and collateral cleavage of ssDNA	sensitive DNA detection, including identification of specific mutations, pathogens, or other genetic markers, commonly used for diagnostic applications such as SARS-CoV-2
3	CDetect	Cas12a	DNA	Cas12a binds to specific DNA targets, causing collateral cleavage of ssDNA, and utilizes a fluorophore-quencher system for signal detection	DNA-based detection system that can be used for the identification of bacterial or viral DNA, genetic mutations, and diagnostics of inherited diseases
4	CRISPR-Cas12-based detection of SARS-CoV-2	Cas12a	RNA/DNA	Cas12a detects viral RNA or DNA by binding to the target sequence, activating collateral cleavage of ssRNA or ssDNA	rapid, point-of-care detection of SARS-CoV-2, the virus causing COVID-19, enabling fast and efficient diagnostic workflows in clinical settings
5	CASSANDRA (CRISPR-Cas system for amplified nucleic acid detection)	Cas13a	RNA	Cas13a recognizes RNA targets, triggering a conformational change and collateral cleavage; this is then quantified by fluorescent signals or other methods	RNA-based diagnostics for infectious diseases, including the detection of viruses (e.g., Zika and Dengue) and bacterial infections
6	CARMEN (CRISPR-based amplified RNA monitoring)	Cas13a	RNA	Cas13a activates collateral cleavage upon recognition of RNA, resulting in the release of a fluorescent signal that indicates target presence	used for RNA-based disease diagnostics, including early-stage cancer detection, genetic screening, and pathogen detection
7	COLONY (CRISPR-based optical localized nucleic acid yield)	Cas13a	RNA	Cas13a binds to RNA targets and induces collateral cleavage, producing fluorescence or other signals to detect specific genetic sequences	real-time monitoring of RNA sequences for disease monitoring, including viral infections and genetic disorders
8	TAM (targeted amplification monitoring)	Cas12a	DNA	Cas12a's target binding leads to DNA cleavage, causing the release of fluorophores or quenching agents	used for rapid and highly sensitive DNA amplification and detection, especially in clinical diagnostics, such as for HIV or tuberculosis

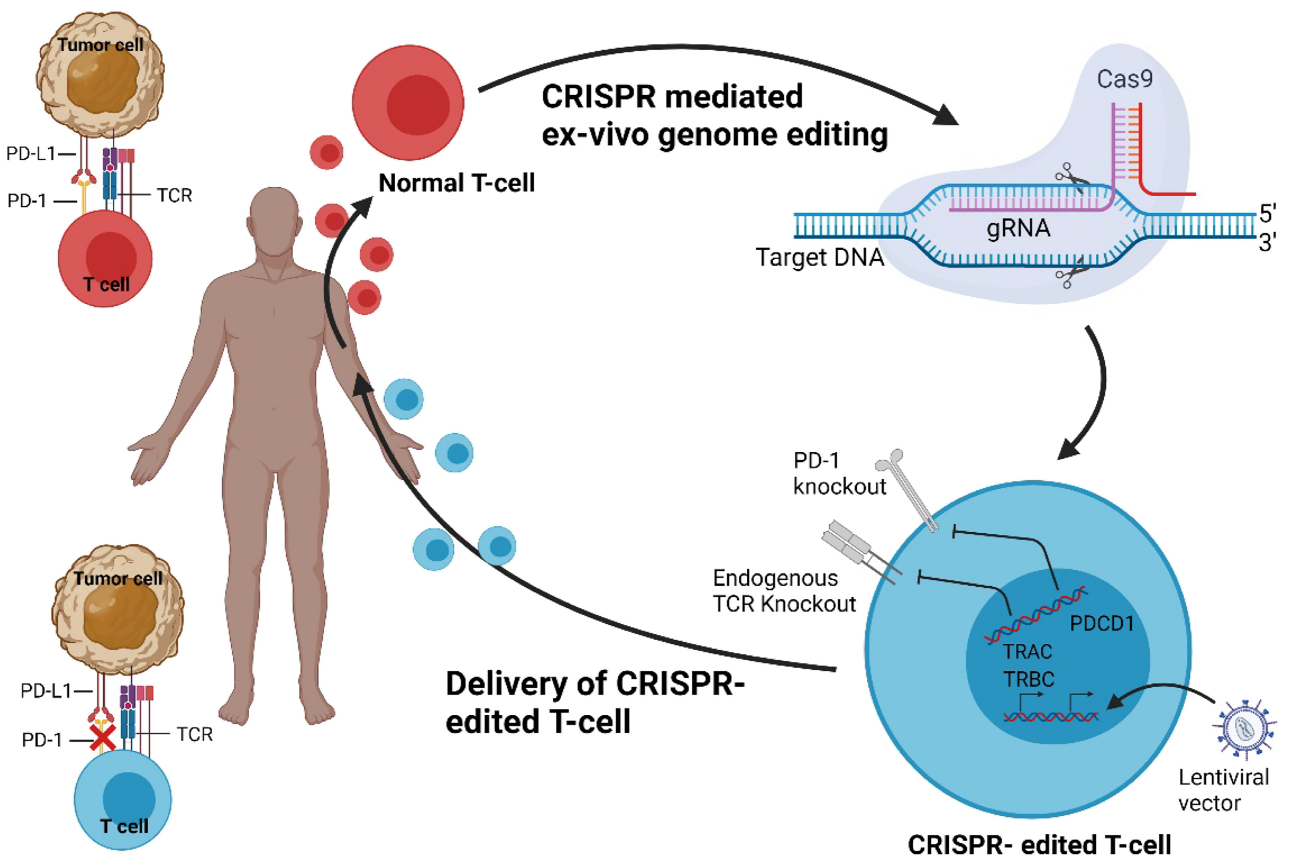

Figure 5. An overview of CRISPR-Cas-engineered T cell therapy

A sufficient amount of blood is drawn from a cancer patient to obtain enough peripheral blood mononuclear cells (PBMCs) for engineered T cell manufacturing. The T cells are purified from PBMCs. After *in vitro* activation and amplification, the CRISPR-Cas9 RNP complexes loaded with three sgRNAs are electroporated into normal T cells. This results in gene editing of the TRAC, TRABC, and PDCD1 (encoding PD-1) loci. The T cells are then transduced with a viral vector, such as lentivirus, to express a TCR specific for the cancer antigens. Following amplification and quality control, CRISPR-edited T cells are infused intravenously into the patient's body to improve antitumor ability. PBMCs, peripheral blood mononuclear cells; sgRNAs, single-guide RNAs; TRAC, T cell receptor alpha constant; TRABC, T cell receptor beta constant; PDCD1, programmed cell death 1; PD-1, programmed death-1; TCR, T cell receptor.

chimeric receptors that convert inhibitory PD-L1 binding into costimulatory signals, transforming the tumor's immune suppression mechanism into a T cell activation pathway.[214] These modifications collectively address T cell exhaustion and dysfunction commonly observed in solid tumor microenvironments.[212,213] Clinical trials demonstrate that PD-1-modified CAR-T cells exhibit enhanced persistence, improved tumor infiltration, and superior antitumor activity compared to conventional CAR-T therapies, particularly in challenging solid tumor contexts where immunosuppression limits therapeutic efficacy.[211,215] However, the clinical implementation of CRISPR-engineered CAR-T cell therapies faces significant manufacturing challenges.[216] The process requires specialized Good Manufacturing Practice (GMP) facilities, typically takes 2–4 weeks from cell collection to final product, and costs an estimated $100,000–$500,000 per patient in manufacturing expenses alone.[217] These logistical complexities currently limit treatment to major academic medical centers, creating access disparities for patients in rural or underserved areas.

MEGA-CRISPR: Revolutionizing cancer immunotherapy

MEGA-CRISPR (multiplexed enhanced genome engineering via Cas-responsive self-processing RNA) is a tool for next-generation genome editing, designed to make multiple gene edits at once using a single RNA transcript, thus eliminating the need for multiple RNAs.[218] The system utilizes the presence of a cleavage signal between the RNA molecules to generate multiple sgRNAs targeting different genes. Traditional single-target approaches often fail because cancer employs multiple resistance mechanisms simultaneously.[219] MEGA-CRISPR's ability to target numerous pathways concurrently represents a paradigm shift toward comprehensive cancer treatment strategies. This multigene target strategy has also boosted the CAR-T therapies.[219] Before the MEGA-CRISPR approach, CAR-T therapies showed limited effect in solid tumors as these were marked by the problems of low T cell persistence, T cell exhaustion, and immune suppression. MEGA-CRISPR allows overcoming this barrier by not just targeting genes such as PD-1, LAG-3, or TIGIT, which restrict the T cell activity, but also inserting

the CAR gene into a safe location like the TRAC locus, thereby improving the T cell survival, strength, and tumor-killing ability.[211,220] By disrupting PD-1 expression through MEGA-CRISPR editing, the engineered T cells maintain their aggressive anti-tumor activity even in immunosuppressive tumor microenvironments. In addition, the MEGA-CRISPR strategy provides an advantage of eliminating viral vectors, such as lentiviruses, which pose a biosafety risk as gene delivery vehicles, as it can be simply delivered using nanoparticle and electroporation strategies.[221] Further to providing advantages as a versatile tool in creating patient-specific CAR-T therapy, the approach also aims to develop "off the shelf" T cell therapies by editing immune-related genes such as TCRα (to prevent graft-versus-host disease) and human leukocyte antigen molecules (to avoid rejection) and then equipping them with a CAR.[222] Beyond checkpoint inhibitor disruption, the technology enables simultaneous integration of suicide genes for safety control, cytokine receptors for improved proliferation, and resistance genes against chemotherapy drugs used in conditioning regimens.[223] This multi-layered approach creates super-charged T cells with enhanced trafficking capabilities to tumor sites, improved metabolic fitness for sustained activity, and resistance to tumor-induced apoptosis signals. Clinical trials have demonstrated that these multiply edited CAR-T cells show superior expansion kinetics and prolonged persistence compared to conventional single-edit approaches.[224]

Clinical translation and global therapeutic progress

The therapeutic potential of CRISPR technology is evident from the rapidly expanding clinical trial landscape, with nearly 300 trials globally encompassing diverse disease areas and demonstrating remarkable success rates in advanced-stage studies (Table S2).[225,226] The landmark approval of Casgevy (CTX001) for sickle cell disease and β-thalassemia represents a watershed moment, achieving 95% transfusion independence in treated patients.[225] A landmark achievement in personalized medicine occurred in 2024 with the treatment of KJ Muldoon, a baby who became the world's first patient to receive a bespoke CRISPR gene-editing therapy designed specifically for his unique genetic mutation.[102] This success has catalyzed investment and research across multiple therapeutic domains, from genetic diseases to cancer immunotherapy. The global distribution of CRISPR trials reflects both regulatory maturity and research infrastructure, with North America leading in trial numbers (145 active trials), followed by Europe (89 trials). Notably, the focus areas vary by region, with North America emphasizing cancer applications (60% of trials), while Europe prioritizes hemoglobinopathies (40% of trials).[227] The high success rates observed in advanced trials (70%–95%) underscore the maturation of CRISPR technology from experimental tool to clinical reality.

Current challenges and limitations

Off-target effects and genomic safety

Off-target activity is a widely recognized limitation where Cas9 endonuclease induces DSBs at genomic sites resembling the intended target. These unintended edits can cause harmful mutations, chromosomal rearrangements, or oncogenic pathway activation.[228,229] Pharmacodynamic (PD) modifications improve CRISPR tool performance and duration at target sites once delivered, enhancing gene-editing accuracy, minimizing side effects, and ensuring system activation only when and where needed.

Major PD improvements include using precise Cas9 versions like SpCas9-HF1 or eSpCas9, engineered to reduce off-target effects through decreased non-specific cleavage activity for safer treatments.[31,230] Controlling timing and activity is advantageous where continuous regulation is not required. Inducible systems use split Cas9, divided into halves that reassemble in the presence of specific drugs or light signals, ensuring editing occurs only when needed and reducing prolonged or unwanted cellular harm.[231] Modulators fused with dCas9 in CRISPRa and CRISPRi approaches using KRAB and VP64, respectively, modulate gene expression rather than making permanent genomic changes. Cas13b-ADAR fusion-based base editing provides transient, reversible RNA editing options, minimizing off-target effects.[112]

Controlled editing strategies use destabilization domains causing rapid Cas9 degradation without stabilizing drugs (e.g., Shield-1), or deliver Cas9 protein/mRNA instead of plasmids for transient expression, ensuring efficient editing while minimizing prolonged activity.[232] These PD modifications make CRISPR tools more targeted, controllable, and safer, critical for transitioning from laboratory studies to real-world therapies, especially in complex diseases such as cancer or genetic disorders requiring precision. However, complete specificity remains challenging *in vivo*, where chromatin accessibility varies between tissues and cell types.

Delivery barrier

Efficient, targeted CRISPR component delivery remains a major bottleneck. Viral vectors like AAVs, widely used for higher transduction efficiency and long-term expression, have limited packaging capacity (∼4.7 kbp), creating problems for large Cas proteins or multiplex editing systems. AAVs and lentiviruses can trigger host immune responses and may integrate into genomes at low frequency, raising safety concerns.[233] Non-viral approaches, including LNPs, AuNPs, electroporation, and cell-penetrating peptides, offer reduced immunogenicity alternatives but often suffer from lower *in vivo* editing efficiency and limited tissue specificity.[234,235]

Immune response

Humans show pre-existing humoral and cellular immunity to SpCas9 or SaCas9, possibly from prior *Streptococcus pyogenes* or *Staphylococcus aureus* exposure.[236] This immune recognition can cause rapid clearance of edited cells or inflammatory toxicity, undermining therapeutic efficacy. Immunosuppressive strategies under investigation include using orthologs from less common bacteria or transient Cas9 delivery as protein or mRNA to address immune response issues.[237]

DNA repair pathway

DNA repair pathways activated following Cas9-induced DSBs influence gene-editing outcomes. Error-prone NHEJ is the predominant

mechanism in most cells, introducing indels useful for gene knockouts. However, precise gene correction or insertion requires HDR, which is inefficient and largely restricted to dividing cells, limiting use in post-mitotic tissues like neurons or cardiac muscle.[238] Additional challenges include embryonic mosaicism from early developmental stage editing, epigenetic effects of dCas9-based gene regulation, potential microbiota horizontal gene transfer, and unresolved ethical concerns around germline editing and gene therapy access equity.[239]

Safety and regulatory considerations

CRISPR clinical applications face off-target effects and potential chromosomal rearrangements following genome editing.[240,241] The FDA requires rigorous oversight with extensive preclinical testing and 15-year patient follow-up studies, as demonstrated in Casgevy's approval.[225] Viral vector delivery poses immunogenicity and insertional mutagenesis risks, necessitating careful vector design and patient monitoring.[242] Long-term CRISPR editing consequences remain largely unknown, particularly for *in vivo* applications. p53-mediated DNA damage responses following CRISPR-induced DSBs may affect genome stability and therapeutic outcomes.[243] On-target but unintended effects like chromothripsis-like rearrangements require comprehensive pre- and post-treatment genomic analysis.[244] High CRISPR therapy costs (approximately $2.2 million for Casgevy) raise equity concerns, limiting access to comprehensively insured patients.[245] Informed consent presents unique challenges due to long-term outcome uncertainty, particularly in pediatric applications like infant KJ Muldoon's personalized treatment.[102] Distinguishing therapeutic intervention from genetic enhancement requires ongoing societal dialogue and regulatory oversight.

International CRISPR governance coordination remains challenging, with varying national regulations creating regulatory arbitrage potential.[246] The WHO has established expert committees providing governance recommendations, emphasizing transparent oversight and public engagement in gene-editing policy development.[247] Developing standardized safety protocols, harmonized regulatory frameworks, and global access mechanisms will be crucial for realizing CRISPR's therapeutic potential while addressing legitimate ethical and safety concerns.

CONCLUSION

Cancer treatment remains one of the most significant and challenging areas in clinical medicine, as cancers are often driven by a multitude of genetic mutations. These mutations can lead to various oncogenic events such as the amplification of cell proliferation, the LOF of tumor suppressor genes, and the induction of metabolic changes that contribute to chemoresistance. Traditionally, cancer therapies have focused on conventional treatments like chemotherapy, radiation, and surgery. However, with the increasing understanding of cancer genomics, gene therapies are emerging as a transformative approach for treating a wide variety of cancers, with an emphasis on targeting the genetic underpinnings of tumors for more precise and personalized treatment. Gene therapy has made considerable strides due to advancements in bioinformatics, molecular biology, and genetic engineering technologies. These developments have paved the way for tools capable of precisely editing the genome, thus expanding the possibilities for cancer treatment. A key technology at the forefront of these advancements is the CRISPR-Cas system, which offers remarkable precision, versatility, and efficiency for genome editing. CRISPR-Cas systems have been widely employed in cancer research, enabling the creation of advanced *in vitro* and *in vivo* models that simulate human cancers more effectively than traditional methods. In addition, approaches such as MEGA-CRISPR have allowed simultaneous targeting of multiple genes regulating different pathways and have improved the therapeutic outcome. CRISPR-Cas system has also facilitated novel approaches to cancer diagnosis due to its versatility to detect both the DNA and RNA molecules using Cas9/Cas12 and Cas13, respectively, with the advanced system sensitive to even single-nucleotide differences. Despite the immense promise of CRISPR-based therapies in oncology, challenges remain. Efficient delivery of gene-editing tools into target tissues, minimizing off-target effects, and improving the long-term stability and precision of edits are areas that require continued research and innovation. Clinical trials involving CRISPR-Cas systems for cancer therapy are already underway, demonstrating the therapeutic potential of gene-editing technologies. However, before these therapies can be fully integrated into clinical practice, further studies are necessary to ensure their safety, efficacy, and scalability.

In conclusion, the evolution of CRISPR technology marks a significant turning point in cancer research and treatment. As these tools continue to improve, they hold the potential to revolutionize how cancer is diagnosed, studied, and treated, offering targeted, personalized therapies that could ultimately lead to more effective and sustainable cancer treatments. With ongoing advancements in precision medicine, it is becoming increasingly feasible to target the genetic alterations in tumors, offering hope for more effective therapeutic strategies. However, substantial efforts are still required to address the technical barriers and optimize the therapeutic applications of these powerful tools in clinical settings. The future of cancer therapy is closely tied to continued innovation in gene-editing technologies, making it an exciting frontier in modern medicine.

DATA AVAILABILITY

No new data were generated in this study. All information is derived from publicly available literature cited in the references.

ACKNOWLEDGMENTS

We gratefully acknowledge South Asian University and ICMR, India (2020-4505/CMB/ADHOC-BMS grant). The authors (V.P. and S.S.) acknowledge the fellowship support from the Department of Biotechnology, Ministry of Science and Technology, India.

AUTHOR CONTRIBUTIONS

V.P. and S.S. contributed equally to this work. V.P., S.S., and Y.R.P. jointly designed the review framework, analyzed the literature, and drafted the manuscript.

DECLARATION OF INTERESTS

The authors declare no competing interests.

SUPPLEMENTAL INFORMATION

Supplemental information can be found online at https://doi.org/10.1016/j.omtn.2025.102717.

REFERENCES

1. World Health Organisation (2021). Global Health Estimates: Life expectancy and leading causes of death and disability. https://www.who.int/data/gho/data/themes/mortality-and-global-health-estimates.
2. World Health Organization (2024). Global cancer burden growing, amidst mounting need for services.
3. Carbone, A. (2020). Cancer classification at the crossroads. Cancers (Basel) *12*, 10–15. https://doi.org/10.3390/cancers12040980.
4. Patrichi, A.I., and Gurzu, S. (2024). Pathogenetic and molecular classifications of soft tissue and bone tumors: A 2024 update. Pathol. Res. Pract. *260*, 155406. https://doi.org/10.1016/j.prp.2024.155406.
5. Pagano, L., Salmanton-García, J., Marchesi, F., Busca, A., Corradini, P., Hoenigl, M., Klimko, N., Koehler, P., Pagliuca, A., Passamonti, F., et al. (2021). COVID-19 infection in adult patients with hematological malignancies: a European Hematology Association Survey (EPICOVIDEHA). J. Hematol. Oncol. *14*, 1–15. https://doi.org/10.1186/s13045-021-01177-0.
6. Rebuli, M.E., Rose, J.J., Noel, A., Croft, D.P., Benowitz, N.L., Cohen, A.H., Crotty Alexander, L.E., Ewart, G., Goniewicz, M.L., Jaspers, I., et al. (2023). The E-cigarette or Vaping Product Use–Associated Lung Injury Epidemic: Pathogenesis, Management, and Future Directions An Official American Thoracic Society Workshop Report. Ann. Am. Thorac. Soc. *20*, 1–17. https://doi.org/10.1513/AnnalsATS.202209-796ST.
7. Cronin, K.M., Desai, A., Hookim, K., and Contino, G. (2024). Kaposi sarcoma in an individual recently diagnosed with HIV. IDCases *36*, e01961. https://doi.org/10.1016/j.idcr.2024.e01961.
8. Hanahan, D., and Weinberg, R.A. (2011). Hallmarks of Cancer: The Next Generation. Cell *144*, 646–674. https://doi.org/10.1016/J.CELL.2011.02.013.
9. Doll, R., and Peto, R. (1981). The Causes of Cancer: Quantitative Estimates of Avoidable Risks of Cancer in the United States Today. JNCI J. Natl. Cancer Inst. *66*, 1192–1308. https://doi.org/10.1093/JNCI/66.6.1192.
10. Arruebo, M., Vilaboa, N., Sáez-Gutierrez, B., Lambea, J., Tres, A., Valladares, M., and González-Fernández, Á. (2011). Assessment of the Evolution of Cancer Treatment Therapies. Cancers (Basel) *3*, 3279. https://doi.org/10.3390/CANCERS3033279.
11. Shuel, S.L. (2022). Targeted cancer therapies. Can. Fam. Physician *68*, 515–518. https://doi.org/10.46747/cfp.6807515.
12. Pucci, C., Martinelli, C., and Ciofani, G. (2019). Innovative approaches for cancer treatment: current perspectives and new challenges. ecancermedicalscience *13*, 961. https://doi.org/10.3332/ECANCER.2019.961.
13. Wyman, C., and Kanaar, R. (2006). DNA Double-Strand Break Repair: All's Well that Ends Well. Annu. Rev. Genet. *40*, 363–383. https://doi.org/10.1146/ANNUREV.GENET.40.110405.090451.
14. Ferreira da Silva, J., Salic, S., Wiedner, M., Datlinger, P., Essletzbichler, P., Hanzl, A., Superti-Furga, G., Bock, C., Winter, G., and Loizou, J.I. (2019). Genome-scale CRISPR screens are efficient in non-homologous end- joining deficient cells. Sci. Rep. *9*, 15751. https://doi.org/10.1038/s41598-019-52078-9.
15. Liao, H., Wu, J., Vandusen, N.J., Li, Y., and Zheng, Y. (2024). repair for precise gene editing. Mol. Ther. Nucleic Acid *35*, 102344. https://doi.org/10.1016/j.omtn.2024.102344.
16. Urnov, F.D., Rebar, E.J., Holmes, M.C., Zhang, H.S., and Gregory, P.D. (2010). Genome editing with engineered zinc finger nucleases. Nat. Rev. Genet. *11*, 636–646. https://doi.org/10.1038/nrg2842.
17. Moscou, M.J., and Bogdanove, A.J. (2009). A Simple Cipher Governs DNA Recognition by TAL Effectors. Science *326*, 1501. https://doi.org/10.1126/science.1178817.
18. Gaj, T., Gersbach, C.A., and Barbas, C.F. (2013). ZFN, TALEN, and CRISPR/Cas-based methods for genome engineering. Trends Biotechnol. *31*, 397–405. https://doi.org/10.1016/j.tibtech.2013.04.004.
19. Mali, P., Yang, L., Esvelt, K.M., Aach, J., Guell, M., DiCarlo, J.E., Norville, J.E., and Church, G.M. (2013). RNA-Guided Human Genome Engineering via Cas9. Science *339*, 823–826. https://doi.org/10.1126/SCIENCE.1232033.
20. Ishino, Y., Shinagawa, H., Makino, K., Amemura, M., and Nakata, A. (1987). Nucleotide sequence of the iap gene, responsible for alkaline phosphatase isozyme conversion in Escherichia coli, and identification of the gene product. J. Bacteriol. *169*, 5429. https://doi.org/10.1128/JB.169.12.5429-5433.1987.
21. Jansen, R., Embden, J.D.A. van, Gaastra, W., and Schouls, L.M. (2002). Identification of genes that are associated with DNA repeats in prokaryotes. Mol. Microbiol. *43*, 1565–1575. https://doi.org/10.1046/J.1365-2958.2002.02839.X.
22. Horvath, P., and Barrangou, R. (2010). CRISPR/Cas, the Immune System of Bacteria and Archaea. Science *327*, 167–170. https://doi.org/10.1126/SCIENCE.1179555.
23. Barrangou, R., Fremaux, C., Deveau, H., Richards, M., Boyaval, P., Moineau, S., Romero, D.A., and Horvath, P. (2007). CRISPR Provides Acquired Resistance Against Viruses in Prokaryotes. Science *315*, 1709–1712. https://doi.org/10.1126/SCIENCE.1138140.
24. Marraffini, L.A., and Sontheimer, E.J. (2008). CRISPR interference limits horizontal gene transfer in staphylococci by targeting DNA. Science *322*, 1843–1845. https://doi.org/10.1126/SCIENCE.1165771.
25. Yosef, I., Goren, M.G., and Qimron, U. (2012). Proteins and DNA elements essential for the CRISPR adaptation process in Escherichia coli. Nucleic Acids Res. *40*, 5569–5576. https://doi.org/10.1093/NAR/GKS216.
26. Wright, A.V., Nuñez, J.K., and Doudna, J.A. (2016). Biology and Applications of CRISPR Systems: Harnessing Nature's Toolbox for Genome Engineering. Cell *164*, 29–44. https://doi.org/10.1016/J.CELL.2015.12.035.
27. Hille, F., and Charpentier, E. (2016). CRISPR-Cas: biology, mechanisms and relevance. Philos. Trans. R. Soc. B Biol. Sci. *371*, 20150496. https://doi.org/10.1098/RSTB.2015.0496.
28. Lone, B.A., Karna, S.K.L., Ahmad, F., Shahi, N., and Pokharel, Y.R. (2018). CRISPR/Cas9 System: A bacterial tailor for genomic engineering. Genet. Res. Int. *2018*, 3797214. https://doi.org/10.1155/2018/3797214.
29. Shah, S.A., Erdmann, S., Mojica, F.J.M., and Garrett, R.A. (2013). Protospacer recognition motifs. RNA Biol *10*, 891–899. https://doi.org/10.4161/rna.23764.
30. Hsu, P.D., Scott, D.A., Weinstein, J.A., Ran, F.A., Konermann, S., Agarwala, V., Li, Y., Fine, E.J., Wu, X., Shalem, O., et al. (2013). DNA targeting specificity of RNA-guided Cas9 nucleases. Nat. Biotechnol. *31*, 827–832. https://doi.org/10.1038/nbt.2647.
31. Kleinstiver, B.P., Tsai, S.Q., Prew, M.S., Nguyen, N.T., Welch, M.M., Lopez, J.M., McCaw, Z.R., Aryee, M.J., and Joung, J.K. (2016). Genome-wide specificities of CRISPR-Cas Cpf1 nucleases in human cells. Nat. Biotechnol. *34*, 869–874. https://doi.org/10.1038/nbt.3620.Genome-wide.
32. Jinek, M., Chylinski, K., Fonfara, I., Hauer, M., Doudna, J.A., and Charpentier, E. (2012). A Programmable Dual-RNA–Guided DNA Endonuclease in Adaptive Bacterial Immunity. Science *337*, 816–821. https://doi.org/10.1126/SCIENCE.1225829.
33. Marraffini, L.A., and Sontheimer, E.J. (2010). CRISPR interference: RNA-directed adaptive immunity in bacteria and archaea. Nat. Rev. Genet. *11*, 181–190. https://doi.org/10.1038/nrg2749.
34. Niewoehner, O., Jinek, M., and Doudna, J.A. (2014). Evolution of CRISPR RNA recognition and processing by Cas6 endonucleases. Nucleic Acids Res. *42*, 1341–1353. https://doi.org/10.1093/NAR/GKT922.
35. Chylinski, K., Rhun, A.L., and Charpentier, E. (2013). The tracrRNA and Cas9 families of type II CRISPR-Cas immunity systems. RNA Biol. *10*, 726–737. https://doi.org/10.4161/RNA.24321.
36. Fonfara, I., Richter, H., Bratovič, M., Rhun, A.L., and Charpentier, E. (2016). The CRISPR-associated DNA-cleaving enzyme Cpf1 also processes precursor CRISPR RNA. Nature *2016*, 517–521. https://doi.org/10.1038/nature17945.

37. Jinek, M., Chylinski, K., Fonfara, I., Hauer, M., Doudna, J.A., and Charpentier, E. (2012). A programmable dual-RNA-guided DNA endonuclease in adaptive bacterial immunity. Science *337*, 816–821. https://doi.org/10.1126/science.1225829.

38. Gasiunas, G., Barrangou, R., Horvath, P., and Siksnys, V. (2012). Cas9–crRNA ribonucleoprotein complex mediates specific DNA cleavage for adaptive immunity in bacteria. Proc. Natl. Acad. Sci. *109*, E2579–E2586. https://doi.org/10.1073/PNAS.1208507109.

39. Anders, C., Niewoehner, O., Duerst, A., and Jinek, M. (2014). Structural basis of PAM-dependent target DNA recognition by the Cas9 endonuclease. Nat *2014*, 569–573. https://doi.org/10.1038/nature13579.

40. Ray, A., and Opyrchal, M. (2025). Targeting PARP1: A Promising Approach for Next-Generation Poly (ADP-ribose) Polymerase Inhibitors. Curr. Breast Cancer Rep *17*, 1–14. https://doi.org/10.1007/s12609-025-00582-5.

41. Hewish, M., Lord, C.J., Martin, S.A., Cunningham, D., and Ashworth, A. (2010). Mismatch repair deficient colorectal cancer in the era of personalized treatment. Nat. Rev. Clin. Oncol. *7*, 197–208. https://doi.org/10.1038/nrclinonc.2010.18.

42. Alaghehbandan, R., Przybycin, C.G., Verkarre, V., and Mehra, R. (2022). Chromophobe renal cell carcinoma: Novel molecular insights and clinicopathologic updates. Asian J. Urol. *9*, 1–11. https://doi.org/10.1016/j.ajur.2021.11.010.

43. Wei, S., Tamaki, A., Pei, J., Vistarop, A., McCue, P.A., Chen, L., and Flieder, D.B. (2025). NFIB::PHACTR2, a novel atypical fusion gene identified in adenoid cystic carcinoma of the palate. Virchows Arch. https://doi.org/10.1007/s00428-025-04107-4.

44. Chakravarti, A., Erkkinen, M.G., Nestler, U., Stupp, R., Mehta, M., Aldape, K., Gilbert, M.R., Black, P.M.L., and Loeffler, J.S. (2006). Temozolomide-mediated radiation enhancement in glioblastoma: A report on underlying mechanisms. Clin. Cancer Res. *12*, 4738–4746. https://doi.org/10.1158/1078-0432.CCR-06-0596.

45. Doench, J.G., Fusi, N., Sullender, M., Hegde, M., Vaimberg, E.W., Donovan, K.F., Smith, I., Tothova, Z., Wilen, C., Orchard, R., et al. (2016). Optimized sgRNA design to maximize activity and minimize off-target effects of CRISPR-Cas9. Nat. Biotechnol. *34*, 184–191. https://doi.org/10.1038/nbt.3437.

46. Giacomelli, A.O., Yang, X., Lintner, R.E., McFarland, J.M., Duby, M., Kim, J., Howard, T.P., Takeda, D.Y., Ly, S.H., Kim, E., et al. (2018). Mutational processes shape the landscape of TP53 mutations in human cancer. Nat. Genet. *50*, 1381–1387. https://doi.org/10.1038/s41588-018-0204-y.

47. Chari, R., Mali, P., Moosburner, M., and Church, G.M. (2015). Unraveling CRISPR-Cas9 genome engineering parameters via a library-on-library approach. Nat. Methods *12*, 823–826. https://doi.org/10.1038/nmeth.3473.

48. Morgens, D.W., Deans, R.M., Li, A., and Bassik, M.C. (2016). Systematic comparison of CRISPR/Cas9 and RNAi screens for essential genes. Nat. Biotechnol. *34*, 634–636. https://doi.org/10.1038/nbt.3567.

49. Koonin, E.V., and Makarova, K.S. (2019). Origins and evolution of CRISPR-Cas systems. Philos. Trans. R. Soc. B *374*, 20180087. https://doi.org/10.1098/RSTB.2018.0087.

50. Yoshimi, K., and Mashimo, T. (2022). Gene and Genome Editing Genome editing technology and applications with the type I CRISPR system. Gene Genome *3–4*, 100013. https://doi.org/10.1016/j.ggedit.2022.100013.

51. Id, M.P., Nasef, M., Chou-zheng, L., Khweis, S.A., Schoeffler, J., Hatoum-aslan, A., Stagg, S.M., and Id, J.A.D. (2023). The structure of a Type III-A CRISPR-Cas effector complex reveals conserved and idiosyncratic contacts to target RNA and crRNA among Type III-A systems. PLoS One *18*, e0287461. https://doi.org/10.1371/journal.pone.0287461.

52. Xu, Y., and Li, Z. (2020). CRISPR-Cas systems : Overview , innovations and applications in human disease research and gene therapy. Comput. Struct. Biotechnol. J. *18*, 2401–2415. https://doi.org/10.1016/j.csbj.2020.08.031.

53. Makarova, K.S., Wolf, Y.I., Alkhnbashi, O.S., Costa, F., Shah, S.A., Saunders, S.J., Barrangou, R., Brouns, S.J.J., Charpentier, E., Haft, D.H., et al. (2015). An updated evolutionary classification of CRISPR–Cas systems Kira. Nat. Rev. Microbiol. *13*, 722–736. https://doi.org/10.1038/nrmicro3569.An.

54. Summary, R. (2016). Diverse evolutionary roots and mechanistic variations of the CRISPR-Cas systems. Science *353*, aad5147. https://doi.org/10.1126/science.aad5147.

55. Lemaire, C., Gallou, B.L., Lanotte, P., Mereghetti, L., and Pastuszka, A. (2022). Distribution , Diversity and Roles of CRISPR-Cas Systems in Human and Animal Pathogenic Streptococci, *13*, pp. 1–17. https://doi.org/10.3389/fmicb.2022.828031.

56. Paul, B., and Montoya, G. (2020). CRISPR-Cas12a : Functional overview and applications. Biomed. J. *43*, 8–17. https://doi.org/10.1016/j.bj.2019.10.005.

57. Watanabe, S., Cui, B., Kiga, K., Aiba, Y., Tan, X., and Ibarra, J.A. (2019). Composition and Diversity of CRISPR-Cas13a Systems in the Genus Leptotrichia, *10*, p. 2838. https://doi.org/10.3389/fmicb.2019.02838.

58. Liu, L., Li, X., Ma, J., Li, Z., You, L., Wang, J., Wang, M., Zhang, X., and Wang, Y. (2017). The Molecular Architecture for RNA-Guided RNA Cleavage by Cas13a. Cell *170*, 714–726.e10. https://doi.org/10.1016/J.CELL.2017.06.050.

59. Nishimasu, H., Ran, F.A., Hsu, P.D., Konermann, S., Shehata, S.I., Dohmae, N., Ishitani, R., Zhang, F., and Nureki, O. (2014). Crystal Structure of Cas9 in Complex with Guide RNA and Target DNA. Cell *156*, 935–949. https://doi.org/10.1016/J.CELL.2014.02.001.

60. Jiang, F., Zhou, K., Ma, L., Gressel, S., and Doudna, J.A. (2015). A Cas9-guide RNA complex preorganized for target DNA recognition. Science *348*, 1477–1481. https://doi.org/10.1126/science.aab1452.

61. Sternberg, S.H., Redding, S., Jinek, M., Greene, E.C., Jennifer, A., Biophysics, M., Biology, C., Division, B., and Berkeley, L. (2014). DNA interrogation by the CRISPR RNA-guided endonucleas. Nature *507*, 62–67. https://doi.org/10.1038/nature13011.DNA.

62. Singh, D., Sternberg, S.H., Fei, J., Doudna, J.A., and Ha, T. (2016). Real-time observation of DNA recognition and rejection by the RNA-guided endonuclease Cas9. Nat. Commun. *7*, 1–8. https://doi.org/10.1038/ncomms12778.

63. Shrivastav, M., Haro, L.P.D., and Nickoloff, J.A. (2007). Regulation of DNA double-strand break repair pathway choice. Cell Res. *181*, 134–147. https://doi.org/10.1038/cr.2007.111.

64. Pardo, B., Gómez-González, B., and Aguilera, A. (2009). DNA Repair in Mammalian Cells. Cell. Mol. Life Sci. *66*, 1039–1056. https://doi.org/10.1007/S00018-009-8740-3.

65. Hibshman, G.N., Bravo, J.P.K., Zhang, H., Dangerfield, T.L., Finkelstein, I.J., Johnson, K.A., and Taylor, D.W. (2024). Unraveling the mechanisms of PAMless DNA interrogation by SpRY-Cas9. Nat. Commun. *15*, 1–15. https://doi.org/10.1038/s41467-024-47830-3.

66. Walton, R.T., Christie, K.A., Whittaker, M.N., and Kleinstiver, B.P. (2020). Unconstrained Genome Targeting with Near-PAMless Engineered CRISPR-Cas9 Variants, *368*, pp. 290–296. https://doi.org/10.1002/pmic.202200238.

67. Hashizume, R., Wakita, S., Sawada, H., Takebayashi, S., Kitabatake, Y., Miyagawa, Y., Hirokawa, Y.S., Imai, H., and Kurahashi, H. (2025). Trisomic rescue via allele-specific multiple chromosome cleavage using CRISPR-Cas9 in trisomy 21 cells. PNAS Nexus *4*, 1–13. https://doi.org/10.1093/pnasnexus/pgaf022.

68. Zhang, X., Tee, L.Y., Wang, X., Huang, Q., and Yang, S. (2015). Off-target Effects in CRISPR/Cas9-mediated Genome Engineering. Mol. Ther. Nucleic Acids *4*, e264. https://doi.org/10.1038/mtna.2015.37.

69. Sato, G., and Kuroda, K. (2023). Overcoming the Limitations of CRISPR-Cas9 Systems in Saccharomyces cerevisiae : Off-Target Effects , Epigenome , and Mitochondrial Editing. Microorganisms *11*, 1040. https://doi.org/10.3390/microorganisms11041040.

70. Gilbert, L.A., Horlbeck, M.A., Adamson, B., Villalta, J.E., Chen, Y., Whitehead, E. H., Guimaraes, C., Panning, B., Ploegh, H.L., Bassik, M.C., et al. (2014). Genome-Scale CRISPR-Mediated Control of Gene Repression and Activation. Cell *159*, 647–661. https://doi.org/10.1016/J.CELL.2014.09.029.

71. Qi, L.S., Larson, M.H., Gilbert, L.A., Doudna, J.A., Weissman, J.S., Arkin, A.P., and Lim, W.A. (2013). Repurposing CRISPR as an RNA-Guided Platform for Sequence-Specific Control of Gene Expression. Cell *152*, 1173–1183. https://doi.org/10.1016/J.CELL.2013.02.022.

72. Brezgin, S., Kostyusheva, A., Kostyushev, D., and Chulanov, V. (2019). Dead Cas Systems: Types, Principles, and Applications. Int. J. Mol. Sci. *20*, 6041. https://doi.org/10.3390/IJMS20236041.

73. Zheng, R., Zhang, L., Parvin, R., Su, L., Chi, J., Shi, K., and Ye, F. (2023). Progress and Perspective of CRISPR-Cas9 Technology in Translational Medicine. Adv. Sci. *10*, 1–25. https://doi.org/10.1002/advs.202300195.

74. Bikard, D., Jiang, W., Samai, P., Hochschild, A., Zhang, F., and Marraffini, L.A. (2013). Programmable repression and activation of bacterial gene expression using an engineered CRISPR-Cas system. Nucleic Acids Res. *41*, 7429–7437. https://doi.org/10.1093/NAR/GKT520.

75. Thakore, P.I., D'Ippolito, A.M., Song, L., Safi, A., Shivakumar, N.K., Kabadi, A.M., Reddy, T.E., Crawford, G.E., and Gersbach, C.A. (2015). Highly specific epigenome editing by CRISPR-Cas9 repressors for silencing of distal regulatory elements. Nat. Methods *12*, 1143–1149. https://doi.org/10.1038/nmeth.3630.

76. O'Geen, H., Bates, S.L., Carter, S.S., Nisson, K.A., Halmai, J., Fink, K.D., Rhie, S.K., Farnham, P.J., and Segal, D.J. (2019). Ezh2-dCas9 and KRAB-dCas9 enable engineering of epigenetic memory in a context-dependent manner. Epigenetics Chromatin *12*, 1–20. https://doi.org/10.1186/S13072-019-0275-8.

77. Alerasool, N., Segal, D., Lee, H., and Taipale, M. (2020). An efficient KRAB domain for CRISPRi applications in human cells. Nat. Methods *17*, 1093–1096. https://doi.org/10.1038/s41592-020-0966-x.

78. Edenhofer, F.C., Térmeg, A., Ohnuki, M., Jocher, J., Kliesmete, Z., Briem, E., Hellmann, I., and Enard, W. (2024). Generation and characterization of inducible KRAB-dCas9 iPSCs from primates for cross-species CRISPRi. iScience *27*, 110090. https://doi.org/10.1016/j.isci.2024.110090.

79. Amabile, A., Migliara, A., Capasso, P., Biffi, M., Cittaro, D., Naldini, L., and Lombardo, A. (2016). Inheritable Silencing of Endogenous Genes by Hit-and-Run Targeted Epigenetic Editing. Cell *167*, 219–232.e14. https://doi.org/10.1016/J.CELL.2016.09.006.

80. O'geen, H., Tomkova, M., Combs, J.A., Tilley, E.K., and Segal, D.J. (2022). Determinants of heritable gene silencing for KRAB-dCas9 + DNMT3 and Ezh2-dCas9 + DNMT3 hit-and-run epigenome editing. Nucleic Acids Res. *50*, 3239–3253. https://doi.org/10.1093/nar/gkac123.

81. Stepper, P., Kungulovski, G., Jurkowska, R.Z., Chandra, T., Krueger, F., Reinhardt, R., Reik, W., Jeltsch, A., and Jurkowski, T.P. (2017). Efficient targeted DNA methylation with chimeric dCas9–Dnmt3a–Dnmt3L methyltransferase. Nucleic Acids Res. *45*, 1703. https://doi.org/10.1093/NAR/GKW1112.

82. Huang, Y.-H., Su, J., Lei, Y., Brunetti, L., Gundry, M.C., Zhang, X., Jeong, M., Li, W., and Goodell, M.A. (2017). DNA epigenome editing using CRISPR-Cas SunTag-directed DNMT3A. Genome Biol. *18*, 1–11. https://doi.org/10.1186/S13059-017-1306-Z.

83. Perez-Pinera, P., Kocak, D.D., Vockley, C.M., Adler, A.F., Kabadi, A.M., Polstein, L.R., Thakore, P.I., Glass, K.A., Ousterout, D.G., Leong, K.W., et al. (2013). RNA-guided gene activation by CRISPR-Cas9–based transcription factors. Nat. Methods *10*, 973–976. https://doi.org/10.1038/nmeth.2600.

84. Hilton, I.B., D'Ippolito, A.M., Vockley, C.M., Thakore, P.I., Crawford, G.E., Reddy, T.E., and Gersbach, C.A. (2015). Epigenome editing by a CRISPR-Cas9-based acetyltransferase activates genes from promoters and enhancers. Nat. Biotechnol. *33*, 510–517. https://doi.org/10.1038/NBT.3199.

85. Tanenbaum, M.E., Gilbert, L.A., Qi, L.S., Weissman, J.S., and Vale, R.D. (2014). A protein-tagging system for signal amplification in gene expression and fluorescence imaging. Cell *159*, 635–646. https://doi.org/10.1016/J.CELL.2014.09.039.

86. Zhou, H., Liu, J., Zhou, C., Gao, N., Rao, Z., Li, H., Hu, X., Li, C., Yao, X., Shen, X., et al. (2018). In vivo simultaneous transcriptional activation of multiple genes in the brain using CRISPR-dCas9-activator transgenic mice. Nat. Neurosci. *21*, 440–446. https://doi.org/10.1038/S41593-017-0060-6.

87. Cheng, A.W., Wang, H., Yang, H., Shi, L., Katz, Y., Theunissen, T.W., Rangarajan, S., Shivalila, C.S., Dadon, D.B., and Jaenisch, R. (2013). Multiplexed activation of endogenous genes by CRISPR-on, an RNA-guided transcriptional activator system. Cell Res. *23*, 1163–1171. https://doi.org/10.1038/CR.2013.122.

88. Kunii, A., Hara, Y., Takenaga, M., Hattori, N., Fukazawa, T., Ushijima, T., Yamamoto, T., and Sakuma, T. (2018). Three-Component Repurposed Technology for Enhanced Expression: Highly Accumulable Transcriptional Activators via Branched Tag Arrays. Cris. J. *1*, 337. https://doi.org/10.1089/CRISPR.2018.0009.

89. Trevino, A.E., and Zhang, F. (2014). Genome Editing Using Cas9 Nickases, 1st ed. (Elsevier Inc.). https://doi.org/10.1016/B978-0-12-801185-0.00008-8.

90. Cong, L., Ran, F.A., Cox, D., Lin, S., Barretto, R., Habib, N., Hsu, P.D., Wu, X., Jiang, W., Marraffini, L.A., and Zhang, F. (2013). Multiplex Genome Engineering Using CRISPR/Cas Systems. Science *339*, 819–823. https://doi.org/10.1126/SCIENCE.1231143.

91. Torella, L., Klermund, J., Bilbao-arribas, M., Tamayo, I., Andrieux, G., Chmielewski, K.O., Vales, A., Olagüe, C., Moreno-luqui, D., Raimondi, I., et al. (2024). Efficient and safe therapeutic use of paired Cas9- nickases for primary hyperoxaluria type 1. EMBO Mol. Med. *16*, 112. https://doi.org/10.1038/s44321-023-00008-8.

92. Bulcaen, M., Kortleven, P., Liu, R.B., Maule, G., Dreano, E., Kelly, M., Ensinck, M. M., Thierie, S., Smits, M., Ciciani, M., et al. (2024). Prime editing functionally corrects cystic fibrosis-causing CFTR mutations in human organoids and airway epithelial cells. Cell Reports Med *5*, 101544. https://doi.org/10.1016/j.xcrm.2024.101544.

93. Chen, P.J., Hussmann, J.A., Yan, J., Knipping, F., Ravisankar, P., Chen, P.F., Chen, C., Nelson, J.W., Newby, G.A., Sahin, M., et al. (2021). Enhanced prime editing systems by manipulating cellular determinants of editing outcomes. Cell *184*, 5635–5652.e29. https://doi.org/10.1016/j.cell.2021.09.018.

94. Liu, G., Lin, Q., Jin, S., and Gao, C. (2022). The CRISPR-Cas toolbox and gene editing technologies. Mol. Cell *82*, 333–347. https://doi.org/10.1016/j.molcel.2021.12.002.

95. Komor, A.C., Kim, Y.B., Packer, M.S., Zuris, J.A., and Liu, D.R. (2016). Programmable editing of a target base in genomic DNA without double-stranded DNA cleavage. Nat *2015*, 420–424. https://doi.org/10.1038/nature17946.

96. Gaudelli, N.M., Komor, A.C., Rees, H.A., Packer, M.S., Badran, A.H., Bryson, D.I., and Liu, D.R. (2018). Programmable base editing of A·T to G·C in genomic DNA without DNA cleavage. Nature *551*, 464–471. https://doi.org/10.1038/nature24644.

97. Richter, M.F., Zhao, K.T., Eton, E., Lapinaite, A., Newby, G.A., Thuronyi, B.W., Wilson, C., Koblan, L.W., Zeng, J., Bauer, D.E., et al. (2020). Phage-assisted evolution of an adenine base editor with improved Cas domain compatibility and activity. Nat. Biotechnol. *38*, 901. https://doi.org/10.1038/s41587-020-0562-8.

98. Yin, S., Gao, L., Sun, X., Zhang, M., Gao, H., Chen, X., Zhang, D., Ming, X., Yang, L., Hu, Y., et al. (2024). Amelioration of metabolic and behavioral defects through base editing in the PahR408W phenylketonuria mouse model. Mol. Ther. *33*, 119–132. https://doi.org/10.1016/j.ymthe.2024.11.032.

99. Yeh, W.H., Chiang, H., Rees, H.A., Edge, A.S.B., and Liu, D.R. (2018). In vivo base editing of post-mitotic sensory cells. Nat. Commun. *9*, 1–10. https://doi.org/10.1038/s41467-018-04580-3.

100. Liang, P., Ding, C., Sun, H., Xie, X., Xu, Y., Zhang, X., and Sun, Y. (2017). Correction of β -thalassemia mutant by base editor in human embryos. Protein Cell *8*, 811–822. https://doi.org/10.1007/s13238-017-0475-6.

101. Maeder, M.L., Stefanidakis, M., Wilson, C.J., Baral, R., Barrera, L.A., Bounoutas, G. S., Bumcrot, D., Chao, H., Ciulla, D.M., DaSilva, J.A., et al. (2019). Development of a gene-editing approach to restore vision loss in Leber congenital amaurosis type 10. Nat. Med. *25*, 229–233. https://doi.org/10.1038/s41591-018-0327-9.

102. Musunuru, K., Grandinette, S.A., Wang, X., Hudson, T.R., Briseno, K., Berry, A.M., Hacker, J.L., Hsu, A., Silverstein, R.A., Hille, L.T., et al. (2025). Patient-Specific In Vivo Gene Editing to Treat a Rare Genetic Disease. N. Engl. J. Med. *392*, 2235–2243. https://doi.org/10.1056/NEJMoa2504747.

103. Shmakov, S., Abudayyeh, O.O., Makarova, K.S., Wolf, Y.I., Gootenberg, J.S., Semenova, E., Minakhin, L., Joung, J., Konermann, S., Severinov, K., et al. (2015). Discovery and Functional Characterization of Diverse Class 2 CRISPR-Cas Systems. Mol. Cell *60*, 385–397. https://doi.org/10.1016/J.MOLCEL.2015.10.008.

104. Zetsche, B., Gootenberg, J.S., Abudayyeh, O.O., Slaymaker, I.M., Makarova, K.S., Essletzbichler, P., Volz, S.E., Joung, J., van der Oost, J., Regev, A., et al. (2015). Cpf1 Is a Single RNA-Guided Endonuclease of a Class 2 CRISPR-Cas System. Cell *163*, 759–771. https://doi.org/10.1016/J.CELL.2015.09.038.

105. Gao, L., Cox, D.B.T., Yan, W.X., Manteiga, J.C., Schneider, M.W., Yamano, T., Nishimasu, H., Nureki, O., Crosetto, N., and Zhang, F. (2017). Engineered Cpf1

variants with altered PAM specificities. Nat. Biotechnol. *35*, 789–792. https://doi.org/10.1038/nbt.3900.

106. Anzalone, A.V., Randolph, P.B., Davis, J.R., Sousa, A.A., Koblan, L.W., Levy, J.M., Chen, P.J., Wilson, C., Newby, G.A., Raguram, A., et al. (2019). Search-and-replace genome editing without double-strand breaks or donor DNA. Nature *576*, 149–157. https://doi.org/10.1038/s41586-019-1711-4.

107. Kim, D., Kim, J., Hur, J.K., Been, K.W., Yoon, S., and Kim, J.-S. (2016). Genome-wide analysis reveals specificities of Cpf1 endonucleases in human cells. Nat. Biotechnol. *34*, 863–868. https://doi.org/10.1038/nbt.3609.

108. Zetsche, B., Heidenreich, M., Mohanraju, P., Fedorova, I., Kneppers, J., DeGennaro, E.M., Winblad, N., Choudhury, S.R., Abudayyeh, O.O., Gootenberg, J.S., et al. (2016). Multiplex gene editing by CRISPR–Cpf1 using a single crRNA array. Nat. Biotechnol. *35*, 31–34. https://doi.org/10.1038/nbt.3737.

109. Campa, C.C., Weisbach, N.R., Santinha, A.J., Incarnato, D., and Platt, R.J. (2019). Multiplexed genome engineering by Cas12a and CRISPR arrays encoded on single transcripts. Nat. Methods *16*, 887–893. https://doi.org/10.1038/s41592-019-0508-6.

110. Mahas, A., Neal Stewart, C., and Mahfouz, M.M. (2018). Harnessing CRISPR/Cas systems for programmable transcriptional and post-transcriptional regulation. Biotechnol. Adv. *36*, 295–310. https://doi.org/10.1016/J.BIOTECHADV.2017.11.008.

111. Anantharaman, V., Makarova, K.S., Burroughs, A.M., Koonin, E.V., and Aravind, L. (2013). Comprehensive analysis of the HEPN superfamily: identification of novel roles in intra-genomic conflicts, defense, pathogenesis and RNA processing. Biol. Direct *81*, 1–28. https://doi.org/10.1186/1745-6150-8-15.

112. Abudayyeh, O.O., Gootenberg, J.S., Essletzbichler, P., Han, S., Joung, J., Belanto, J. J., Verdine, V., Cox, D.B.T., Kellner, M.J., Regev, A., et al. (2017). RNA targeting with CRISPR–Cas13. Nat *2017*, 280–284. https://doi.org/10.1038/nature24049.

113. East-Seletsky, A., O'Connell, M.R., Knight, S.C., Burstein, D., Cate, J.H.D., Tjian, R., and Doudna, J.A. (2016). Two distinct RNase activities of CRISPR-C2c2 enable guide-RNA processing and RNA detection. Nat *2016*, 270–273. https://doi.org/10.1038/nature19802.

114. Smargon, A.A., Cox, D.B.T., Pyzocha, N.K., Zheng, K., Slaymaker, I.M., Gootenberg, J.S., Abudayyeh, O.A., Essletzbichler, P., Shmakov, S., Makarova, K. S., et al. (2017). Cas13b Is a Type VI-B CRISPR-Associated RNA-Guided RNase Differentially Regulated by Accessory Proteins Csx27 and Csx28. Mol. Cell *65*, 618–630.e7. https://doi.org/10.1016/J.MOLCEL.2016.12.023.

115. Cox, D.B.T., Gootenberg, J.S., Abudayyeh, O.O., Franklin, B., Kellner, M.J., Joung, J., and Zhang, F. (2017). RNA editing with CRISPR-Cas13. Science *358*, 1019–1027. https://doi.org/10.1126/SCIENCE.AAQ0180.

116. Konermann, S., Lotfy, P., Brideau, N.J., Oki, J., Shokhirev, M.N., and Hsu, P.D. (2018). Transcriptome Engineering with RNA-Targeting Type VI-D CRISPR Effectors. Cell *173*, 665–676.e14. https://doi.org/10.1016/j.cell.2018.02.033.

117. Kellner, M.J., Koob, J.G., Gootenberg, J.S., Abudayyeh, O.O., and Zhang, F. (2019). SHERLOCK: nucleic acid detection with CRISPR nucleases. Nat. Protoc. *14*, 2986–3012. https://doi.org/10.1038/s41596-019-0210-2.

118. Gootenberg, J.S., Abudayyeh, O.O., Lee, J.W., Essletzbichler, P., Dy, A.J., Joung, J., Verdine, V., Donghia, N., Daringer, N.M., Freije, C.A., et al. (2017). Nucleic acid detection with CRISPR-Cas13a/C2c2. Science *356*, 438. https://doi.org/10.1126/science.aam9321.

119. Abbott, T.R., Dhamdhere, G., Liu, Y., Lin, X., Goudy, L., Zeng, L., Chemparathy, A., Chmura, S., Heaton, N.S., Debs, R., et al. (2020). Development of CRISPR as an Antiviral Strategy to Combat SARS-CoV-2 and Influenza. Cell *181*, 865–876.e12. https://doi.org/10.1016/j.cell.2020.04.020.

120. Shalem, O., Sanjana, N.E., Hartenian, E., Shi, X., Scott, D.A., Mikkelsen, T.S., Heckl, D., Ebert, B.L., Root, D.E., Doench, J.G., et al. (2014). Genome-Scale CRISPR-Cas9 Knockout Screening in Human Cells. Science *343*, 84–87. https://doi.org/10.1126/SCIENCE.1247005.

121. Wang, T., Wei, J.J., Sabatini, D.M., and Lander, E.S. (2014). Genetic Screens in Human Cells Using the CRISPR-Cas9 System. Science *343*, 80–84. https://doi.org/10.1126/SCIENCE.1246981.

122. Luo, J. (2016). CRISPR/Cas9: From Genome Engineering to Cancer Drug Discovery. Trends Cancer *2*, 313–324. https://doi.org/10.1016/J.TRECAN.2016.05.001.

123. Hart, T., Chandrashekhar, M., Aregger, M., Steinhart, Z., Brown, K.R., MacLeod, G., Mis, M., Zimmermann, M., Fradet-Turcotte, A., Sun, S., et al. (2015). High-Resolution CRISPR Screens Reveal Fitness Genes and Genotype-Specific Cancer Liabilities. Cell *163*, 1515–1526. https://doi.org/10.1016/J.CELL.2015.11.015.

124. Wang, T., Wei, J.J., Sabatini, D.M., and Lander, E.S. (2014). Genetic screens in human cells using the CRISPR-Cas9 system. Science *343*, 80–84. https://doi.org/10.1126/SCIENCE.1246981.

125. Wang, T., Birsoy, K., Hughes, N.W., Krupczak, K.M., Post, Y., Wei, J.J., Lander, E. S., and Sabatini, D.M. (2015). Identification and characterization of essential genes in the human genome. Science *350*, 1096–1101. https://doi.org/10.1126/SCIENCE.AAC7041.

126. Konermann, S., Brigham, M.D., Trevino, A.E., Joung, J., Abudayyeh, O.O., Barcena, C., Hsu, P.D., Habib, N., Gootenberg, J.S., Nishimasu, H., et al. (2014). Genome-scale transcriptional activation by an engineered CRISPR-Cas9 complex. Nat *2014 5177536 517*, 583–588. https://doi.org/10.1038/nature14136.

127. Huarte, M. (2015). The emerging role of lncRNAs in cancer. Nat. Med. *21*, 1253–1261. https://doi.org/10.1038/nm.3981.

128. Zhu, S., Li, W., Liu, J., Chen, C.-H., Liao, Q., Xu, P., Xu, H., Xiao, T., Cao, Z., Peng, J., et al. (2016). Genome-scale deletion screening of human long non-coding RNAs using a paired-guide RNA CRISPR–Cas9 library. Nat. Biotechnol. *34*, 1279–1286. https://doi.org/10.1038/NBT.3715.

129. Cho, S.W., Xu, J., Sun, R., Mumbach, M.R., Carter, A.C., Chen, Y.G., Yost, K.E., Kim, J., He, J., Nevins, S.A., et al. (2018). Promoter of lncRNA Gene PVT1 Is a Tumor-Suppressor DNA Boundary Element. Cell *173*, 1398–1412.e22. https://doi.org/10.1016/j.cell.2018.03.068.

130. Tseng, Y.Y., Moriarity, B.S., Gong, W., Akiyama, R., Tiwari, A., Kawakami, H., Ronning, P., Reuland, B., Guenther, K., Beadnell, T.C., et al. (2014). PVT1 dependence in cancer with MYC copy-number increase. Nature *512*, 82–86. https://doi.org/10.1038/nature13311.

131. Liu, S.J., Horlbeck, M.A., Cho, S.W., Birk, H.S., Malatesta, M., He, D., Attenello, F. J., Villalta, J.E., Cho, M.Y., Chen, Y., et al. (2017). CRISPRi-based genome-scale identification of functional long non-coding RNA loci in human cells. Science *355*, aah7111. https://doi.org/10.1126/SCIENCE.AAH7111.

132. Adli, M. (2018). The CRISPR tool kit for genome editing and beyond. Nat. Commun. *9*, 1911. https://doi.org/10.1038/s41467-018-04252-2.

133. Pflueger, C., Tan, D., Swain, T., Nguyen, T., Pflueger, J., Nefzger, C., Polo, J.M., Ford, E., and Lister, R. (2018). A modular dCas9-SunTag DNMT3A epigenome editing system overcomes pervasive off-target activity of direct fusion dCas9-DNMT3A constructs. Genome Res. *28*, 1193–1206. https://doi.org/10.1101/gr.233049.117.

134. Wang, Q., Xiong, F., Wu, G., Liu, W., Chen, J., Wang, B., and Chen, Y. (2022). Gene body methylation in cancer: molecular mechanisms and clinical applications. Clin. Epigenetics *14*, 1–14. https://doi.org/10.1186/s13148-022-01382-9.

135. Pandey, V., Punniyamoorthy, S., and Pokharel, Y.R. (2023). Emerging Roles of SPT5 in Transcription. Cell. Physiol. Biochem. *57*, 395–408. https://doi.org/10.33594/000000665.

136. Shin, Y., Kim, M., Won, J., Kim, J., Oh, S.B., Lee, J.-H., and Park, K. (2020). Epigenetic Modification of CFTR in Head and Neck Cancer. J. Clin. Med. *9*, 734. https://doi.org/10.3390/jcm9030734.

137. Liu, X.S., Wu, H., Ji, X., Stelzer, Y., Wu, X., Czauderna, S., Shu, J., Dadon, D., Young, R.A., and Jaenisch, R. (2016). Editing DNA Methylation in the Mammalian Genome. Cell *167*, 233–247.e17. https://doi.org/10.1016/j.cell.2016.08.056.

138. Vojta, A., Dobrinic, P., Tadic, V., Bockor, L., Korac, P., Julg, B., Klasic, M., and Zoldos, V. (2016). Repurposing the CRISPR-Cas9 system for targeted DNA methylation. Nucleic Acids Res. *44*, 5615–5628. https://doi.org/10.1093/nar/gkw159.

139. Xie, N., Zhou, Y., Sun, Q., and Tang, B. (2018). Novel epigenetic techniques provided by the CRISPR/Cas9 System. Stem Cells Int. *2018*, 7834175. https://doi.org/10.1155/2018/7834175.

140. Anton, T., Karg, E., and Bultmann, S. (2018). Applications of the CRISPR/Cas system beyond gene editing. Biol. Methods Protoc. *3*, 1–10. https://doi.org/10.1093/biomethods/bpy002.

141. Reck, M., Rodríguez-Abreu, D., Robinson, A.G., Hui, R., Csőszi, T., Fülöp, A., Gottfried, M., Peled, N., Tafreshi, A., Cuffe, S., et al. (2016). KEYNOTE-024 Investigators. Pembrolizumab versus Chemotherapy for PD-L1-Positive Non-Small-Cell Lung Cancer. New Eng. J. *375*, 1823–1833. https://doi.org/10.1056/NEJMoa1606774.

142. Ding, X., Seebeck, T., Feng, Y., Jiang, Y., Davis, G.D., and Chen, F. (2019). Improving CRISPR-Cas9 Genome Editing Efficiency by Fusion with Chromatin-Modulating Peptides. CRISPR J. *2*, 51–63. https://doi.org/10.1089/crispr.2018.0036.

143. Li, J., Mahata, B., Escobar, M., Hilton, I.B., Goell, J., and Wang, K. (2021). Programmable human histone phosphorylation and gene activation using a CRISPR/Cas9-based chromatin kinase. Nat. Commun. *12*, 896. https://doi.org/10.1038/s41467-021-21188-2.

144. Cai, R., Lv, R., Shi, X., and Yang, G. (2023). CRISPR/dCas9 Tools : Epigenetic Mechanism and Application in Gene Transcriptional Regulation. Int. J. Mol. Sci. *24*, 14865. https://doi.org/10.3390/ijms241914865.

145. Qu, P., Yang, Y., Alameh, M., and Musunuru, K. (2023). Epigenome Editing Durability Varies Widely Across Cardiovascular Disease Target Genes. Arterioscler. Thromb. Vasc. Biol. *43*, 2075. https://doi.org/10.1161/ATVBAHA.123.319748.

146. Ruan, Y., Wang, J., Yu, M., Wang, F., Wang, J., Xu, Y., Liu, L., Cheng, Y., Yang, R., Zhang, C., et al. (2023). A multi-omics integrative analysis based on CRISPR screens re-defines the pluripotency regulatory network in ESCs. Commun. Biol. *6*, 1–17. https://doi.org/10.1038/s42003-023-04700-w.

147. Dolgalev, G., and Poverennaya, E. (2021). Applications of CRISPR-Cas technologies to proteomics. Genes *12*, 1790. https://doi.org/10.3390/genes12111790.

148. Emma, L.B., Liu, J., Phillip, J., and Robinson, R.R.R. (2022). Clinical applications of mass spectrometry-based proteomics in cancer Where are we. Proteomics *23*, 2200238. https://doi.org/10.1002/pmic.202200238.

149. Cheng, J., Lin, G., Wang, T., Wang, Y., Guo, W., Liao, J., Yang, P., Chen, J., Shao, X., Lu, X., Zhu, L., et al. (2022). Massively Parallel CRISPR-Based Genetic Perturbation Screening at Single-Cell Resolution. Adv. Sci. *10*, 2204484. https://doi.org/10.1002/advs.202204484.

150. Xu, Z., Sziraki, A., Lee, J., Zhou, W., and Cao, J. (2024). Dissecting key regulators of transcriptome kinetics through scalable single-cell RNA profiling of pooled CRISPR screens. Nat. Biotechnol. *42*, 1218–1223. https://doi.org/10.1038/s41587-023-01948-9.

151. Yao, D., Binan, L., Bezney, J., Simonton, B., Freedman, J., Frangieh, C.J., Dey, K., Geiger-Schuller, K., Eraslan, B., Gusev, A., et al. (2024). Scalable genetic screening for regulatory circuits using compressed Perturb-seq. Nat. Biotechnol. *42*, 1282–1295. https://doi.org/10.1038/s41587-023-01964-9.

152. Asmamaw Mengstie, M. (2022). Viral Vectors for the in Vivo Delivery of CRISPR Components: Advances and Challenges. Front. Bioeng. Biotechnol. *10*, 1–6. https://doi.org/10.3389/fbioe.2022.895713.

153. Xu, C.L., Ruan, M.Z.C., Mahajan, V.B., and Tsang, S.H. (2019). Viral delivery systems for crispr. Viruses *11*, 1–12. https://doi.org/10.3390/v11010028.

154. Palmgren, G. (2024). Gene-Editing Tools: Delivery Methods and Challenges. Cris. Med. News Clin. Trials. https://crisprmedicinenews.com/news/gene-editing-tools-delivery-methods-and-challenges/.

155. Wang, J.H., Gessler, D.J., Zhan, W., Gallagher, T.L., and Gao, G. (2024). Adeno-associated virus as a delivery vector for gene therapy of human diseases. Signal Transduct. Target. Ther. *9*, 78. https://doi.org/10.1038/s41392-024-01780-w.

156. Wang, D., Zhang, F., and Gao, G. (2020). CRISPR-Based Therapeutic Genome Editing: Strategies and In Vivo Delivery by AAV Vectors. Cell *181*, 136–150. https://doi.org/10.1016/j.cell.2020.03.023.

157. Bulcha, J.T., Wang, Y., Ma, H., Tai, P.W.L., and Gao, G. (2021). Viral vector platforms within the gene therapy landscape. Signal Transduct. Target. Ther. *6*, 53. https://doi.org/10.1038/s41392-021-00487-6.

158. Wang, Y., Jiang, H., Li, M., Xu, Z., Xu, H., Chen, Y., Chen, K., Zheng, W., Lin, W., Liu, Z., et al. (2024). Delivery of CRISPR/Cas9 system by AAV as vectors for gene therapy. Gene *927*, 148733. https://doi.org/10.1016/j.gene.2024.148733.

159. Himeda, C.L., Jones, T.I., and Jones, P.L. (2021). Targeted epigenetic repression by CRISPR/dSaCas9 suppresses pathogenic DUX4-fl expression in FSHD. Mol. Ther. Methods Clin. Dev. *20*, 298–311. https://doi.org/10.1016/j.omtm.2020.12.001.

160. Chehelgerdi, M., Chehelgerdi, M., Khorramian-Ghahfarokhi, M., Shafieizadeh, M., Mahmoudi, E., Eskandari, F., Rashidi, M., Arshi, A., and Mokhtari-Farsani, A. (2024). Comprehensive review of CRISPR-based gene editing: mechanisms, challenges, and applications in cancer therapy. Mol. Cancer *23*, 1–45. https://doi.org/10.1186/s12943-023-01925-5.

161. Kantor, B., O'Donovan, B., Rittiner, J., Hodgson, D., Lindner, N., Guerrero, S., Dong, W., Zhang, A., and Chiba-Falek, O. (2024). The therapeutic implications of all-in-one AAV-delivered epigenome-editing platform in neurodegenerative disorders. Nat. Commun. *15*, 1–15. https://doi.org/10.1038/s41467-024-50515-6.

162. Nguyen, N.T.K., Chang, Y.H., Truong, V.A., Hsu, M.N., Pham, N.N., Chang, C.W., Wu, Y.H., Chang, Y.H., Li, H., and Hu, Y.C. (2021). CRISPR activation of long non-coding RNA DANCR promotes bone regeneration. Biomaterials *275*, 120965. https://doi.org/10.1016/j.biomaterials.2021.120965.

163. Finn, J.D., Smith, A.R., Patel, M.C., Shaw, L., Youniss, M.R., van Heteren, J., Dirstine, T., Ciullo, C., Lescarbeau, R., Seitzer, J., et al. (2018). A Single Administration of CRISPR/Cas9 Lipid Nanoparticles Achieves Robust and Persistent In Vivo Genome Editing. Cell Rep. *22*, 2227–2235. https://doi.org/10.1016/j.celrep.2018.02.014.

164. Li, T., Yang, Y., Qi, H., Cui, W., Zhang, L., Fu, X., He, X., Liu, M., Li, P.-f., and Yu, T. (2023). CRISPR/Cas9 therapeutics: progress and prospects. Signal Transduct. Target. Ther. *8*, 36. https://doi.org/10.1038/s41392-023-01309-7.

165. Rosenblum, D., Gutkin, A., Kedmi, R., Ramishetti, S., Veiga, N., Jacobi, A.M., Schubert, M.S., Friedmann-Morvinski, D., Cohen, Z.R., Behlke, M.A., et al. (2020). CRISPR-Cas9 genome editing using targeted lipid nanoparticles for cancer therapy. Sci. Adv. *6*, 1–12. https://doi.org/10.1126/sciadv.abc9450.

166. Henderson, H. (2024). CRISPR Clinical Trials: A 2024 Update. Innov. Genomics Inst. https://innovativegenomics.org/news/crispr-clinical-trials-2024/.

167. Chen, K., Han, H., Zhao, S., Xu, B., Yin, B., Trinidad, M., Burgstone, B.W., Murthy, N., and Doudna, J.A. (2023). Lung and Liver Editing by Lipid Nanoparticle Delivery of a Stable CRISPR-Cas9 RNP (Springer). https://doi.org/10.1038/s41587-024-02437-3.

168. Mohammadian Farsani, A., Mokhtari, N., Nooraei, S., Bahrulolum, H., Akbari, A., Farsani, Z.M., Khatami, S., Ebadi, M. sadat, and Ahmadian, G. (2024). Lipid nanoparticles: The game-changer in CRISPR-Cas9 genome editing. Heliyon *10*, e24606. https://doi.org/10.1016/j.heliyon.2024.e24606.

169. Xu, X., Liu, C., Wang, Y., Koivisto, O., Zhou, J., Shu, Y., and Zhang, H. (2021). Nanotechnology-based delivery of CRISPR/Cas9 for cancer treatment. Adv. Drug Deliv. Rev. *176*, 113891. https://doi.org/10.1016/j.addr.2021.113891.

170. Beach, M.A., Nayanathara, U., Gao, Y., Zhang, C., Xiong, Y., Wang, Y., and Such, G.K. (2024). Polymeric Nanoparticles for Drug Delivery. Chem. Rev. *124*, 5505–5616. https://doi.org/10.1021/acs.chemrev.3c00705.

171. Lu, B., Lv, X., and Le, Y. (2019). Chitosan-modified PLGA nanoparticles for control-released drug delivery. Polymers *11*, 304. https://doi.org/10.3390/polym11020304.

172. Westphal, M., Maire, C.L., and Lamszus, K. (2017). EGFR as a Target for Glioblastoma Treatment: An Unfulfilled Promise. CNS Drugs *31*, 723–735. https://doi.org/10.1007/s40263-017-0456-6.

173. Hejabi, F., Abbaszadeh, M.S., Taji, S., O'Neill, A., Farjadian, F., and Doroudian, M. (2022). Nanocarriers: A novel strategy for the delivery of CRISPR/Cas systems. Front. Chem. *10*, 1–10. https://doi.org/10.3389/fchem.2022.957572.

174. Nunes, R., Valente, J.F.A., Patrício, T.M.F., and Sousa, Â. (2025). Nanoengineered innovations on DNA delivery systems for targeted cancer therapy. J. Drug Deliv. Sci. Technol. *111*, 107131. https://doi.org/10.1016/j.jddst.2025.107131.

175. Kim, H.I., Park, J., Zhu, Y., Wang, X., Han, Y., and Zhang, D. (2024). Recent advances in extracellular vesicles for therapeutic cargo delivery. Exp. Mol. Med. *56*, 836–849. https://doi.org/10.1038/s12276-024-01201-6.

176. Li, J., Wang, J., and Chen, Z. (2025). Emerging role of exosomes in cancer therapy: progress and challenges. Mol. Cancer *24*, 13. https://doi.org/10.1186/s12943-024-02215-4.

177. Wan, T., Zhong, J., Pan, Q., Zhou, T., Ping, Y., and Liu, X. (2022). Exosome-mediated delivery of Cas9 ribonucleoprotein complexes for tissue-specific gene therapy of liver diseases. Sci. Adv. *8*, 1–13. https://doi.org/10.1126/sciadv.abp9435.

178. Kim, S.M., Yang, Y., Oh, S.J., Hong, Y., Seo, M., and Jang, M. (2017). Cancer-derived exosomes as a delivery platform of CRISPR/Cas9 confer cancer cell tropism-dependent targeting. J. Control. Release *266*, 8–16. https://doi.org/10.1016/j.jconrel.2017.09.013.

179. Agosti, E., Antonietti, S., Ius, T., Fontanella, M.M., Zeppieri, M., and Panciani, P.P. (2024). A Systematic Review of Mesenchymal Stem Cell-Derived Extracellular Vesicles: A Potential Treatment for Glioblastoma. Brain Sci. *14*, 1058. https://doi.org/10.3390/brainsci14111058.

180. Ramakrishna, S., Kwaku Dad, A.B., Beloor, J., Gopalappa, R., Lee, S.K., and Kim, H. (2014). Gene disruption by cell-penetrating peptide-mediated delivery of Cas9 protein and guide RNA. Genome Res. *24*, 1020–1027. https://doi.org/10.1101/gr.171264.113.

181. Georgeous, J., AlSawaftah, N., Abuwatfa, W.H., and Husseini, G.A. (2024). Review of Gold Nanoparticles: Synthesis, Properties, Shapes, Cellular Uptake, Targeting, Release Mechanisms and Applications in Drug Delivery and Therapy. Pharmaceutics *16*, 1332. https://doi.org/10.3390/pharmaceutics16101332.

182. Wang, P., Zhang, L., Zheng, W., Cong, L., Guo, Z., Xie, Y., Wang, L., Tang, R., Feng, Q., Hamada, Y., Gonda, K., et al. (2018). Thermo-triggered Release of CRISPR-Cas9 System by Lipid-Encapsulated Gold Nanoparticles. Angew Chem. Int. Ed. Engl. *57*, 1491–1496. https://doi.org/10.1002/anie.201708689.

183. Chehelgerdi, M., Chehelgerdi, M., Allela, O.Q.B., Pecho, R.D.C., Jayasankar, N., Rao, D.P., Thamaraikani, T., Vasanthan, M., Viktor, P., Lakshmaiya, N., et al. (2023). Progressing nanotechnology to improve targeted cancer treatment: overcoming hurdles in its clinical implementation. Mol. Cancer *22*, 169. https://doi.org/10.1186/s12943-023-01865-0.

184. Duan, L., Ouyang, K., Xu, X., Xu, L., Wen, C., Zhou, X., Qin, Z., Xu, Z., Sun, W., and Liang, Y. (2021). Nanoparticle Delivery of CRISPR/Cas9 for Genome Editing. Front. Genet. *12*, 673286. https://doi.org/10.3389/fgene.2021.673286.

185. Bui, T.A., Mei, H., Sang, R., Ortega, D.G., and Deng, W. (2024). Advancements and challenges in developing in vivo CAR T cell therapies for cancer treatment. EBioMedicine *106*, 105266. https://doi.org/10.1016/j.ebiom.2024.105266.

186. Zeng, G., Yu, Y., Wang, M., Liu, J., He, G., Yu, S., Yan, H., Yang, L., Li, H., and Peng, X. (2024). Advancing cancer research through organoid technology. J. Transl. Med. *22*, 1007. https://doi.org/10.1186/s12967-024-05824-1.

187. Geurts, M.H., and Clevers, H. (2023). CRISPR engineering in organoids for gene repair and disease modelling. Nat. Rev. Bioeng *1*, 32–45. https://doi.org/10.1038/s44222-022-00013-5.

188. Thorel, L., Perréard, M., Florent, R., Divoux, J., Coffy, S., Vincent, A., Gaggioli, C., Guasch, G., Gidrol, X., Weiswald, L.B., et al. (2024). Patient-derived tumor organoids: a new avenue for preclinical research and precision medicine in oncology. Exp. Mol. Med. *56*, 1531–1551. https://doi.org/10.1038/s12276-024-01272-5.

189. Roh, J.I., Lee, J., Park, S.U., Kang, Y.S., Lee, J., Oh, A.R., Choi, D.J., Cha, J.Y., and Lee, H.W. (2018). CRISPR-Cas9-mediated generation of obese and diabetic mouse models. Exp. Anim. *67*, 229–237. https://doi.org/10.1538/expanim.17-0123.

190. Macarrón Palacios, A., Korus, P., Wilkens, B.G.C., Heshmatpour, N., and Patnaik, S.R. (2024). Revolutionizing in vivo therapy with CRISPR/Cas genome editing: breakthroughs, opportunities and challenges. Front. Genome Ed. *6*, 1–22. https://doi.org/10.3389/fgeed.2024.1342193.

191. Malihe, L., Alexandra, E., and Butler, A.S. (2023). Application of CRISPR-Cas9 technology in diabetes research. Diabet. Med. *41*, 15240. https://doi.org/10.1111/dme.15240.

192. Drost, J., Van Boxtel, R., Blokzijl, F., Mizutani, T., Sasaki, N., Sasselli, V., De Ligt, J., Behjati, S., Grolleman, J.E., Van Wezel, T., et al. (2017). Use of CRISPR-modified human stem cell organoids to study the origin of mutational signatures in cancer. Science (Washington, D.C.) *358*, 234–238. https://doi.org/10.1126/science.aao3130.

193. Matano, M., Date, S., Shimokawa, M., Takano, A., Fujii, M., Ohta, Y., Watanabe, T., Kanai, T., and Sato, T. (2015). Modeling colorectal cancer using CRISPR-Cas9-mediated engineering of human intestinal organoids. Nat. Med. *21*, 256–262. https://doi.org/10.1038/nm.3802.

194. Ravichandran, M., and Maddalo, D. (2023). Applications of CRISPR-Cas9 for advancing precision medicine in oncology : from target discovery to disease modeling. Front. Genet. *14*, 1273994. https://doi.org/10.3389/fgene.2023.1273994.

195. Chen, S., Sanjana, N.E., Zheng, K., Shalem, O., Lee, K., Shi, X., Scott, D.A., Song, J., Pan, J.Q., Weissleder, R., et al. (2015). Genome-wide CRISPR screen in a mouse model of tumor growth and metastasis. Cell *160*, 1246–1260. https://doi.org/10.1016/j.cell.2015.02.038.

196. Song, C.-Q., Li, Y., Mou, H., Moore, J., Park, A., Pomyen, Y., Hough, S., Kennedy, Z., Fischer, A., Yin, H., et al. (2017). Genome-Wide CRISPR Screen Identifies Regulators of Mitogen-Activated Protein Kinase as Suppressors of Liver Tumors in Mice. Gastroenterology *152*, 1161–1173.e1. https://doi.org/10.1053/J.GASTRO.2016.12.002.

197. Xue, W., Chen, S., Yin, H., Tammela, T., Papagiannakopoulos, T., Joshi, N.S., Cai, W., Yang, G., Bronson, R., Crowley, D.G., et al. (2014). CRISPR-mediated direct mutation of cancer genes in the mouse liver. Nat *2014*, 380–384. https://doi.org/10.1038/nature13589.

198. Weber, J., Öllinger, R., Friedrich, M., Ehmer, U., Barenboim, M., Steiger, K., Heid, I., Mueller, S., Maresch, R., Engleitner, T., et al. (2015). CRISPR/Cas9 somatic multiplex-mutagenesis for high-throughput functional cancer genomics in mice. Proc. Natl. Acad. Sci. *112*, 13982–13987. https://doi.org/10.1073/PNAS.1512392112.

199. Li, X., Wang, Z., Man, X., Dai, X., Zhou, Q., and Zhang, S. (2025). Gynecologic Oncology Research advances CRISPR gene editing technology generated models in the study of epithelial ovarian carcinoma. Gynecol. Oncol. *195*, 34–44. https://doi.org/10.1016/j.ygyno.2025.02.022.

200. Shalem, O., Sanjana, N.E., Hartenian, E., Shi, X., Scott, D.A., Mikkelsen, T.S., Heckl, D., Ebert, B.L., Root, D.E., Doench, J.G., et al. (2014). Genome-scale CRISPR-Cas9 knockout screening in human cells. Science *343*, 84–87. https://doi.org/10.1126/SCIENCE.1247005.

201. Kumaran, A., Jude Serpes, N., Gupta, T., James, A., Sharma, A., Kumar, D., Nagraik, R., Kumar, V., and Pandey, S. (2023). Advancements in CRISPR-Based Biosensing for Next-Gen Point of Care Diagnostic Application. Biosensors *13*, 202. https://doi.org/10.3390/bios13020202.

202. Ghouneimy, A., Mahas, A., Marsic, T., Aman, R., and Mahfouz, M. (2023). CRISPR-Based Diagnostics: Challenges and Potential Solutions toward Point-of-Care Applications. ACS Synth. Biol. *12*, 1–16. https://doi.org/10.1021/acssynbio.2c00496.

203. Hassan, Y.M., Mohamed, A.S., Hassan, Y.M., and El-Sayed, W.M. (2025). Recent developments and future directions in point-of-care next-generation CRISPR-based rapid diagnosis. Clin. Exp. Med. *25*, 33. https://doi.org/10.1007/s10238-024-01540-8.

204. Chen, J.S., Ma, E., Harrington, L.B., Da Costa, M., Tian, X., Palefsky, J.M., and Doudna, J.A. (2018). CRISPR-Cas12a target binding unleashes indiscriminate single-stranded DNase activity. Science *360*, 436–439. https://doi.org/10.1126/SCIENCE.AAR6245.

205. Wirth, T., Parker, N., and Ylä-Herttuala, S. (2013). History of gene therapy. Gene *525*, 162–169. https://doi.org/10.1016/J.GENE.2013.03.137.

206. Zhan, T., Rindtor, N., Betge, J., and Ebert, M.P. (2019). Seminars in Cancer Biology CRISPR/Cas9 for cancer research and therapy. Semin. Cancer Biol. *55*, 106. https://doi.org/10.1016/j.semcancer.2018.04.001.

207. Eyquem, J., Mansilla-Soto, J., Giavridis, T., Van Der Stegen, S.J.C., Hamieh, M., Cunanan, K.M., Odak, A., Gönen, M., and Sadelain, M. (2017). Targeting a CAR to the TRAC locus with CRISPR/Cas9 enhances tumour rejection. Nature *543*, 113–117. https://doi.org/10.1038/nature21405.

208. Tebas, P., Stein, D., Tang, W.W., Frank, I., Wang, S.Q., Lee, G., Spratt, S.K., Surosky, R.T., Giedlin, M.A., Nichol, G., et al. (2014). Gene Editing of CCR5 in Autologous CD4 T Cells of Persons Infected with HIV. N. Engl. J. Med. *370*, 901–910. https://doi.org/10.1056/NEJMOA1300662.

209. Tian, Y., Li, Y., Shao, Y., and Zhang, Y. (2020). Gene modification strategies for next-generation CAR T cells against solid cancers. J. Hematol. Oncol. *13*, 1–16. https://doi.org/10.1186/s13045-020-00890-6.

210. Cas, C., Eyquem, J., Mansilla-soto, J., Giavridis, T., Stegen, S.J.C.V.D., Hamieh, M., Cunanan, K.M., Odak, A., Gönen, M., and Sadelain, M. (2017). Targeting a CAR to the TRAC locus with CRISPR/Cas9 enhances tumour rejection. Nature *543*, 113–117. https://doi.org/10.1038/nature21405.

211. Stadtmauer, E.A., Fraietta, J.A., Davis, M.M., Cohen, A.D., Weber, K.L., Lancaster, E., Mangan, P.A., Kulikovskaya, I., Gupta, M., Chen, F., et al. (2020). CRISPR-engineered T cells in patients with refractory cancer. Science *367*, 1–20. https://doi.org/10.1126/science.aba7365.

212. Choi, B.D., Yu, X., Castano, A.P., Darr, H., Henderson, D.B., Bouffard, A.A., Larson, R.C., Scarfò, I., Bailey, S.R., Gerhard, G.M., et al. (2019). CRISPR-Cas9 disruption of PD-1 enhances activity of universal EGFRvIII CAR T cells in a preclinical model of human glioblastoma. J. Immunother. Cancer *7*, 1–8. https://doi.org/10.1186/s40425-019-0806-7.

213. Wang, Z., Li, N., Feng, K., Chen, M., Zhang, Y., Liu, Y., Yang, Q., Nie, J., Tang, N., Zhang, X., et al. (2021). Phase I study of CAR-T cells with PD-1 and TCR disruption in mesothelin-positive solid tumors. Cell. Mol. Immunol. *18*, 2188–2198. https://doi.org/10.1038/s41423-021-00749-x.

214. Liu, X., Ranganathan, R., Jiang, S., Fang, C., Sun, J., Kim, S., Newick, K., Lo, A., June, C.H., Zhao, Y., et al. (2016). A chimeric switch-receptor targeting PD1 augments the efficacy of second-generation CAR T cells in advanced solid tumors. Cancer Res. *76*, 1578–1590. https://doi.org/10.1158/0008-5472.CAN-15-2524.

215. Guo, Y., Tong, C., Su, L., Zhang, W., Jia, H., Liu, Y., Yang, Q., Wu, Z., Wang, Y., and Han, W. (2022). CRISPR/Cas9 genome-edited universal CAR T cells in patients with relapsed/refractory lymphoma. Blood Adv. *6*, 2695–2699. https://doi.org/10.1182/bloodadvances.2021006232.

216. Witkowsky, L., Norstad, M., Glynn, A.R., and Kliegman, M. (2023). Towards affordable CRISPR genomic therapies: a task force convened by the Innovative Genomics Institute. Gene Ther. *30*, 747–752. https://doi.org/10.1038/s41434-023-00392-3.

217. Caforio, M., Iacovelli, S., Quintarelli, C., Locatelli, F., and Folgiero, V. (2024). GMP-manufactured CRISPR/Cas9 technology as an advantageous tool to support cancer immunotherapy. J. Exp. Clin. Cancer Res. *43*, 1–12. https://doi.org/10.1186/s13046-024-02993-1.

218. Lim, S.R., and Lee, S.J. (2024). Multiplex CRISPR-Cas Genome Editing: Next-Generation Microbial Strain Engineering. J. Agric. Food Chem. *72*, 11871–11884. https://doi.org/10.1021/acs.jafc.4c01650.

219. Sara Reardon (2024). MEGA-CRISPR tool gives a power boost to cancer-fighting cells. Nature *626*, 940. https://doi.org/10.1038/d41586-024-00511-z.

220. Roth, T.L., Puig-Saus, C., Yu, R., Shifrut, E., Carnevale, J., Li, P.J., Hiatt, J., Saco, J., Krystofinski, P., Li, H., et al. (2018). Reprogramming human T cell function and specificity with non-viral genome targeting. Nature *559*, 405–409. https://doi.org/10.1038/s41586-018-0326-5.

221. Haghighi, E., Abolmaali, S.S., Dehshahri, A., Ali, S., Shaegh, M., Azarpira, N., and Tamaddon, A.M. (2024). Navigating the intricate in - vivo journey of lipid nanoparticles tailored for the targeted delivery of RNA therapeutics : a quality - by - design approach. J. Nanobiotechnology *22*, 710. https://doi.org/10.1186/s12951-024-02972-w.

222. Qasim, W., Zhan, H., Samarasinghe, S., Adams, S., Amrolia, P., Stafford, S., Butler, K., Rivat, C., Wright, G., Somana, K., et al. (2017). Molecular remission of infant B-ALL after infusion of universal TALEN gene-edited CAR T cells. Sci. Transl. Med. *9*, 1–8. https://doi.org/10.1126/scitranslmed.aaj2013.

223. Hong, M., Clubb, J.D., and Chen, Y.Y. (2020). Engineering CAR-T Cells for Next-Generation Cancer Therapy. Cancer Cell *38*, 473–488. https://doi.org/10.1016/j.ccell.2020.07.005.

224. Li, Y.R., Zhu, Y., Fang, Y., Lyu, Z., and Yang, L. (2025). Emerging trends in clinical allogeneic CAR cell therapy. Med *6*, 100677. https://doi.org/10.1016/j.medj.2025.100677.

225. U.S. Food and Drug Administration (2023). U.S. Food and Drug Administration FDA Approves First Gene Therapies to Treat Patients with Sickle Cell Disease. https://www.fda.gov/news-events/press-announcements/fda-approves-first-gene-therapies-treat-patients-sickle-cell-disease.

226. CRISPR Medicine News Clinical Trials (2025). Gene-editing Clinical Trials. https://crisprmedicinenews.com/clinical-trials/.

227. Cetin, B., Erendor, F., Eksi, Y.E., Sanlioglu, A.D., and Sanlioglu, S. (2025). Advancing CRISPR Genome Editing into Gene Therapy Clinical Trials: Progress and Future Prospects. Expert Rev. Mol. Med. *27*, e16. https://doi.org/10.1017/erm.2025.10.

228. Tsai, S.Q., and Joung, J.K. (2016). Defining and improving the genome-wide specificities of CRISPR-Cas9 nucleases. Nat. Rev. Genet. *17*, 300–312. https://doi.org/10.1038/nrg.2016.28.

229. Fu, Y., Foden, J.A., Khayter, C., Maeder, M.L., Reyon, D., Joung, J.K., and Sander, J. D. (2013). High-frequency off-target mutagenesis induced by CRISPR-Cas nucleases in human cells. Nat. Biotechnol. *31*, 822–826. https://doi.org/10.1038/nbt.2623.

230. Slaymaker, I.M., Gao, L., Zetsche, B., Scott, D.A., Yan, W.X., and Zhang, F. (2016). Rationally engineered Cas9 nucleases with improved specificity. Science *351*, 84–88. https://doi.org/10.1126/science.aad5227.

231. Mengstie, M.A., and Wondimu, B.Z. (2021). Mechanism and applications of crispr/cas-9-mediated genome editing. Biol. Targets & Ther. *15*, 353–361. https://doi.org/10.2147/BTT.S326422.

232. Senturk, S., Shirole, N.H., Nowak, D.G., Corbo, V., Pal, D., Vaughan, A., Tuveson, D.A., Trotman, L.C., Kinney, J.B., and Sordella, R. (2017). Rapid and tunable method to temporally control gene editing based on conditional Cas9 stabilization. Nat. Commun. *8*, 1–10. https://doi.org/10.1038/ncomms14370.

233. Shirley, J.L., de Jong, Y.P., Terhorst, C., and Herzog, R.W. (2020). Immune Responses to Viral Gene Therapy Vectors. Mol. Ther. *28*, 709–722. https://doi.org/10.1016/j.ymthe.2020.01.001.

234. Lino, C.A., Harper, J.C., Carney, J.P., and Timlin, J.A. (2018). Delivering crispr: A review of the challenges and approaches. Drug Deliv. *25*, 1234–1257. https://doi.org/10.1080/10717544.2018.1474964.

235. Lôbo, G.C.N.B., Paiva, K.L.R., Silva, A.L.G., Simões, M.M., Báo, S.N., and Radicchi, M.A. (2021). Nanocarriers used in drug delivery to enhance immune system in cancer therapy. Pharmaceutics *13*, 1167. https://doi.org/10.3390/pharmaceutics13081167.

236. Charlesworth, C.T., Deshpande, P.S., Dever, D.P., Camarena, J., Lemgart, V.T., Cromer, M.K., Vakulskas, C.A., Collingwood, M.A., Zhang, L., Bode, N.M., et al. (2019). Identification of preexisting adaptive immunity to Cas9 proteins in humans. Nat. Med. *25*, 249–254. https://doi.org/10.1038/s41591-018-0326-x.

237. Wagner, J., Wickman, E., DeRenzo, C., and Gottschalk, S. (2020). CAR T Cell Therapy for Solid Tumors: Bright Future or Dark Reality? Mol. Ther. *28*, 2320–2339. https://doi.org/10.1016/j.ymthe.2020.09.015.

238. Maruyama, T., Dougan, S.K., Truttmann, M.C., Bilate, A.M., Ingram, J.R., and Ploegh, H.L. (2015). Increasing the efficiency of precise genome editing with CRISPR-Cas9 by inhibition of nonhomologous end joining. Nat. Biotechnol. *33*, 538–542. https://doi.org/10.1038/nbt.3190.

239. Mehravar, M., Shirazi, A., Nazari, M., and Banan, M. (2019). Mosaicism in CRISPR/Cas9-mediated genome editing. Dev. Biol. *445*, 156–162. https://doi.org/10.1016/j.ydbio.2018.10.008.

240. Kosicki, M., Tomberg, K., and Bradley, A. (2018). Repair of double-strand breaks induced by CRISPR–Cas9 leads to large deletions and complex rearrangements. Nat. Biotechnol. *36*, 765–771. https://doi.org/10.1038/nbt.4192.

241. Yoo, K.W., Yadav, M.K., Song, Q., Atala, A., and Lu, B. (2022). Targeting DNA polymerase to DNA double-strand breaks reduces DNA deletion size and increases templated insertions generated by CRISPR/Cas9. Nucleic Acids Res. *50*, 3944–3957. https://doi.org/10.1093/nar/gkac186.

242. Elangkovan, N., and Dickson, G. (2021). Gene Therapy for Duchenne Muscular Dystrophy. J. Neuromuscul. Dis. *8*, S303–S316. https://doi.org/10.3233/JND-210678.

243. Haapaniemi, E., Botla, S., Persson, J., Schmierer, B., and Taipale, J. (2018). CRISPR – Cas9 genome editing induces a p53- mediated DNA damage response. Nat. Med. *24*, 927. https://doi.org/10.1038/s41591-018-0049-z.

244. Leibowitz, M.L., Papathanasiou, S., Doerfler, P.A., Logan, J., Sun, L., Yao, Y., Zhang, C., and Weiss, M.J. (2021). Chromothripsis as an on-target consequence of CRISPR-Cas9 genome editing. zenodo *53*, 895–905. https://doi.org/10.5281/zenodo.4533300.

245. (2023). Reuters Vertex/CRISPR price sickle cell disease gene therapy at $2.2 mln. https://www.reuters.com/business/healthcare-pharmaceuticals/vertexcrispr-price-sickle-cell-disease-gene-therapy-22-mln-2023-12-08/.

246. Conley, J.M., Davis, A.M., Henderson, G.E., Juengst, E.T., Meagher, K.M., Walker, R.L., Waltz, M., and Cadigan, J. (2020). A New Governance Approach to Regulating Human Genome Editing. North Carolina J. Law Technol. *22*, 107–141.

247. UNESCO (2023). UNESCO Two UNESCO debates on ethics of Artificial Intelligence and Gene Editing. https://www.unesco.org/en/articles/two-unesco-debates-ethics-artificial-intelligence-and-gene-editing-11-12-september.

OMTN, Volume *36*

Supplemental information

Exploring CRISPR-Cas: The transformative impact of gene editing in molecular biology

Vivek Pandey, Shivani Sharma, and Yuba Raj Pokharel

Tables:

Table S1: Cancer-Lethal Genes by CRISPR Screen Type: Loss-of-Function and Gain-of-Function

Sr. No.	Gene	Function/Pathway	Cancer Type	Screen Outcome	Therapeutic Implication
1	BCR-ABL [1]	Tyrosine kinase fusion	Chronic myeloid leukemia	Essential - LOF causes cell death	Direct target for imatinib therapy
2	KRAS [2]	Small GTPase/MAPK signaling	Colorectal, pancreatic cancer	Essential - LOF inhibits growth	Synthetic lethal targets identified
3	Anti-proliferative genes [3]	Growth suppression	K562 leukemia	GOF - activation inhibits growth	Reactivation therapy strategies
4	MAPK pathway genes [4]	Growth signaling	A375 melanoma	GOF - activation confers drug resistance	Combination MAPK inhibition
5	lncRNAs (499 identified) [5]	Gene regulation	Multiple cell lines	Cell-specific effects	lncRNA-targeted therapies
6	PVT1 [6]	Pro-growth lncRNA	Various cancer cells	Silencing - reduces growth	lncRNA modulation
7	CDK6 [2]	Etoposide	HL60 leukemia	LOF - alters cell cycle response	CDK inhibitors
8	NF2 [7]	BRAF inhibitors	A375 melanoma	LOF - activates alternative pathways	Hippo pathway targeting
9	TRIM37 [8]	PLK4 inhibitors	Multiple cancers	LOF - synthetic lethality	Biomarker-driven therapy
10	ARID1A [9]	Chromatin remodeling	LOF	Enhances T cell persistence	Epigenetic modulators
11	SLC7A1/SLC38A2 [10]	Amino acid transport	LOF	Affects T cell metabolism	Metabolic modulators

Table S2: CRISPR Clinical Trials - Therapeutic Potential and Global Progress

Sr. No.	Trial/Therapy	CRISPR Technique	Disease Target	Country	Phase	Sample Size	Primary Outcome	Status/Results
1	CTX001 (Casgevy) [11]	Base editing (BCL11A)	Sickle cell disease, β-thalassemia	USA, Europe, Canada	III	75 patients	Transfusion independence	FDA/EMA Approved 2023 95% success
2	EDIT-101[12]	Cas9 knockout (CEP290)	Leber congenital amaurosis	USA	I/II	18 patients	Vision restoration	Ongoing - promising early results
3	CTX110 [13]	Cas9 (TRAC knockout)	B-cell malignancies	USA, Europe	I	37 patients	CAR-T efficacy	70% overall response rate
4	NTLA-2001 [14]	*In vivo* base editing	Hereditary ATTR amyloidosis	USA, UK	I	12 patients	TTR protein reduction	87% reduction achieved
5	Universal CAR-T [15]	Cas9 multiplex editing	ALL, lymphoma	UK, USA	I	28 patients	Remission rates	60% complete remission
6	CRISPR-HIV (CCR5)[16,17]	Cas9 knockout	HIV infection	China, USA	I	24 patients	Viral suppression	Mixed results, ongoing studies

7	AGN-151587 [18]	Cas9 editing	Geographic atrophy (AMD)	USA	I/II	79 patients	Vision preservation	Recruiting patients
8	CRISPR-001 [11]	*Ex vivo* editing	β-thalassemia	Multiple	I/II	42 patients	Hemoglobin increase	89% transfusion reduction
9	NY-ESO-1 TCR [19]	Cas9 multiplex	Solid tumors	USA	I	20 patients	Tumor response	50% stable disease
10	Base Editor DMD [20]	Cytosine base editing	Duchenne muscular dystrophy	USA	Preclinical	N/A	Dystrophin restoration	In development

References:

(1) Hart, T.; Chandrashekhar, M.; Aregger, M.; Steinhart, Z.; Brown, K. R.; MacLeod, G.; Mis, M.; Zimmermann, M.; Fradet-Turcotte, A.; Sun, S.; Mero, P.; Dirks, P.; Sidhu, S.; Roth, F. P.; Rissland, O. S.; Durocher, D.; Angers, S.; Moffat, J. High-Resolution CRISPR Screens Reveal Fitness Genes and Genotype-Specific Cancer Liabilities. *Cell* **2015**, *163* (6), 1515–1526. https://doi.org/10.1016/J.CELL.2015.11.015.

(2) Wang, T.; Wei, J. J.; Sabatini, D. M.; Lander, E. S. Genetic Screens in Human Cells Using the CRISPR-Cas9 System. *Science (80-.).* **2014**, *343* (6166), 80–84. https://doi.org/10.1126/SCIENCE.1246981.

(3) Gilbert, L. A.; Horlbeck, M. A.; Adamson, B.; Villalta, J. E.; Chen, Y.; Whitehead, E. H.; Guimaraes, C.; Panning, B.; Ploegh, H. L.; Bassik, M. C.; Qi, L. S.; Kampmann, M.; Weissman, J. S. Genome-Scale CRISPR-Mediated Control of Gene Repression and Activation. *Cell* **2014**,

159 (3), 647–661. https://doi.org/10.1016/J.CELL.2014.09.029.

(4) Konermann, S.; Brigham, M. D.; Trevino, A. E.; Joung, J.; Abudayyeh, O. O.; Barcena, C.; Hsu, P. D.; Habib, N.; Gootenberg, J. S.; Nishimasu, H.; Nureki, O.; Zhang, F. Genome-Scale Transcriptional Activation by an Engineered CRISPR-Cas9 Complex. *Nat. 2014 5177536* **2014**, *517* (7536), 583–588. https://doi.org/10.1038/nature14136.

(5) Liu, S. J.; Horlbeck, M. A.; Cho, S. W.; Birk, H. S.; Malatesta, M.; He, D.; Attenello, F. J.; Villalta, J. E.; Cho, M. Y.; Chen, Y.; Mandegar, M. A.; Olvera, M. P.; Gilbert, L. A.; Conklin, B. R.; Chang, H. Y.; Weissman, J. S.; Lim, D. A. CRISPRi-Based Genome-Scale Identification of Functional Long Non-Coding RNA Loci in Human Cells. *Science* **2017**, *355* (6320). https://doi.org/10.1126/SCIENCE.AAH7111.

(6) Liu, S. J.; Horlbeck, M. A.; Cho, S. W.; Birk, H. S.; Malatesta, M.; He, D.; Attenello, F. J.; Villalta, J. E.; Cho, M. Y.; Chen, Y.; Mandegar, M. A.; Olvera, M. P.; Gilbert, L. A.; Conklin, B. R.; Chang, H. Y.; Weissman, J. S.; Lim, D. A. CRISPRi-Based Genome-Scale Identification of Functional Long Non-Coding RNA Loci in Human Cells HHS Public Access LncRNA Knockdown Can Perturb Complex Transcriptional Networks in a Cell Type-Specific Manner. These Data Underscore the Functional Importance. *Sci. January* **2017**, *06* (3556320), 1–19. https://doi.org/10.1126/science.aah7111.CRISPRi-based.

(7) Shalem, O.; Sanjana, N. E.; Hartenian, E.; Shi, X.; Scott, D. A.; Mikkelsen, T. S.; Heckl, D.; Ebert, B. L.; Root, D. E.; Doench, J. G.; Zhang, F. Genome-Scale CRISPR-Cas9 Knockout Screening in Human Cells. *Science (80-.).* **2014**, *343* (6166), 84–87. https://doi.org/10.1126/SCIENCE.1247005.

(8) Meitinger, F.; Ohta, M.; Lee, K. Y.; Watanabe, S.; Davis, R. L.; Anzola, J. V.; Kabeche, R.; Jenkins, D. A.; Shiau, A. K.; Desai, A.; Oegema, K. TRIM37 Controls Cancer-Specific Vulnerability to PLK4 Inhibition. *Nature* **2020**, *585* (7825), 440–446. https://doi.org/10.1038/s41586-020-2710-1.

(9) Belk, J. A.; Yao, W.; Ly, N.; Freitas, K. A.; Chen, Y. T.; Shi, Q.; Valencia, A. M.; Shifrut, E.; Kale, N.; Yost, K. E.; Duffy, C. V.; Daniel, B.; Hwee, M. A.; Miao, Z.; Ashworth, A.; Mackall, C. L.; Marson, A.; Carnevale, J.; Vardhana, S. A.; Satpathy, A. T. Genome-Wide CRISPR Screens of T Cell Exhaustion Identify Chromatin Remodeling Factors That Limit T Cell Persistence. *Cancer Cell* **2022**, *40* (7), 768-786.e7. https://doi.org/10.1016/j.ccell.2022.06.001.

(10) Huang, H.; Zhou, P.; Wei, J.; Long, L.; Shi, H.; Dhungana, Y.; Chapman, N. M.; Fu, G.; Saravia, J.; Raynor, J. L.; Liu, S.; Palacios, G.; Wang, Y. D.; Qian, C.; Yu, J.; Chi, H. In Vivo CRISPR Screening Reveals Nutrient Signaling Processes Underpinning CD8+ T Cell Fate Decisions. *Cell* **2021**, *184* (5), 1245-1261.e21. https://doi.org/10.1016/j.cell.2021.02.021.

(11) Frangoul, H.; Altshuler, D.; Cappellini, M. D.; Chen, Y.-S.; Domm, J.; Eustace, B. K.; Foell, J.; de la Fuente, J.; Grupp, S.; Handgretinger, R.; Ho, T. W.; Kattamis, A.; Kernytsky, A.; Lekstrom-Himes, J.; Li, A. M.; Locatelli, F.; Mapara, M. Y.; de Montalembert, M.; Rondelli, D.; Sharma, A.; Sheth, S.; Soni, S.; Steinberg, M. H.; Wall, D.; Yen, A.; Corbacioglu, S. CRISPR-Cas9 Gene Editing for Sickle Cell Disease and β-Thalassemia. *N. Engl. J. Med.* **2021**, *384* (3), 252–260. https://doi.org/10.1056/nejmoa2031054.

(12) Maeder, M. L.; Stefanidakis, M.; Wilson, C. J.; Baral, R.; Barrera, L. A.; Bounoutas, G. S.; Bumcrot, D.; Chao, H.; Ciulla, D. M.; DaSilva,

J. A.; Dass, A.; Dhanapal, V.; Fennell, T. J.; Friedland, A. E.; Giannoukos, G.; Gloskowski, S. W.; Glucksmann, A.; Gotta, G. M.; Jayaram, H.; Haskett, S. J.; Hopkins, B.; Horng, J. E.; Joshi, S.; Marco, E.; Mepani, R.; Reyon, D.; Ta, T.; Tabbaa, D. G.; Samuelsson, S. J.; Shen, S.; Skor, M. N.; Stetkiewicz, P.; Wang, T.; Yudkoff, C.; Myer, V. E.; Albright, C. F.; Jiang, H. Development of a Gene-Editing Approach to Restore Vision Loss in Leber Congenital Amaurosis Type 10. *Nat. Med.* **2019**, *25* (2), 229–233. https://doi.org/10.1038/s41591-018-0327-9.

(13) Stadtmauer, E. A.; Fraietta, J. A.; Davis, M. M.; Cohen, A. D.; Weber, K. L.; Lancaster, E.; Mangan, P. A.; Kulikovskaya, I.; Gupta, M.; Chen, F.; Tian, L.; Gonzalez, V. E.; Xu, J.; Jung, I. young; Joseph Melenhorst, J.; Plesa, G.; Shea, J.; Matlawski, T.; Cervini, A.; Gaymon, A. L.; Desjardins, S.; Lamontagne, A.; Salas-Mckee, J.; Fesnak, A.; Siegel, D. L.; Levine, B. L.; Jadlowsky, J. K.; Young, R. M.; Chew, A.; Hwang, W. T.; Hexner, E. O.; Carreno, B. M.; Nobles, C. L.; Bushman, F. D.; Parker, K. R.; Qi, Y.; Satpathy, A. T.; Chang, H. Y.; Zhao, Y.; Lacey, S. F.; June, C. H. CRISPR-Engineered T Cells in Patients with Refractory Cancer. *Science (80-.).* **2020**, *367* (6481), 1–20. https://doi.org/10.1126/science.aba7365.

(14) Gillmore, J. D.; Gane, E.; Taubel, J.; Kao, J.; Fontana, M.; Maitland, M. L.; Seitzer, J.; O'Connell, D.; Walsh, K. R.; Wood, K.; Phillips, J.; Xu, Y.; Amaral, A.; Boyd, A. P.; Cehelsky, J. E.; McKee, M. D.; Schiermeier, A.; Harari, O.; Murphy, A.; Kyratsous, C. A.; Zambrowicz, B.; Soltys, R.; Gutstein, D. E.; Leonard, J.; Sepp-Lorenzino, L.; Lebwohl, D. CRISPR-Cas9 In Vivo Gene Editing for Transthyretin Amyloidosis. *N. Engl. J. Med.* **2021**, *385* (6), 493–502. https://doi.org/10.1056/nejmoa2107454.

(15) Qasim, W.; Zhan, H.; Samarasinghe, S.; Adams, S.; Amrolia, P.; Stafford, S.; Butler, K.; Rivat, C.; Wright, G.; Somana, K.; Ghorashian, S.; Pinner, D.; Ahsan, G.; Gilmour, K.; Lucchini, G.; Inglott, S.; Mifsud, W.; Chiesa, R.; Peggs, K. S.; Chan, L.; Farzeneh, F.; Thrasher, A. J.; Vora, A.; Pule, M.; Veys, P. Molecular Remission of Infant B-ALL after Infusion of Universal TALEN Gene-Edited CAR T Cells. *Sci. Transl. Med.* **2017**, *9* (374), 1–8. https://doi.org/10.1126/scitranslmed.aaj2013.

(16) Tebas, P.; Stein, D.; Tang, W. W.; Frank, I.; Wang, S. Q.; Lee, G.; Spratt, S. K.; Surosky, R. T.; Giedlin, M. A.; Nichol, G.; Holmes, M. C.; Gregory, P. D.; Ando, D. G.; Kalos, M.; Collman, R. G.; Binder-Scholl, G.; Plesa, G.; Hwang, W.-T.; Levine, B. L.; June, C. H. Gene Editing of CCR5 in Autologous CD4 T Cells of Persons Infected with HIV. *http://dx.doi.org/10.1056/NEJMoa1300662* **2014**, *370* (10), 901–910. https://doi.org/10.1056/NEJMOA1300662.

(17) Xu, L.; Wang, J.; Liu, Y.; Xie, L.; Su, B.; Mou, D.; Wang, L.; Liu, T.; Wang, X.; Zhang, B.; Zhao, L.; Hu, L.; Ning, H.; Zhang, Y.; Deng, K.; Liu, L.; Lu, X.; Zhang, T.; Xu, J.; Li, C.; Wu, H.; Deng, H.; Chen, H. CRISPR-Edited Stem Cells in a Patient with HIV and Acute Lymphocytic Leukemia. *N. Engl. J. Med.* **2019**, *381* (13), 1240–1247. https://doi.org/10.1056/nejmoa1817426.

(18) Huo, Y.; Aboud, K.; Kang, H.; Cutting, L. E.; Bennett, A. Clinical Endpoints for the Study of Geographic Atrophy Secondary to Age-Related Macular Degeneration. **2017**, *44* (9), 1–13. https://doi.org/10.1097/IAE.0000000000001283.Clinical.

(19) Lu, Y. C.; Parker, L. L.; Lu, T.; Zheng, Z.; Toomey, M. A.; White, D. E.; Yao, X.; Li, Y. F.; Robbins, P. F.; Feldman, S. A.; Van Der Bruggen, P.; Klebanoff, C. A.; Goff, S. L.; Sherry, R. M.; Kammula, U. S.; Yang, J. C.; Rosenberg, S. A. Treatment of Patients with

Metastatic Cancer Using a Major Histocompatibility Complex Class II-Restricted T-Cell Receptor Targeting the Cancer Germline Antigen MAGE-A3. *J. Clin. Oncol.* **2017**, *35* (29), 3322–3329. https://doi.org/10.1200/JCO.2017.74.5463.

(20) Chemello, F.; Chai, A. C.; Li, H.; Rodriguez-Caycedo, C.; Sanchez-Ortiz, E.; Atmanli, A.; Mireault, A. A.; Liu, N.; Bassel-Duby, R.; Olson, E. N. Precise Correction of Duchenne Muscular Dystrophy Exon Deletion Mutations by Base and Prime Editing. *Sci. Adv.* **2021**, *7* (18). https://doi.org/10.1126/sciadv.abg4910.
